# MiRNAs function in the development of resistance against doxorubicin in cancer cells: targeting ABC transporters

**DOI:** 10.3389/fphar.2024.1486783

**Published:** 2024-11-29

**Authors:** Xin-Yan Lu, Hongxu Jin

**Affiliations:** ^1^ Department of General Surgery, Shengjing Hospital of China Medical University, Shenyang, China; ^2^ Emergency Medicine Department of General Hospital of Northern Theater Command, Shenyang, Liaoning, China

**Keywords:** doxorubicin, ncRNA, miRNA - microRNA, chemoresisitance, ABC transporter

## Abstract

Resistance to chemotherapeutic agents poses a significant challenge in cancer treatment, particularly with doxorubicin, a widely used drug for various cancers, including breast cancer, leukaemia, osteosarcoma, and gastrointestinal cancers. This review aims to elucidate the critical role of microRNAs (miRNAs) in the development of doxorubicin resistance, focusing on their interactions with ATP-binding cassette (ABC) transporters. Despite extensive research, the molecular mechanisms governing doxorubicin resistance still need to be completed, particularly regarding the regulatory influence of miRNAs on ABC transporter expression. By analyzing current literature, this review identifies a notable gap: the lack of comprehensive insight into how specific miRNAs modulate the expression and activity of ABC transporters in cancer cells, contributing to doxorubicin resistance. We systematically examine recent findings on the interplay between miRNAs and ABC transporters, providing a detailed assessment of potential therapeutic strategies that leverage miRNA modulation to overcome drug resistance. Ultimately, this review underscores the significance of integrating miRNA research into existing therapeutic frameworks to enhance the efficacy of doxorubicin in cancer treatment.

## Graphical Abstract

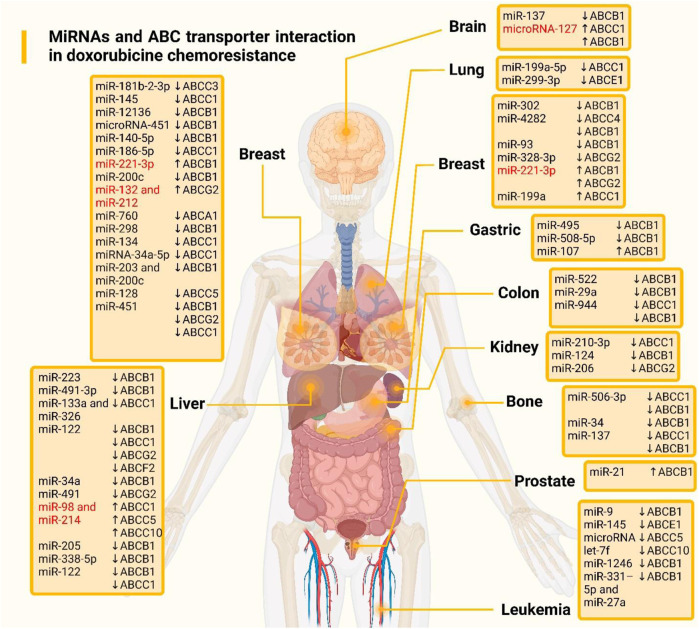

## 1 Introduction

Cancer, as the second leading reason for death around the world, is known to impose a great burden on the socioeconomic situations of every country, which is a reason for increasing the attention of different countries to the topic of cancer and increasing investment in providing suitable solutions to prevent and treat this deadly disease (Siegel et al., 2023). The past 20 years have witnessed the identification and development of more than 70 targeted therapeutic agents as novel drugs for treating a broad range of cancer types (Kroemer et al., 2023). These drugs combat cancers via influencing the proliferation, growth, survival, and metastasis of tumour cells, which is achieved by interfering with particular signalling pathways (Goossens and Bailly, 2019; Jairajpuri et al., 2024; Zhang et al., 2022). However, narrow indications have been extensively reported for these innovative therapeutic agents. Due to some restrictions, such as the heterogeneous nature of cancer, genomic instability, emergence of drug resistance, and many other unknown factors, the efficacy of these agents is diminished (Hays and Bonavida, 2019; Sansregret et al., 2018; Chen L. et al., 2023). As a result, cancer therapy is still dominated by conventional chemotherapy, which is a substantial mainstay and tool in fighting cancer. Again, the application of conventional chemotherapeutics faces two major problems worthy of attention, including the development of resistance and adverse effects (Sun et al., 2020; Nazari Soltan Ahmad et al., 2020; He et al., 2021). An increase in cellular detoxification, apoptosis inhibition, dysregulation of DNA repair, and regulation of non-coding RNAs (ncRNAs) are among the most noteworthy mechanisms responsible for the appearance of drug resistance (Aldossary, 2019). Therefore, the hot point of research in cancer treatment is finding novel candidates that not only reverse drug resistance by increasing the effectiveness of conventional therapeutic agents but also have low side effects due to their low toxicity on healthy cells (Wu et al., 2020).

Doxorubicin is a widely used chemotherapeutic agent, particularly effective in treating a variety of cancers, including breast, ovarian, and lung malignancies. However, the efficacy of doxorubicin is frequently compromised by the development of drug resistance, a major obstacle to successful cancer treatment (Kciuk et al., 2023). One of the most significant mechanisms driving doxorubicin resistance is the overexpression of ATP-binding cassette (ABC) transporters, which actively expel chemotherapeutic agents, such as doxorubicin, from cancer cells. ABC transporters, particularly ABCB1 and ABCC1, are widely expressed in multiple cancer types and are strongly implicated in multidrug resistance, which severely limits the effectiveness of chemotherapy. By increasing drug efflux, these transporters reduce the intracellular concentration of chemotherapeutic agents, thereby diminishing their cytotoxic effects. In hepatocellular carcinoma, for example, the inhibition of ABCB1 has been shown to increase doxorubicin accumulation, reduce drug tolerance, and improve treatment outcomes (Blukacz et al., 2024). Similarly, in triple-negative breast cancer (TNBC), the stabilization of ABCB1 via USP51-mediated deubiquitination enhances resistance to doxorubicin, while targeting USP51 impairs this resistance (Ou et al., 2024).

Even though approximately 80% of the human genome is transcribed into RNA, only about 2% of these RNAs are translated into proteins (Anastasiadou et al., 2018). Consequently, ncRNAs are RNA molecules that are transcribed from DNA but are not translated into proteins. They constitute the vast majority of cellular RNAs and play significant roles in regulating various physiological, biological, and pathological processes (Yan and Bu, 2021). Over the past few decades, there has been growing interest in exploring the involvement of key ncRNA categories, particularly microRNAs (miRNAs) and long non-coding RNAs (lncRNAs), in the initiation and progression of various human cancers. Highly conserved miRNAs are approximately 20-nucleotide-long non-coding RNAs, and they lack protein-coding potential (Hauptman and Glavač, 2013). Recently, miRNAs have been at the centre of debate regarding their crucial role in reversing chemoresistance. For example, miR-218-5p has been shown to inhibit doxorubicin-induced mitophagy by targeting Parkin, thereby increasing doxorubicin sensitivity in breast cancer cells (Naso et al., 2024). Additionally, miR-181b-5p reverses doxorubicin resistance by downregulating the p53/p21 pathway and inhibiting G1 arrest through the suppression of BCLAF1, as demonstrated *in vivo* (Zhao et al., 2023). Their role in cancer progression and treatment resistance is becoming increasingly evident, particularly with respect to their ability to influence the expression and activity of ABC transporters (Gomes et al., 2020). Among these, miR-34a-5p has garnered attention for its potent effect on ABCC1, a key transporter associated with doxorubicin resistance. Studies have demonstrated that the restoration of miR-34a-5p expression significantly suppresses ABCC1 by directly targeting its 3′ untranslated region, leading to increased doxorubicin influx in resistant breast cancer cells. This enhanced intracellular accumulation of doxorubicin effectively reverses the resistance phenotype, highlighting miR-34a-5p as a potential therapeutic candidate for overcoming ABC transporter-mediated drug resistance (Yahya et al., 2024). This study focuses on elucidating the role of miRNAs in the development of resistance to doxorubicin, with a particular emphasis on their regulatory effects on ABC transporters in cancer cells. By identifying specific miRNAs that modulate ABCB1 and ABCC1, we aim to provide a comprehensive understanding of how miRNA-mediated mechanisms contribute to chemoresistance. Our objective is to explore the potential of these miRNAs as therapeutic targets to overcome doxorubicin resistance, thus enhancing the effectiveness of conventional chemotherapy and improving clinical outcomes.

## 2 An overview of mechanisms underlying doxorubicin resistance

### 2.1 Doxorubicin mechanism of action

Doxorubicin is widely used in chemotherapy for its effectiveness against various cancers, though its exact mechanisms of action are still under debate. Despite this, it is known that doxorubicin intercalates into DNA, inhibits topoisomerase enzymes, and disrupts mitochondrial function, leading to increased production of free radicals and oxidative damage (Elfadadny et al., 2023). Doxorubicin primarily induces DNA damage through the formation of adducts, single-strand breaks, and double-strand breaks, trapping topoisomerase enzymes. These breaks activate the DNA damage response pathway, recruiting various repair proteins and initiating cell-cycle arrest or apoptosis. The drug’s ability to induce apoptosis is linked to phosphorylation events mediated by the ATM and ATR kinases, which further activate checkpoint kinases and tumour suppressor proteins like p53, ultimately halting cell proliferation and triggering programmed cell death (Halim et al., 2018). In addition to its apoptotic effects, doxorubicin can trigger other cell death mechanisms, such as senescence, autophagy, pyroptosis, ferroptosis, or necrosis, depending on drug dosage and cellular context (Christidi and Brunham, 2021). Doxorubicin’s interaction with cardiolipin in mitochondria increases levels of reactive oxygen species (ROS) and reactive nitrogen species, which contribute to DNA damage in both nuclear and mitochondrial DNA. This oxidative stress amplifies ROS production, further damaging the electron transport chain and reinforcing the cycle of ROS generation. Through interactions with proteins like p53 and pathways such as ceramide signalling, doxorubicin promotes the activation of proapoptotic factors like cytochrome c, APAF1, and SMAC/DIABLO, leading to the activation of caspases that finalize the apoptotic process by fragmenting DNA and executing cell death (Kciuk et al., 2023).

Furthermore, doxorubicin enhances anti-tumor immune responses, particularly by activating CD8^+^ T cells. Through the release of damage-associated molecules like ATP, calreticulin, and HMGB1, doxorubicin-treated cancer cells can be recognized by dendritic cells, leading to the activation of immune responses. These interactions promote T-cell and natural killer cell activity, which releases cytotoxic molecules that directly contribute to cancer cell destruction (Yang et al., 2021; Zhao et al., 2024). Moreover, doxorubicin-induced DNA damage activates pathways that may influence immune checkpoint regulation, such as upregulation of PD-L1, which underscores the potential for combining doxorubicin with immune checkpoint inhibitors in treatment strategies. The drug’s ability to stimulate both direct cytotoxicity and immune-mediated responses is crucial for its therapeutic effects (Sadighi et al., 2021).

### 2.2 Doxorubicin drug resistance

Doxorubicin is considered a legacy antitumor drug due to its long-standing use across a wide range of cancer types, including breast cancer, lymphoma, and sarcoma. Compared to other traditional chemotherapy drugs like cisplatin and vincristine, doxorubicin’s ability to target both DNA and mitochondrial function gives it a broader range of cytotoxic mechanisms, making it one of the most versatile anticancer agents (Sritharan and Sivalingam, 2021). Like other chemo drugs, doxorubicin resistance is mediated by the ability of cancer cells to overcome the initial cytotoxicity and restart the proliferative and invasive capabilities of these cells (Cox and Weinman, 2016). Given the rising incidence of drug resistance, it is crucial to investigate how cancer cells adapt by switching between different signalling pathways to sustain their proliferation and invasion, ultimately leading to the development of doxorubicin resistance. Inhibiting topoisomerase II activity, which leads to the induction of single- and double-strand DNA breaks, limitation of DNA replication and transcription, and finally, interfering with cancer cell proliferation is the proper mechanism for the cytotoxicity of doxorubicin (Cao X. et al., 2020). However, DNA damage alone is often insufficient to cause cancer cell death, as the DNA damage repair mechanisms in cancer cells can mitigate the cytotoxic effects of doxorubicin. Various pathways, including non-homologous end joining and homologous recombination, may repair the DNA breaks induced by doxorubicin, thus promoting survival and enabling resistance. Recent studies have highlighted that proteins involved in DNA repair, such as SIRT6, play a significant role in modulating doxorubicin sensitivity. SIRT6-mediated resistance was found to be associated with enhanced DNA damage repair activity, which could be attenuated by inhibiting this pathway using agents like olaparib or ATM inhibitors. Similarly, overexpression of SIRT6 in osteosarcoma cells has been linked to poor patient prognosis, further demonstrating the importance of DNA repair in doxorubicin resistance (Zhang et al., 2020). Moreover, thioredoxin-interacting protein (TXNIP), a key regulator of ROS synthesis, was found to promote doxorubicin-induced DNA damage in TNBC cells. TXNIP enhances ROS-mediated DNA damage, contributing to doxorubicin efficacy; however, the upregulation of TXNIP via inhibitors like c-Myc could potentially overcome resistance mechanisms (Chen et al., 2022a). Beyond the critical functions of various molecular pathways in determining the doxorubicin sensitivity of cancer cells, intracellular organelles like mitochondria are reported to play pivotal roles (Dai et al., 2017; Kirtonia et al., 2020). Cancer cell treatment with doxorubicin is extensively demonstrated to result in structural and functional alterations in mitochondrial genes. Since mitochondria are a central organelle that triggers apoptosis, any change in the mitochondria has a close association with apoptosis (Taymaz-Nikerel et al., 2018). Thus, the interplay between DNA damage repair, ROS production, and mitochondrial dysfunction underpins key aspects of doxorubicin resistance, where a multifaceted approach targeting both the DNA repair and mitochondrial pathways might provide enhanced therapeutic efficacy. The cell cycle, the main factor in cancer progression, is suppressed by doxorubicin application. However, prolonged exposure of cancer cells to doxorubicin leads to the development of resistance against this chemotherapeutic agent, which is mainly reported to be caused by a mutation in p53, a key tumour suppressor gene (Guo et al., 2023; Aas et al., 1996). Not surprisingly, cross-resistance between two drugs is also common in cancer therapy. For example, simultaneous exposure to doxorubicin and paclitaxel is demonstrated to lead to the mutation of p53 and the possession of resistance in hepatocellular carcinoma (HCC) cells (Chan and Lung, 2004). P53 mutation-mediated emergence of doxorubicin resistance is due to overexpression, as well as promoted activity of P-glycoprotein (P-gp), an ATP-dependent pump responsible for the effluxing doxorubicin out of cells into extracellular space. Due to the function of this pump, the intracellular levels of doxorubicin will decrease, and its accumulation within cells will be inhibited (Chan and Lung, 2004). However, in addition to these molecular pathways, several other mechanisms significantly contribute to doxorubicin resistance. For instance, cancer cell heterogeneity presents a major challenge, as the diverse genetic and phenotypic profiles within tumours enable certain subpopulations of cells to evade the cytotoxic effects of doxorubicin. Studies have shown that cancer cells cultured in three-dimensional extracellular matrix (ECM)-based models exhibit altered sensitivity to doxorubicin compared to cells grown in two-dimensional conditions, largely due to enhanced cell-to-ECM interactions. Inhibition of integrin signalling in combination with doxorubicin has been shown to significantly reduce cancer cell viability (Lovitt et al., 2018). Additionally, cancer stem cell (CSC) theory posits that a small subset of cancer cells within the tumour has the ability to self-renew and initiate tumour regrowth after therapy, contributing significantly to drug resistance. Doxorubicin treatment has been shown to induce a therapy-induced senescence state, during which a dormant population of cells can survive, later re-enter the cell cycle, and increase expression of stemness-related genes, such as SOX2, KLF4, and c-MYC. This senescence-associated stemness enhances the tumour’s aggressiveness and risk of recurrence, as demonstrated in liver cancer studies where EpCAM+/CD133+ stem cells exhibit increased resistance to doxorubicin due to elevated ABCG2 expression (Zhang et al., 2020; Karabicici et al., 2021). Epigenetic changes are another contributing factor to drug resistance. Aberrant methylation patterns, histone modifications, and chromatin remodelling can silence key genes involved in drug sensitivity, such as the MSH2 gene, which is important for DNA mismatch repair. Treatment with epigenetic modulators like 5-aza-deoxycytidine and trichostatin A has been shown to re-sensitize resistant breast cancer cells to doxorubicin (Ponnusamy et al., 2018).

Moreover, various molecular mechanisms have been identified as contributing to changes in the activity and expression of drug transporters, leading to the development of doxorubicin resistance. For example, doxorubicin accumulation can be prevented via nuclear factor erythroid 2-related factor 2 (Nrf2)-mediated enhancement in the activity and expression levels of ABCB1, another important drug transporter (Xia et al., 2020). These membrane-bound proteins, particularly members like P-gp (ABCB1), MRP1 (ABCC1), and BCRP (ABCG2), play pivotal roles in the efflux of doxorubicin and other chemotherapeutic agents out of cancer cells, thereby reducing intracellular drug concentrations and diminishing the efficacy of the treatment. This drug efflux function is a major adaptive response that enables cancer cells to evade the cytotoxic effects of doxorubicin, allowing them to continue increasing despite drug exposure.

Unlike doxorubicin, other well-known drugs like cisplatin primarily act by forming platinum-DNA adducts, and vincristine disrupts the microtubules in dividing cells, highlighting the unique multi-targeted cytotoxicity of doxorubicin. However, like doxorubicin, both drugs also face significant issues with the development of resistance, which emphasises the necessity of understanding and overcoming these mechanisms to improve the long-term efficacy of chemotherapies (Sohail et al., 2021). The following section will provide a detailed overview of ABC transporters, including their structure, function, and distribution, to aid in the comprehension of their critical role in the development of drug resistance in cancer therapy.

## 3 ABC transporters: structure, function, distribution

### 3.1 Structure

**FIGURE 1:**
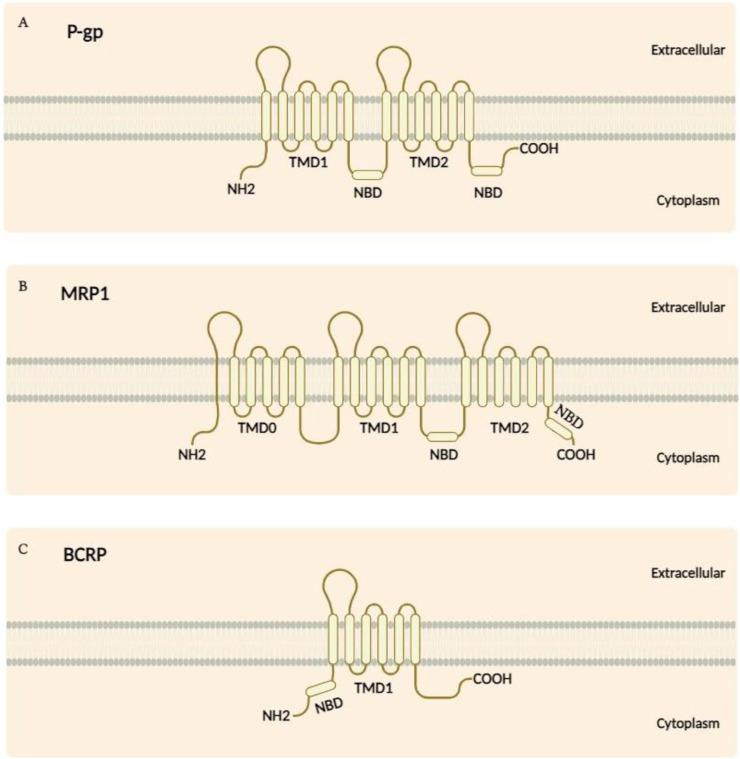
**(A)** The structure of P-gp consists of two NBDs situated in the cytoplasm, along with 12 transmembrane segments. **(B)** While MRP1 shares a similar structural topology with P-gp, it is distinguished by an additional TMD made up of five segments. **(C)** In contrast, the BCRP features an NBD located at the NH2 terminus and has a TMD composed of six segments.

ABC transporters represent one of the most extensive families of transmembrane proteins, distinguished by their conserved structural framework and vital functions in substrate transport across cellular membranes. These transporters are typically composed of transmembrane domains (TMDs) and nucleotide-binding domains (NBDs). As seen in Figure 1, although P-gp features two NBDs and a standard TMD arrangement, MRP1 differs by having an additional TMD comprising five segments. Similarly, BCRP presents a unique structure with its NBD situated at the NH2 terminus alongside a distinct TMD configuration. The TMDs are organised into multiple alpha-helical segments that span the membrane and create pathways for specific substrate translocation. On the cytoplasmic side, the NBDs contain essential motifs, including the Walker A and Walker B motifs, the ABC signature, and the Q-loop, which are crucial for ATP binding and hydrolysis. The energy released from ATP hydrolysis in the NBDs drives conformational changes in the TMDs, thereby facilitating the movement of substrates through the membrane (Alam and Locher, 2023; Huang and Ecker, 2023).

### 3.2 Function

**FIGURE 2:**
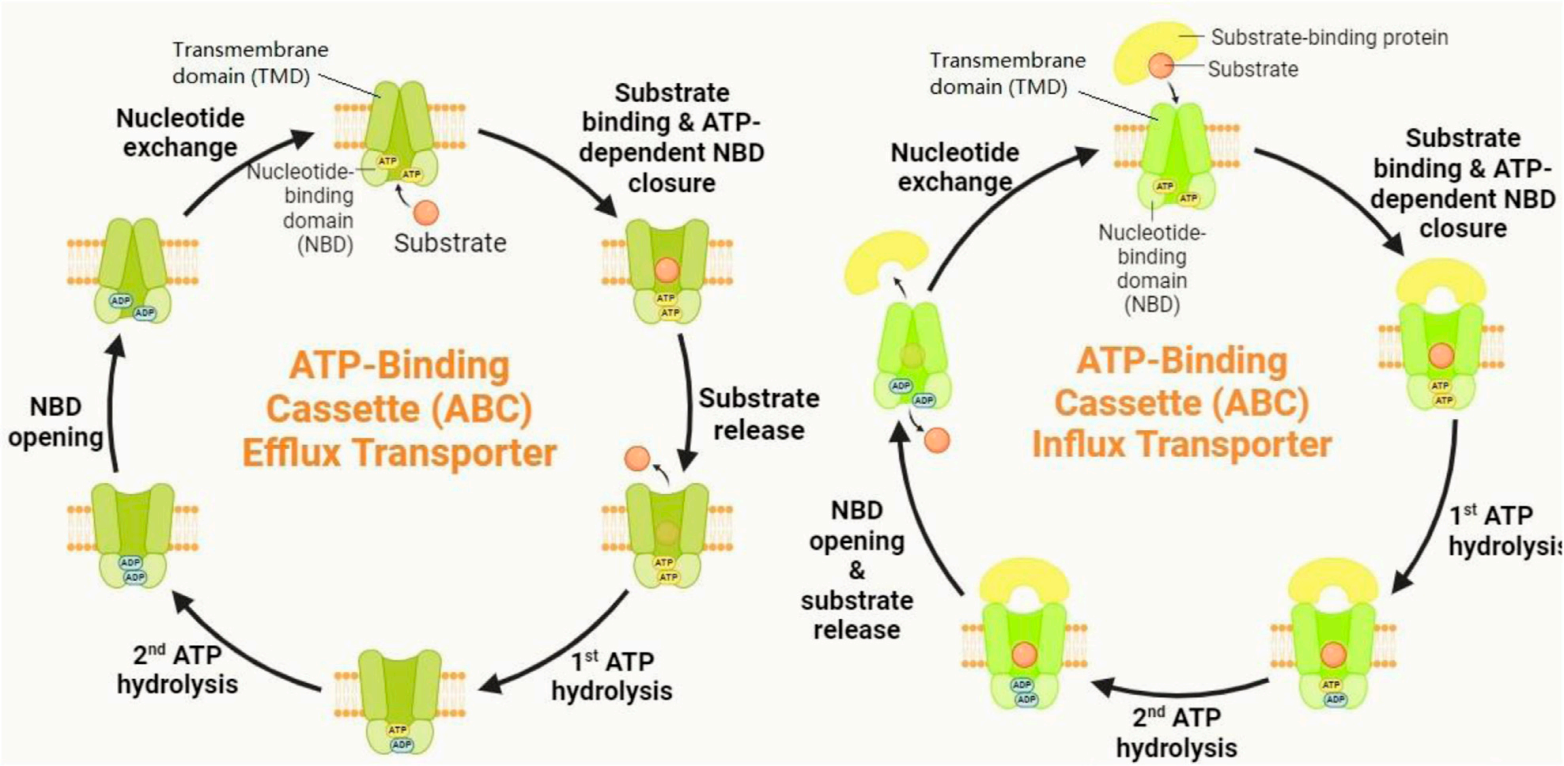
Mechanism of action of ABC transporters. ABC transporters operate through a sophisticated mechanism involving substrate binding and conformational changes. When a substrate binds to the high-affinity TMD pockets, it induces significant conformational shifts in the NBDs. This interaction results in the formation of closed NBD dimers. The closure of these NBD dimers facilitates ATP hydrolysis, which, in turn, drives the rotation of the TMDs. This rotation triggers conformational alterations in the TMDs, causing them to open toward the extracellular space. As a result, the substrate is expelled from the cell.

According to Figure 2, ABC transporters harness the energy from ATP hydrolysis to transport a wide variety of substrates, including lipids, ions, peptides, and xenobiotics, across biological membranes. Among the most extensively studied ABC transporters are ABCB1 (also known as P-glycoprotein), ABCC1 (also referred to as multidrug resistance protein 1, MRP1), and ABCG2 (also known as breast cancer resistance protein, BCRP) (Banerjee et al., 2023). ABCB1 is primarily known for its role in conferring multidrug resistance in cancer by specifically expelling chemotherapeutic agents out of cells, thereby abating intracellular drug accumulation and diminishing therapeutic efficacy (Chen H-K. et al., 2023). ABCC1, which belongs to the C subgroup of ABC transporters, similarly contributes to drug resistance by transporting a broad spectrum of substrates, including organic anions and drugs, out of cells. As a lipophilic anion pump, ABCC1 has more diversity in its substrates than P-gp. Amphipathic organic acids containing large hydrophobic groups are the most prevalent substrates of ABCC1 (Zhang et al., 2024). ABCG2, a member of the G subfamily, is another key player in multidrug resistance, with its property to efflux a wide variety of chemotherapeutic agents, as well as endogenous and exogenous substrates (Schulz et al., 2023).

Beyond their role in drug resistance, these transporters also participate in various physiological processes. ABCB1, for example, is crucial in protecting tissues like the blood-brain barrier by eliminating the admission of potentially harmful xenobiotics into the central nervous system. ABCC1 is implicated in transporting glutathione conjugates, bile acids, and other organic anions, playing a vital role in cellular detoxification. ABCG2 regulates the intracellular levels of ions, lipids, hormones, and organelles like the Golgi apparatus, endoplasmic reticulum, lysosome, and mitochondrion (Sajid et al., 2023).

### 3.3 Distribution

The distribution of ABC transporters is extensive and reflects their diverse functional roles. ABCB1 is highly expressed in the kidneys, liver, intestines, and the blood-brain barrier, where it is pivotal in the absorption, distribution, and elimination of pharmacological compounds and xenobiotics. Its expression in the blood-brain barrier is notably critical for protecting the central nervous system from toxic substances (Moore et al., 2023). ABCC1 is widely expressed in tissues consisting of the lungs, blood cells, and liver, where it is involved in the efflux of organic anions and drug conjugates. ABCG2 is expressed in the placenta, liver, intestine, and blood-brain barrier, where it helps protect these tissues from xenobiotics and facilitates the efflux of physiological substrates. The widespread expression of these transporters underscores their importance in maintaining cellular homeostasis, protecting tissues from toxic insults, and contributing to drug resistance, a significant obstacle in combating tumorigenesis (Leonard et al., 2003).

The regulation of ABC transporters extends beyond intrinsic cellular mechanisms and includes modulation by miRNAs, which have emerged as crucial post-transcriptional regulators in various physiological and pathological processes, including cancer drug resistance. Several studies have demonstrated that miRNAs can directly target the mRNA of ABC transporters such as ABCB1, ABCC1, and ABCG2, influencing their expression levels and activity. This regulation can significantly impact the efflux of chemotherapeutic agents, thus modulating drug resistance (Wang et al., 2021). Understanding the intricate relationship between miRNAs and ABC transporters is pivotal in identifying novel therapeutic strategies to counteract multidrug resistance in cancer cells. In the following section, we will explore the biogenesis and functional roles of miRNAs to facilitate the understanding of how their dysregulation contributes to drug resistance, particularly through their influence on ABC transporters.

## 4 miRNAs

### 4.1 Biogenesis and biology

Even though a vast majority of the human genome (around 80%) is transcribed into RNA, only about 2% of this RNA is translated into proteins. (Cai et al., 2009). Consequently, ncRNAs comprise the bulk of cellular RNAs and play a significant role in regulating physiologic and pathological processes (Ye et al., 2019). Over the past few decades, there has been a growing interest in studying the role of two key types of ncRNAs, miRNAs and long non-coding RNAs (lncRNAs), in the initiation and progression of divergent human cancers (Reddy, 2015; Xu et al., 2024). MiRNAs, a class of small ncRNAs consisting of 20–22 nucleotides, regulate crucial cellular processes by targeting the 3′ untranslated region (UTR) of mRNA in target genes. (Vishnoi and Rani, 2017; Ha and Kim, 2014). Primary-miRNAs (pri-miRNAs) with a poly-A tail and 5′ cap are the first product of RNA polymerase II during miRNA biosynthesis. Then RNAase III endonuclease, known as Drosha, converts pri-miRNA into precursor miRNAs (pre-miRNAs) with 60–110 nucleotide length (Achkar et al., 2016). The pri-miRNA is transferred into the cytoplasm, and in a reaction catalyzed by RNase III enzyme DICER-1, the stem-loop structures are removed, and the miRNA duplex is created (Romero-Cordoba et al., 2014). Following unwinding by a helicase, the mature single-stranded miRNA is incorporated into the RNA-induced silencing complex (RISC), where it participates in the negative regulation of target mRNAs (Romero-Cordoba et al., 2014; Michlewski and Cáceres, 2019). Dysregulation in the expression pattern of miRNAs contributes to the disruption of various cellular events and, ultimately, maltransformation (Lin and Gregory, 2015; Zhou et al., 2022). Hence, these tiny RNA molecules are of high importance in the initiation/progression of various human malignancies (Lin and Gregory, 2015). More importantly, miRNAs are also present in biological fluids comprising saliva, urine, plasma and serum, which increase their value as biomarkers. miRNAs are released into body fluids through various mechanisms, including passive release and excretion via microvesicles or exosomes (Hayes et al., 2014). A huge amount of miRNAs is excreted into extracellular space via an RNA-binding protein-dependent pathway due to the miRNAs association with RNA-binding proteins and proteins high-density lipoprotein (HDL), Argonaute-2 (Ago-2), Nucleophosmin 1 (Npm1), and exosome (Selvarajan et al., 2019).

### 4.2 miRNAs and chemoresistance

MiRNAs are pivotal in modulating apoptosis, and the programmed cell death process is often disrupted in cancer. By targeting pro-apoptotic and anti-apoptotic genes, miRNAs can tilt the balance toward cell survival or death in response to chemotherapy. For instance, miR-519d enhances cisplatin-induced apoptosis in breast cancer stem cells by downregulating MCL-1, a pro-survival factor. This demonstrates how miRNAs can potentiate the effects of chemotherapeutic agents by promoting apoptosis, thereby reducing the likelihood of drug resistance (Xie et al., 2017). Furthermore, miRNAs play a significant role in epithelial-mesenchymal transition (EMT), a critical process that allows cancer cells to gain migratory and invasive properties, contributing to metastasis and drug resistance. By targeting key transcription factors and signalling molecules, miRNAs can influence EMT. For example, miR-451a targets c-Myc, a regulator of EMT, thereby enhancing the sensitivity of lung cancer cells to doxorubicin. In this way, miRNAs can modulate the EMT process and affect tumour aggressiveness and response to therapy (Tao et al., 2020; Ashrafizadeh et al., 2024).

The ability of miRNAs to regulate the cell cycle is also vital in determining the responsiveness of cancer cells to chemotherapeutic agents. Specific miRNAs can induce cell cycle arrest by targeting cyclins and cyclin-dependent kinases (CDKs). For instance, miR-221/222 targets ANXA3, leading to cell cycle arrest in breast cancer cells, which sensitizes them to adriamycin. This highlights the potential of miRNAs as therapeutic targets to enhance drug sensitivity by managing cell proliferation (Kim et al., 2023). The regulation of CSCs also underscores the importance of miRNAs in chemoresistance. CSCs possess unique properties that contribute to tumour recurrence and therapy resistance. MiRNAs play a crucial role in regulating the self-renewal and differentiation of CSCs. For instance, the enforced expression of miR-519d has been shown to increase sensitivity to cisplatin in T-47D cancer stem cells, indicating that miRNAs can modify CSC behaviour and enhance therapeutic efficacy. By targeting pathways that govern CSC characteristics, miRNAs may offer new strategies for overcoming drug resistance (Xie et al., 2017). The regulation of the DNA damage response is another critical area where miRNAs exert their influence. By targeting genes involved in DNA repair mechanisms, miRNAs can affect the ability of cancer cells to withstand DNA-damaging therapies. For example, miR-140s inhibition of FEN1 not only suppresses DNA repair but also enhances the response to chemotherapy, demonstrating how miRNAs can shape the cellular response to DNA damage (Lu et al., 2020).

Crucially, miRNAs also play a direct role in regulating ABC transporters, which are critical for drug efflux and resistance. ABC transporters, such as ABCC1, MRP-7, and others, are membrane proteins that pump out various substrates, including chemotherapeutic agents, thereby reducing their efficacy. For instance, miR-133b has been shown to target ABCC1 directly, reducing its expression and enhancing chemosensitivity in drug-resistant colorectal cancer cells. *In vivo* studies have demonstrated that restoring miR-133b levels in resistant cancer models led to decreased tumour growth, corroborating the notion that miRNAs can effectively modulate transporter activity and influence treatment outcomes (Chen et al., 2017). Similarly, miR-98 inhibits MRP-7, a transporter associated with paclitaxel resistance. By repressing MRP-7 expression, miR-98 enhances the sensitivity of cancer cells to paclitaxel, providing a potential strategy for overcoming drug resistance in endometrial cancer. These findings underscore the potential of miRNAs to serve as therapeutic agents or adjuvants, providing a novel approach to sensitize tumours to chemotherapy (Huang et al., 2021).

By influencing the expression and function of ABC transporters, miRNAs serve as vital regulators of drug resistance in cancer. Understanding their role in this context not only sheds light on the mechanisms of chemoresistance but also opens avenues for therapeutic interventions aimed at restoring sensitivity to chemotherapy. Future research focusing on miRNAs as potential biomarkers and therapeutic targets may lead to more effective strategies for combating drug-resistant cancers and improving patient outcomes. This comprehensive understanding of miRNA-mediated regulation of ABC transporters could pave the way for the development of innovative combination therapies that exploit these molecular insights to enhance the efficacy of existing chemotherapeutic regimens.

## 5 miRNAs targeting drug transporters in doxorubicin resistance

A key mechanism driving the chemotherapeutic resistance, including doxorubicin, is the upregulation of drug transporters from the ATP-binding cassette family (Xiao et al., 2021). Among these, P-gp, MRP1, and BCRP play central roles in mediating drug efflux, leading to decreased intracellular drug concentrations and reduced cytotoxicity (Xiao et al., 2021; Dean et al., 2001). P-gp is particularly well-known for its broad substrate specificity, effectively transporting a range of hydrophobic drugs such as doxorubicin, vinblastine, daunorubicin, and vincristine. This transporter significantly contributes to drug resistance by actively exporting these chemotherapeutics out of cancer cells, thereby reducing their efficacy (Bogman et al., 2001).

On the other hand, MRP1, a lipophilic anion pump, transports amphipathic organic acids, including a wide variety of chemotherapeutics such as etoposide, Vinca alkaloids, irinotecan (SN-38), and anthracyclines like doxorubicin and daunorubicin. Although there is some overlap in drug resistance phenotypes between P-gp and MRP1, they differ in substrate specificity, with MRP1 being more versatile (Hanssen et al., 2021). MRP1 has been identified as a key player in cells lacking P-gp expression, where it takes over the efflux of chemotherapeutics, further contributing to resistance. Studies using MRP1-transfected cells demonstrate that MRP1 significantly impacts drug resistance in multiple cancer types, including leukaemia, gastric, lung, HCC, and breast cancers. In these malignancies, the overexpression of both P-gp and MRP1 has been associated with diminished cytotoxic effects of doxorubicin, underscoring their importance in modulating drug response (Park et al., 2004; Carabias et al., 2022; Chen et al., 2016; Kong et al., 2020; Yu et al., 2021).

In addition to P-gp and MRP1, other ABC transporters such as MRP6 (ABCC6), MRP2 (ABCC2), MRP7 (ABCC10), and MRP3 (ABCC3) also contribute to the development of drug resistance in various cancers. (Kruh and Belinsky, 2003). Importantly, BCRP has emerged as a critical transporter implicated in the efflux of doxorubicin and other chemotherapeutic agents, further compounding resistance mechanisms (Szakács et al., 2006). To combat this, several miRNAs have been shown to target these ABC transporters, particularly P-gp, MRP1, and ABCG2, thereby altering the sensitivity of cancer cells to doxorubicin. These miRNAs serve as potential modulators in overcoming drug resistance, offering promising therapeutic avenues for reversing the refractory nature of cancer cells to chemotherapeutic agents like doxorubicin which have been specifically investigated in the following sections.

### 5.1 Breast cancer

Breast cancer is now the second most prevalent aggressive carcinoma among women, and globally accounts for the fourth most common cancer-related death (Bray et al., 2024). Doxorubicin is widely considered the most impactful chemotherapeutic drug in addressing breast cancer. However, its potency is often compromised by the emergence of multidrug resistance in breast cancer cells during chemotherapy. Approximately 30%–50% of patients with metastatic breast cancer respond positively to doxorubicin treatment (Zangouei et al., 2021). While multiple mechanisms contribute to drug resistance in cancer cells, the most thoroughly investigated is drug efflux. This process is mediated by overexpressed ABC transporters on the cell membrane, which actively pump anticancer drugs out of the cells, thus diminishing their effectiveness (Yahya et al., 2024). As a result, the development of reliable and efficient inhibitors targeting ABC transporters to triumph over MDR has become a critical preference in breast cancer therapy.

In breast cancer, doxorubicin is a fundamental chemo-drug substance to eliminate cancer cells. However, resistance to doxorubicin, often mediated by P-gp, is a common challenge that reduces the effectiveness of treatment and lowers patient survival rates (Lao et al., 2013). Several miRNAs have been marked as crucial players in this resistance by targeting P-gp. For instance, miR-200c, an acclaimed tumour suppressor miRNA in breast cancer, exhibits a marked downregulation in MCF-7 breast cancer cells compared to their drug-sensitive counterparts. This downregulation is associated with escalated P-gp expression, suggesting that overexpression of miR-200c could potentially mitigate P-gp levels and thereby enhance the sensitivity of breast cancer cells to doxorubicin (Chen et al., 2012).

Additionally, Armada et al. demonstrated that both miR-200c and miR-203 negatively regulate the activity of P-gp, effectively reversing doxorubicin resistance in breast tumour cells (Armada et al., 2019). Another study by Bao et al. highlighted that miR-298 is significantly repressed in doxorubicin-resistant breast cancer cell lines, where it strongly contributed to the upregulation of P-gp. This miRNA directly binds to the 3′ UTR of P-gp, suppressing its expression and overcoming doxorubicin resistance (Bao et al., 2012). Similarly, miR-12136 has been shown to attach to the 3′ UTR region of ABCB1 mRNA, reducing P-gp levels and reversing doxorubicin resistance (Yuan et al., 2020). Moreover, miR-124-3p and miR-451 are other anti-oncogenic miRNAs that sensitize breast cancer cells to doxorubicin by targeting P-gp (Kovalchuk et al., 2008; Hu et al., 2019). Elevated levels of miR-129-5p in doxorubicin-resistant MCF-7 cells have been observed to trigger intracellular accretion of doxorubicin, achieved by inhibiting P-gp activity and expression. Additionally, miR-129-5p suppresses CDK6, bringing about cell cycle arrest in the G2 phase, thereby reversing doxorubicin resistance (Yi et al., 2016). The regulatory network between miRNAs and competing endogenous RNAs (ceRNAs) is a key mechanism that involves lncRNAs in maintaining the functional balance between critical signalling pathways in cancer. Disruption of the miRNA-ceRNA network can have severe consequences. For example, miR-221-3p, a tumour suppressor miRNA in breast cancer, has several conserved binding sites with the lncRNA GAS5, indicating that GAS5 may function as a ceRNA for miR-221-3p. Chen et al. found that GAS5 is upregulated, while P-gp is downregulated in doxorubicin-resistant breast cancer samples and cell lines. GAS5 is crucial in modulating resistance to doxorubicin by simultaneously targeting P-gp and miR-221-3p while also engaging the Wnt/β-catenin signalling pathway (Chen et al., 2020). Another miRNA, miR-21, indirectly regulates P-gp expression in MCF-7 cells via the hyaluronan-CD44-mediated protein kinase C (PKC) pathway (Bourguignon et al., 2009). Furthermore, the miR-302 family (miR-302a/b/c/d) has been proven to sensitize breast cancer cells to doxorubicin by suppressing P-gp through the inhibition of MAP/ERK kinase kinase 1 (MEKK1) (Zhao et al., 2016).

Beyond the well-established role of P-gp, MRP1 is critically involved in developing resistance to doxorubicin (Louisa et al., 2014). MiRNAs have come to light as key regulators in reversing this resistance by targeting these proteins. Notably, miR-145, a tumour suppressor miRNA, has been revealed to enhance the sensitivity of breast tumour cells to doxorubicin by specifically targeting MRP1. *In vivo* studies corroborate these findings, demonstrating that increased intracellular accumulation of doxorubicin and suppression of MRP1 significantly improve the response to doxorubicin chemotherapy (Gao et al., 2016; Wuxiao et al., 2020). Similarly, research by Lu et al. revealed a marked downregulation of miR-134 in doxorubicin-resistant MCF-7 cell lines. The introduction of a miR-134 mimic in these cells dramatically inhibited their proliferation and robustly triggered apoptosis, further underscoring the role of miR-134 in reversing doxorubicin resistance through MRP1 suppression (Lu et al., 2015). Comparable outcomes were observed in DOX-resistant MDA-MB-468 triple-negative breast cancer cells, where miR-186-5p effectively overcame doxorubicin resistance by targeting MRP1 (Lu et al., 2022). Additionally, the miR-199a/MRP1 axis has also been entangled in the mechanism of doxorubicin resistance in breast cancer cells (Chang et al., 2018).

Several microRNAs have been identified as key regulators of the response of breast cancer cells to doxorubicin by influencing ABCG2 expression. Specifically, miRNA-132 and miRNA-212 are upregulated in doxorubicin-resistant breast cancer cells, a phenomenon associated with decreased PTEN expression, heightened AKT activation, and elevated NF-κB levels. Silencing these miRNAs reversed these molecular alterations and reduced ABCG2 expression, hence refining the sensitivity of resistant breast cancer cells to doxorubicin (Xie M. et al., 2018). Supporting these findings, Cano et al. identified a correlation between ABCG2 expression levels and miR‐99a‐5p in triple-negative breast cancer (TNBC) cells (Garrido-Cano et al., 2022). Upregulation of miR‐99a‐5p increased doxorubicin cytotoxicity by promoting apoptosis in MDA‐MB‐231 and MDA‐MB‐231R cells. Crucially, this effect was mediated by miR‐99a‐5p’s suppression of ABCG2, leading to significant doxorubicin accumulation and enhanced cytotoxicity in cancer cells (Garrido-Cano et al., 2022). Additionally, Pan et al. demonstrated that miR-221-3p downregulated homeodomain-interacting protein kinase 2 (HIPK2) while upregulating Che-1, a direct HIPK2 target, thereby reducing ABCG2 expression. These interactions contributed to the reversal of doxorubicin resistance, a finding corroborated by both *in vivo* and *in vitro* studies (Pan et al., 2021).

Owing to the intricate nature of miRNAs, directly targeting them to trigger or hamper their transcriptional activity might be sophisticated; as a result, finding substances that regulate their gene expression or their upstream regulators has drawn the growing attention of researchers to widen understanding of the drug resistance and eventually impeding it. Currently, growing evidence suggests that polyphenols like curcumol and quercetin have played an integral role in intensifying the cytotoxic ability of doxorubicin in breast malignancies (Zeng et al., 2020). Curcumol, the principal active component in Rhizoma Curcumae, possesses significant biological tasks and plays an imperative role in enhancing the efficacy of chemotherapy in various diseases (Shaikh et al., 2021). It has been displayed that curcumol boosts the chemotherapeutic influence of doxorubicin in breast cancer cells. Notably, curcumol was evidenced to significantly inhibit the mRNA and protein levels of MRP3 by upregulating miR-181b-2-3p (Zeng et al., 2020). Quercetin, a naturally occurring flavonoid, has been noted to reduce doxorubicin resistance caused by ABC transporters. However, its clinical application is constrained by its limited solubility. To achieve the necessary concentration for overcoming drug resistance, high doses of quercetin are required, which can lead to cytotoxic effects on healthy cells (Almohammad Aljabr et al., 2024). 7-O-Geranylquercetin (GQ), a liposoluble derivative of quercetin, has demonstrated the ability to suppress growth and stimulate apoptosis in human gastric, lung, and breast cancer cells (Liao et al., 2015; Zhu et al., 2017; Lotfi et al., 2023). Notably, GQ can reverse doxorubicin resistance in breast cancer cells at lower concentrations, with a more potent effect than quercetin, highlighting its potential as an effective MDR reversal agent. It has been shown that combining GQ with miR-451 significantly enhances the hindering effects of doxorubicin on the invasion and proliferation of MCF-7/DOX cells. This combination also reduces the expression levels of MDR1 and P-gp in these breast cancer cells. In a xenograft tumour model, the use of GQ and miR-451 together was found to amplify the tumour suppressor effects of doxorubicin in nude mice. Further analysis using Western blotting and immunohistochemistry confirmed a decrease in P-gp expression in cancerous tissues. More precisely GQ could upregulate miR-451 and they work synergistically to overcome drug resistance in MCF-7/DOX cells by downregulating MDR1 and P-gp expression (Chen et al., 2022b).

The intricate interplay between miRNAs, proteins, and key signalling pathways is a crucial focus for advancing strategies to overcome doxorubicin resistance in breast cancer. Future research should delve deeper into how miRNAs regulate both upstream and downstream effectors of ABC transporters, particularly P-gp and MRP1. For instance, miRNAs like miR-200c and miR-298 downregulate P-gp by targeting critical upstream signalling molecules such as the Wnt/β-catenin and PI3K/AKT pathways, influencing downstream effects on drug efflux. Similarly, miR-221-3p’s regulation of ABCG2 via the HIPK2-Che-1 axis provides insights into broader pathway involvement in drug resistance. Natural compounds like curcumol and 7-O-Geranylquercetin (GQ) show the potential to enhance this regulatory network by modulating miRNAs and pathways like the MAPK/ERK and CDK6 signalling cascades. By doing so, they influence both the upstream regulation of miRNA expression and the downstream suppression of drug-resistance proteins. Combining miRNAs with these polyphenols has demonstrated synergistic effects, particularly in upregulating tumour suppressor miRNAs such as miR-451 and targeting P-gp more effectively. To fully exploit these interactions, future studies should prioritize the investigation of key protein players and pathways governing the miRNA-ABC transporter axis, aiming to develop multi-targeted approaches that enhance doxorubicin sensitivity and circumvent drug resistance at multiple molecular levels. Incorporating nanotechnology and advanced drug delivery systems will also be vital in optimizing the bioavailability of such therapies and ensuring precise targeting, minimizing side effects and maximizing efficacy in clinical settings.

### 5.2 Colorectal cancer

Colorectal cancer (CRC), a prevalent disease in the modern era, claims nearly 700,000 lives annually and ranks as the fourth most lethal cancer globally (Kuipers et al., 2015; Siegel et al., 2014). Despite significant advancements in diagnostic methods and therapeutic strategies, the outlook for CRC patients prevails unfavourable due to challenges in early detection and the manifestation of drug resistance (Ashique et al., 2024). Doxorubicin is commonly administered to eliminate residual CRC cells post-surgery and in progressed stages of CRC (Xiong and Xiao, 2018). However, despite its extensive and long-standing clinical use, the performance of doxorubicin is narrowed by the emergence of drug resistance.

Accumulating evidence has emerged on ABC transporters’ role and their interaction with miRNAs as a potential bypass for doxorubicin chemoresistance in CRC. However, miRNAs like miR-522 directly target the activity of ABCB5, and some others such as miR-944 and miR-29a could recruit the more complicated mechanisms and interact with protein regulators and signaling pathways to exert their role (Xi et al., 2021; Shi et al., 2020; Yang et al., 2015). ABCB5 plays a critical role in cancer outgrowth, particularly in CRC, where it has been established as a vital element in mediating patient resistance to doxorubicin chemotherapy (Guo et al., 2018). Its involvement in tumor growth is further supported by studies utilizing shRNA to knock down ABCB5 expression in colorectal cancer cell lines, which resulted in reduced tumor formation in human-to-mouse xenograft models (Wilson et al., 2011). These findings highlight the multifaceted contributions of ABCB5 to cancer advancement and resistance to medical treatment, alluding to its potential as a target for combination therapy. Fascinatingly, miRNA target prediction algorithms pinpointed ABCB5 as a promising target of miR-522. This was confirmed through a fluorescent reporter assay, which verified that miR-522 specifically binds to the predicted site within the 3′-untranslated region of ABCB5 mRNA. Overexpression of miR-522 in HT29/DOX cells brought about decreased levels of ABCB5 protein levels, suggesting that miR-522 mimics could be a promising treatment approach for overcoming chemoresistance in colorectal cancer (Yang et al., 2015). In a recent study, the interaction of CirRNAs/miRNAs was suggested to be associated with doxorubicin CRC cell resistance. Silencing circCSPP1 increased sensitivity to DOX, reduced cell growth and invasion, and triggered apoptosis by modulating the miR-944/FZD7 axis in DOX-resistant CRC cells, offering new therapeutic potential for CRC treatment (Xi et al., 2021). FZD7, a member of the Frizzled family, is instrumental in tumour metastasis by modulating both non-canonical and canonical Wnt signalling pathways (Larasati et al., 2022). FZD7 is crucial in various cancers, including CRC. Previous studies have shown that knocking down FZD7 reduces cellular viability and invasive potential in CRC (Hafezi et al., 2022). Elizabeth et al. showed that the expression of FZD7, which β-catenin/TCF4 regulates, is reduced at the invasive front of colorectal cancer, suggesting that FZD7 promotes CRC progression (Vincan et al., 2010).

Interestingly, Xi and colleagues unravelled that circCSPP1 directly augmented the expression of FZD7 level by reversing the suppressive effect miR-944 on FZD7 transcription, thereby potentiating the capability of P-gp, MRP1, and LRP to abate the cytotoxic property of doxorubicin (Xi et al., 2021). In another investigation, phosphatase and tensin homolog (PTEN)/phosphoinositide 3-kinase (PI3K)/Akt axis has been found to exert the interplay role between miR-29a and P-gp (Shi et al., 2020). PTEN, a tumour suppressor gene located at 10q23.31, encodes a protein with diverse functions, including phosphatase activity targeting protein substrates such as focal adhesion kinase (FAK), insulin receptor substrate 1 (IRS-1), protein tyrosine kinase 6 (PTK6), and phosphatidylinositol-3,4,5-trisphosphate (PIP3) (Chawra et al., 2024). The loss or deletion of a single PTEN allele is sufficient to trigger the PI3K/Akt/PKB axis, feasibly giving rise to the development of colon cancer. PTEN exerts its inhibitory effect on the PI3K/Akt signalling through PIP3 dephosphorylation to produce PIP2, thereby reducing cell proliferation, promoting apoptosis, and decreasing invasiveness (Salvatore et al., 2019). Conversely, suppression of PTEN leads to PKB activation, which in turn phosphorylates and activates various proteins, including mTOR, GSK3β, Bad, caspase 9, IKK, and P-gp. Within the context of P-pg, it has been demonstrated that PTEN protein level was significantly lessened in HT29/DOX cells; however, miR-29a led to the amplified expression of PTEN in these cells. The upregulation of PTEN in HT29/DOX cells subsequently suppressed the PI3K/Akt signalling, ultimately resulting in the downregulation of P-gp and increased drug amassment within the cells (Shi et al., 2020). These conclusions indicate that modulating miR-29a expression could serve as a valuable predictor of clinical response to HT29/DOX, offering a promising therapeutic target for colon cancer treatment.

In light of the emerging role of miRNAs and their interactions with ABC transporters, future therapeutic strategies for overcoming doxorubicin resistance in CRC should prioritize a deeper exploration of these molecular mechanisms. Targeting key regulators such as ABCB5 through miRNAs like miR-522 presents a promising avenue, particularly for addressing drug resistance. However, the complexity of these interactions, including the influence of circRNAs and other signalling pathways like the PI3K/Akt axis, suggests that a multi-targeted approach may be necessary for more effective treatment outcomes. The potential of miRNA mimics or inhibitors to modulate resistance mechanisms opens the door to novel combination therapies, integrating miRNA-based interventions with conventional chemotherapeutics. Furthermore, advancing the understanding of the broader implications of miRNA-mediated regulation—such as their effects on apoptosis, EMT, and cancer metastasis—could enhance the specificity of targeted treatments. Future research should also explore how the modulation of miRNAs like miR-29a and circRNAs, such as circCSPP1, can be utilized to re-sensitize resistant CRC cells, providing a personalized therapeutic strategy.

### 5.3 Gastric cancer

Gastric cancer is positioned as the second primary cause of cancer-related fatality universally, with minimal advancements in prolonged survival rates over the last decade (Iwu and Iwu-Jaja, 2023). Chemotherapy remains the fundamental treatment approach for gastric malignancy; nevertheless, the effectiveness of chemotherapy is significantly hindered by MDR (Hosseini et al., 2024). Numerous studies have identified that MDR is rooted in alterations in medication absorption, secretion, and metabolism, as well as changes in DNA replication, repair mechanisms, cell survival, apoptosis, and other biological cellular process. Recent research has shed light on the molecular underpinnings of MDR, particularly focusing on non-coding RNAs like miRNAs (Duan et al., 2023).

**FIGURE 3:**
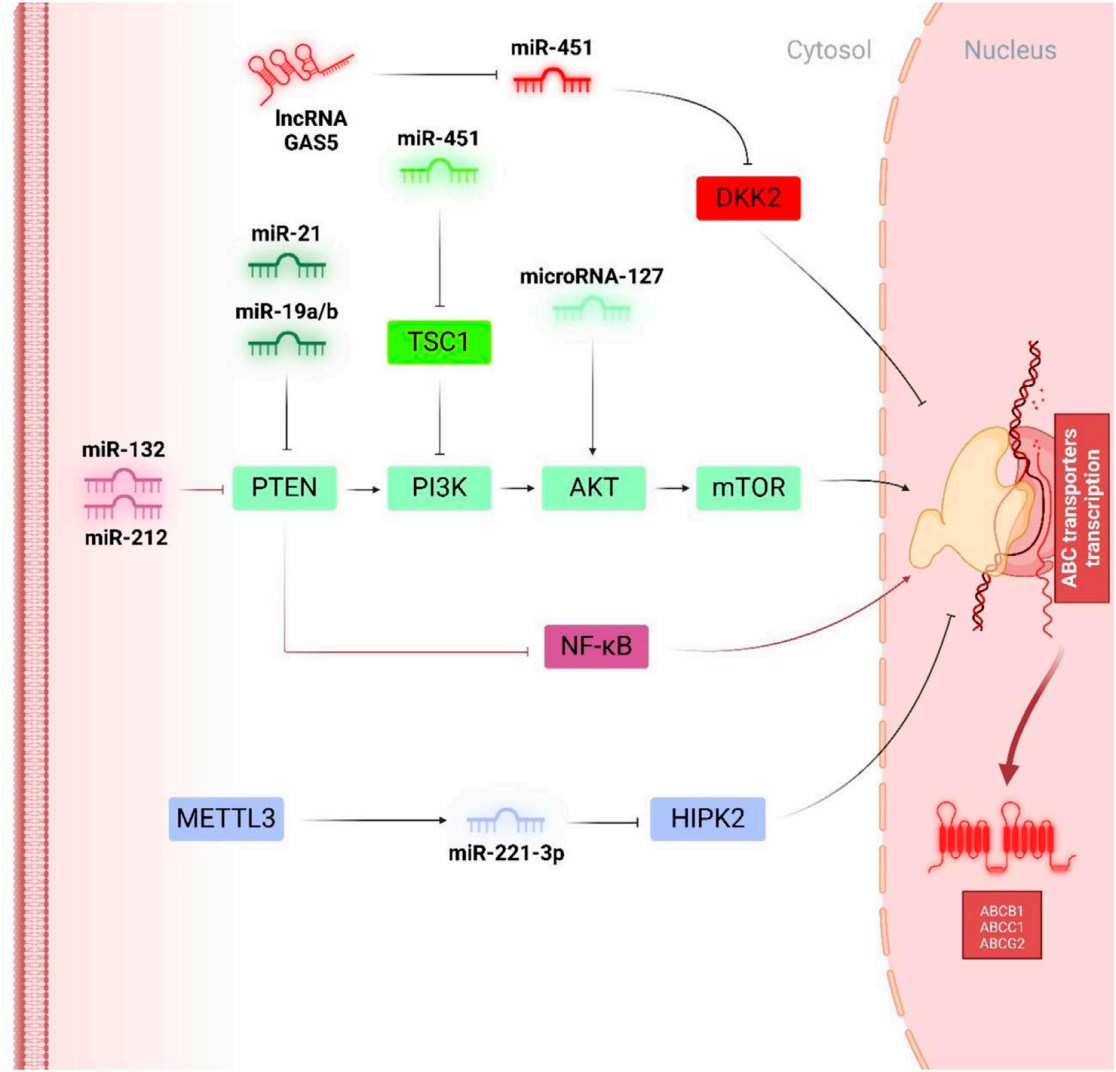
Tumor promotor miRNAs in regulation of doxorubicin chemoresistance. PTEN, as a multi-functional tumour suppressor, has been widely downregulated by oncogenic miRNAs like miR-21 and miR-19a/b to augment the phosphorylation of PI3K/Akt/mTOR pathways and facilitate the expression of ABCB1. On the other hand, miR-451 and miR-127, independent of PTEN activity, influence the phosphorylation of PI3K and Akt, respectively, to enhance ABCB1 and ABC1-mediated doxorubicin chemoresistance. Interestingly, miR-132 and miR-212 inhibited PTEN expression to trigger AKT phosphorylation and the NF-κB pathway and consequently led to an increment of ABCG2.

In gastric malignancies, miR-19a/b has been recognized as the tumour promotor miRNA aids in drug resistance, and on the contrary, miR-107, miR-508-5p, and miR‐495 have been presented as the anti-oncogenes acting in an ABC transporter-dependent condition (Shang et al., 2014; Teng et al., 2015; Zou et al., 2017; Wang et al., 2013). Research has indicated that miR-19a/b enhances MDR in gastric cancer cells by facilitating the expulsion of chemo-drug agents and suppressing drug-related apoptosis. Notably, akin to miR-29a in colorectal cancer, miR-19 contributes to the onset of doxorubicin resistance in human gastric cancer cells through its targeting of PTEN. As illustrated in Figure 3, it has been validated that PTEN is an efficient downstream of miR-19a/b in gastric tumour cells, displaying that miR-19a/b aids in alleviating the susceptibility to doxorubicin by downregulating the PTEN and subsequent phosphorylation of AKT, contributing to the suppression of Bax and Caspase-3 together with an increment of the cytotoxic drug efflux by intensifying the expression of P-pg (Wang et al., 2013). Regarding the other interplay regulators, zinc ribbon domain-containing 1 (ZNRD1) is the other one contributing to the miRNAs-mediated regulation of ABC transporters. ZNRD1, a transcription-associated gene, encodes a protein characterized by two zinc ribbon domains. Similar zinc ribbon motifs have been observed in various other transcription-related proteins, suggesting that ZNRD1 may participate in the regulation of gene expression (Sansregret et al., 2018; Chen L. et al., 2023; Sun et al., 2020). Recent findings indicated that ZNRD1 expression is correlated with multidrug resistance in gastric cancer through the modulation of P-pg. These observations suggest that the ZNRD1 gene may be involved in transcriptional modulation and could play significant roles in moderating both physiologic and pathological processes in gastric cancer (Hong et al., 2005; Shi et al., 2004). In this context, the study by Shang et al. demonstrated both direct and indirect regulation of P-gp by miR-508-5p. Specifically, miR-508-5p overexpression led to a reduction in P-gp and ZNRD1 levels by directly addressing their 3′-UTRs. Additionally, miR-508-5p’s inhibition of ZNRD1 resulted in decreased P-gp transcription. Consequently, reversing the P-gp-related doxorubicin resistance phenotype may be partially explained by a regulatory feedback loop involving miR-508-5p, ZNRD1, and P-gp. The newly identified miR-508-5p/P-gp/ZNRD1 regulatory network offers fresh insights into the mechanisms driving doxorubicin chemoresistance. Restoring miR-508-5p expression could emerge as a promising therapeutic approach for combating MDR in gastric cancer in the future (Shang et al., 2014).

Focusing on the upstream regulators of miRNAs may offer new paves to combat the gastric cancer chemoresistance mechanism (Liu et al., 2021a). Considering the RNA-binding protein as one of the most familiar upstream regulators of miRNAs and upon the intricate RNA-binding protein/miRNA interaction, the Lin28/miR-107 axis has been the centre of debates regarding their tumour promotor or oncogenic effects, thus unravelling the RNA-binding protein-miRNA axis is significant for determining targetability in gastric chemoresistance. An *in vivo in vitro* study indicated that following transfection with Lin28, there was a marked reduction in miR-107 expression, whereas knockdown of Lin28 led to an upregulation of miR-107 levels. Additionally, 48 h post-transfection with pre-miR-107, a significant decrease in Lin28 RNA levels was observed, and even 96 h post-transfection, Lin28 protein levels remained lower than those in the non-transfected control group. Further investigations revealed that Lin28 could suppress the miR-107, leading to the upregulation of C-myc and P-gp and the downregulation of Cyclin D1, which collectively contribute to the subtraction of the cytotoxicity-inducing ability of doxorubicin on gastric tumour cells (Teng et al., 2015).

In summary, combining new molecularly targeted therapies with conventional chemotherapy might overcome MDR more effectively. For example, using agents that inhibit the function of miR-19a/b alongside doxorubicin could potentially improve response rates and reduce resistance. Therapies targeting ZNRD1, manipulating RNA-binding protein levels or their interaction with miRNAs, and other key regulators identified in the miRNA regulatory loops might be developed to address specific resistance mechanisms. Future strategies to combat gastric cancer will likely involve a combination of targeted molecular therapies, personalized treatment approaches, and innovative drug delivery systems. As research progresses, these approaches will provide more effective ways to manage and potentially overcome MDR in gastric cancer.

### 5.4 Osteosarcoma

Doxorubicin resistance is also a problem worthy of attention in patients with osteosarcoma (Armstrong and Dass, 2018). Therefore, some studies tried to use miRNAs to reverse doxorubicin resistance. For example, Yang et al. examined the effects of miR-125b-5p on cell growth, apoptosis, metastasis, and doxorubicin resistance in osteosarcoma cell lines. Osteosarcoma cells transfected with miR-125b-5p showed higher sensitivity to doxorubicin, which was achieved by downregulation of P-pg drug transporter and suppression of signal transducer and inhibits the transcription 3 (STAT3) expression (Xu et al., 2013). STAT3 activation, acting as a convergence point for multiple oncogenic signalling pathways, can lead to the abnormal transcription of genes involved in tumour cell growth, apoptosis, and resistance to chemotherapy. This process contributes to tumour advancement and is correlated with a poor prognosis in patients. In cases of osteosarcoma, miR-221 and miR-506-3p have been found to exhibit an opposite effect on STAT3 activity and influence P-pg expression. Liu et al. announced that miR-221 was intensified in doxorubicin-resistant Saos-2 cells (Liu et al., 2021b). Treatment of cells with miR-221 inhibitor prompted a significant subside in the expression levels of STAT3, P-gp, and Bcl-2. In addition, the STAT3 inhibitor, STAT3-IN-3, also attenuated miR-221 mediated P-gp upregulation in Saos-2 cells. As a result, miRNA-221 was suggested to elevate the P-gp and Bcl-2 expression by stimulating the STAT3 pathway to foster DOX resistance in osteosarcoma cells (Liu et al., 2021b). In contrast to the tumour promotor activity of miR-221, a recent study unveiled that miR-506-3p involved Janus kinase 2 (JAK2) in controlling the STAT3 activity during doxorubicin chemoresistance. JAK2 is a tyrosine kinase positioned beneath the cell membrane, capable of interacting with various cytokine receptors to trigger the implementation of transcription factors, such as STAT3. STAT3, an intranuclear transcription factor, is essential in promoting cellular growth, differentiation, immune responses, and programed cell death. In typical physiological states, the JAK2/STAT3 signalling pathway plays a crucial role in regulating various fundamental biological processes, including embryonic development, immune regulation, and cellular progression and differentiation. Nevertheless, in specific pathological conditions, this pathway can become abnormally stimulated. When cytokines bind to their respective receptors, associated JAKs are recruited and subsequently phosphorylated, leading to JAK activation. This activation triggers the formation of docking sites for STAT proteins, which are then phosphorylated to produce p-STAT. These phosphorylated STATs separate from the receptor, dimerize, and move into the nucleus, where they interact with DNA to regulate gene transcription, thus activating the JAK/STAT signalling pathway. Research has confirmed that miR-506-3p can counteract chemoresistance to doxorubicin in U-2OS osteosarcoma cells by inhibiting JAK2 phosphorylation and lowering total STAT3 protein levels in drug-resistant cells. Consequently, this inhibition of the overactive JAK2/STAT3 pathway results in alleviated mRNA expression of P-pg, MRP1, Bcl-2, and Survivin (Wang et al., 2024).

Furthermore, circRNAs like circPVT1 add an additional layer of complexity to the regulatory network by modulating miRNA function. These circRNAs function as molecular sponges, binding to specific miRNAs, which in turn affects gene expression and contributes to the emergence of drug resistance. CircPVT1, originating from the PVT1 locus within the long noncoding RNA region on chromosome 8q24, has been identified as a contributor to chemotherapy resistance in various cancers. Additionally, elevated levels of circPVT1 have been observed in osteosarcoma, where it has been imputed to enhance the resistance of osteosarcoma cells to doxorubicin (Yi et al., 2023; Wang B. et al., 2022). In fact, CircPVT1 influences the transcriptional activity of miR-137 to regulate TP53-regulated inhibitor of apoptosis 1 (TRIAP1) expression, known for its role as a programmed cell death inhibitor, has recently been recognized as a key contributor to drug resistance in multiple cancer types. TRIAP1 was detected as a direct target of miR-137 and was found to partially counteract the enhanced sensitivity to DXR induced by miR-137 overexpression. Furthermore, mechanistic studies revealed that inhibition of miR-137 could partially reverse the decrease in TRIAP1 levels caused by circPVT1 silencing in DXR-resistant osteosarcoma cells. This finding supports the capability of circPVT1 as a molecular sponge for miR-137, thereby promoting the upregulation of TRIAP1 expression and consequently facilitating the transcription of *MRP1 and* P-gp (Li et al., 2021a; Adams et al., 2015).

In light of the mTOR pathway’s upregulation observed in numerous chemoresistant cancers, sirolimus (rapamycin), a first-class mTOR inhibitor, has been revealed to augment apoptosis in human osteosarcoma cells and enhance their responsiveness to anticancer drugs, potentially through the regulation of miRNAs. Sirolimus has been found to downregulate the transcriptional activity of PAK1 and P-gp by upregulating miR-34b. In essence, miR-34b inhibits the expression and function of P-gp by directly targeting the 3′-UTR of ABCB1 mRNA, leading to the repression of its mRNA (Zhou et al., 2016).

**FIGURE 4:**
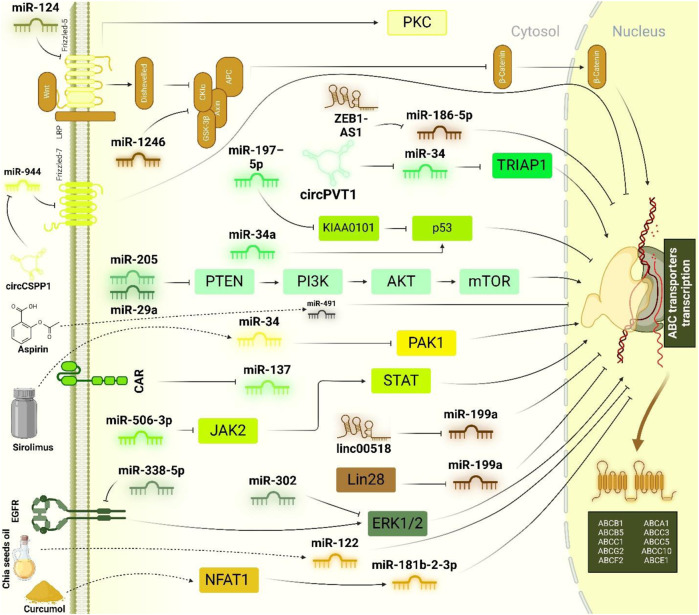
Tumor suppressor miRNAs in regulation of doxorubicin chemoresistance. miRNAs play a pivotal role in modulating doxorubicin chemoresistance, particularly by influencing several key signalling pathways. Among these, the PTEN/PI3K/Akt/mTOR, EGFR/ERK1/2, and Wnt/β-catenin pathways are notably regulated by miRNAs. The PTEN/PI3K/Akt/mTOR pathway has emerged as a primary target, with miR-205 and miR-29a identified as key inhibitors that reduce ABCB1 expression. Similarly, the EGFR/ERK1/2 axis, another critical pathway, can be reversed through the actions of miR-338-5p and miR-302, leading to the suppression of ABCB1. Furthermore, miR-124 and miR-1246 mitigate ABCB1 expression by inhibiting the Wnt/β-catenin pathway. In the context of the complex interplay between lncRNAs and miRNAs within ceRNA networks, linc00518 and ZEB1-AS1 have been identified as sponges for miR-199a and miR-186-5p, respectively, thereby promoting ABCC1 transcription. Additionally, several upstream regulators have been discovered that enhance the expression of miRNAs involved in the suppression of ABC transporters. Notably, natural compounds like chia seed oil and curcumol, alongside pharmacological agents such as aspirin and sirolimus, have been shown to upregulate miR-122, miR-181b-2-3p, miR-491, and miR-34, contributing to the downregulation of ABC transporters.

As illustrated in Figure 4, the identification of miR-125b-5p, miR-221, and miR-506-3p as modulators of the STAT3 pathway underscores the significance of targeting signalling cascades central to chemoresistance. Future studies should focus on the precise mechanisms through which these miRNAs interact with other molecular players, such as circRNAs like circPVT1, to refine their roles in resistance. Investigating combination therapies that include miRNA modulation alongside JAK2/STAT3 inhibitors could further optimize treatment strategies. Moreover, given the role of circRNAs as miRNA sponges, a deeper understanding of their interactions with miRNA-regulated networks, such as the influence of circPVT1 on miR-137, could lead to novel therapeutic interventions targeting multidrug resistance proteins like P-gp and MRP1. Additionally, mTOR inhibitors, such as sirolimus, present another dimension of therapeutic potential by modulating miRNA expression to overcome resistance, suggesting that future work should explore the synergy between miRNA-based therapies and mTOR inhibition. Together, these insights emphasize the need for further research to translate these molecular insights into clinically effective strategies, with the ultimate goal of improving treatment outcomes for patients with osteosarcoma.

### 5.5 Ovarian cancer

Although less studied, the functional characteristics and expression of ABC transporters in ovarian cells could potentially impact ovarian function and contribute to ovarian resistance by affecting the intracellular concentrations of hormones, drugs, or other signalling molecules pumping out chemotherapeutics, reducing their effectiveness. Zou et al. described that miR-495 upregulation in A2780DX cells (ovarian cancer cell line with doxorubicin resistance) led to significant downregulation of MDR1 and subsequent doxorubicin accumulation in the resistant cells, as well as apoptosis induction, which all demonstrated increased doxorubicin sensitivity. The authors concluded that administering miR-495 as a pre-treatment prior to chemotherapy may enhance the effectiveness of treatment against doxorubicin resistance mediated by P-gp. in ovarian cancer (Zou et al., 2017).

Recently, growing attention has been given to the intricate molecular correlation between NRF2 and cell signalling for ovarian cancer cell resistance. NRF2 is a transcription factor belonging to the cap’n’collar family of basic leucine-zipper (CNC-bZIP) proteins; it plays a central role in regulating the expression of divergent target genes, such as those involved in detoxification, antioxidant defence, and drug efflux. In numerous cancerous cells, there is frequent overexpression of NRF2, leading to a prompt formation of xenobiotic detoxifying enzymes and redox-regulating proteins. This elevation provides tumour cells with enhanced protection against anticancer drugs, apoptotic triggers, and radiotherapy (Li et al., 2021b). In the case of doxorubicin-resistant cancer cells, restriction of NRF2 expression has been observed to suppress c-MET/EGFR levels by upregulating miR-206. The NRF2-silencing-induced increase in miR-206 also leads to a reduction in BCRP expression, thereby enhancing chemosensitivity to anticancer treatments. These results offer new awareness of the molecular interactions between the cellular protective element NRF2 and the c-MET/EGFR oncogene signalling pathway, underscoring the pioneering role of miR-206 in reducing BCRP action (Choi et al., 2017).

The interplay between ABC transporters, microRNAs, and the NRF2 signalling pathway presents a promising avenue for enhancing therapeutic strategies against ovarian cancer, particularly in the context of drug resistance. The role of miR-495 in downregulating P-gp expression exemplifies the potential of targeting specific microRNAs to sensitize resistant ovarian cancer cells to doxorubicin. Furthermore, the intricate relationship between NRF2 and c-MET/EGFR signalling highlights the necessity of understanding how these molecular pathways converge to influence drug efflux and apoptosis. Future research should focus on developing targeted therapies that leverage these molecular interactions, such as the administration of miR-495 or the silencing of NRF2, to improve drug accumulation in resistant ovarian cells. Additionally, exploring the role of other regulatory microRNAs in modulating ABC transporter expression could unveil novel targets for combination therapies, ultimately leading to more effective treatment regimens for ovarian cancer patients. By advancing our understanding of these complex mechanisms, we can pave the way for innovative approaches that enhance chemotherapeutic efficacy and overcome resistance in ovarian cancer.

### 5.6 Leukemia

In reality, it is critical to emphasize that based on the previous investigation in leukaemia, until now, miRNAs have often displayed a tumour suppressor ability, directly influencing the transcriptional activity of ABC transporters. In chronic myelogenous leukemia (CML), Li et al. scrutinized the relationship between miR-9 and doxorubicin resistance in K562 cell lines, as well as patient samples representing resistance to doxorubicin. Both *in vitro* and *in vivo* investigations showed the sufficiency of miR-9 overexpression in reversing doxorubicin resistance and increasing the sensitivity of resistant cells to doxorubicin-mediated apoptosis. Further examinations have revealed the participation of P-gp in miR-9-related doxorubicin resistance. This protein is a direct target of miR-9 (Li et al., 2017). The protein expression of miR-331–5p and miR-27a were inversely associated with the transcription of the drug resistance factor P-gp in leukaemia cell lines exhibiting progressively increased resistance. P-gp has been identified as a target of both miR-331–5p and miR-27a, with the predicted binding sites of these miRNAs playing a vital role in regulating P-gp action. Additionally, at the protein level, it has been demonstrated that transfecting K562-resistant cells with miR-331–5p, miR-27a, or a combination of both resulted in reduced P-gp levels, with the combined miRNA treatment showing greater inhibitory effects than either miRNA alone (Feng et al., 2011).

Align with the previous research, the study of Xie stepped forward and indicated despite the direct effect of miRNAs on P-gp expression, some other signalling pathways, like as the Wnt/β-catenin pathway, might be involved during doxorubicin chemoresistance in leukaemia. The chemo-resistant properties of MDR leukaemia cells are diminished by the suppression of miR-1246, which modulates AXIN2 and GSK-3β, leading to the deactivation of the Wnt/β-catenin signalling pathway. In leukaemia cells, miR-1246 directly influences the 3′UTR seed-matching sites of AXIN2 and glycogen synthase kinase 3 beta (GSK-3β), worthwhile Wnt/β-catenin pathway participants. Inhibiting miR-1246 results in the upregulation of AXIN2 and GSK-3β, which afterwards deactivates the Wnt/β-catenin pathway. This process also reduces β-catenin expression, which in turn downplays P-gp levels in chemo-resistant leukaemia cells. These findings Intimate that miR-1246 could serve as a unique diagnostic biomarker and offer a latent strategy for overcoming drug resistance in leukaemia therapy (Xie et al., 2021).

Even though in most tumorigenesis and doxorubicin drug resistance, P-gp is the pioneer reprehensive of ABC transporters targeted by miRNAs, in leukaemia, some other little-known members comprising of ABCE1, ABCC5, and ABCC10 have been inferred in this process. In K562/ADM cells overexpressing miR-145, ABCE1 mRNA and transcriptional levels were notably reduced, while transfection with an miR-145 inhibitor led to a substantial increase in these levels. This suggests that miR-145 influences the 3′ UTR of ABCE1, promoting apoptosis in leukemic stem cells and enhancing the K562/ADM cells’ sensitivity to Adriamycin by suppressing ABCE1 (Wuxiao et al., 2020). The most contemplative case in ABC transporter-related chemoresistance may affiliated with ABCC5 and ABCC10, where they exhibited antiapoptotic and proliferative properties except for the drug efflux ability in leukemia doxorubicin resistance cells. Flow cytometry and Western blot assays conducted on K562 cells demonstrated that the higher expression of ABCC5 and ABCC10 resulted in increased levels of caspase-3 and PARP, while the levels of cleaved caspase-3 and cleaved PARP were reduced. Moreover, compared to cells transfected with let-7f, the co-overexpression cell lines exhibited a significant increase in colony formation, along with a decrease in the quantity of cells in the G1 phase. This was accompanied by a downregulation of p27 and upregulation of cyclin D1 and phosphorylated pRb, suggesting that the overexpression of ABCC5 and ABCC10 facilitates cancer cell growth. miRNA Let-7f was shown to negatively control the expression of ABCC5 and ABCC10, which in turn intensified cell sensitivity to doxorubicin, augmented apoptosis, and hindered cell proliferation (Cao YX. et al., 2020).

miRNAs have the utmost importance in overcoming chemoresistance in leukaemia by targeting ABC transporters like P-gp and others, such as ABCE1, ABCC5, and ABCC10. These miRNAs, including miR-9, miR-331–5p, miR-27a, and miR-1246, have been shown to increase the sensitivity of leukaemia cells to chemotherapy by modulating drug resistance pathways, including Wnt/β-catenin. Future research should focus on fully mapping miRNA-ABC transporter networks, understanding the interplay with other signalling pathways, and developing effective miRNA-based therapies. Personalized medicine approaches could tailor these therapies to individual patients, while clinical validation is needed to bring miRNA-targeted treatments into practice.

### 5.7 Glioma

Glioma, a prevalent form of neurological cancer, has emerged as a significant public health concern in modern society. Currently, the primary treatment for malignant glioma involves surgical removal, supplemented by postoperative radiotherapy and chemotherapy. Despite these approaches, treating glioma remains complex, with chemotherapy resistance in glioma cells posing a substantial challenge to effective clinical management. Feng et al. assessed the involvement of miR-127 in the doxorubicin resistance in U251 and U87-MG glioma cells. They found that both doxorubicin-resistant cells treated with miR-127 inhibitor showed higher sensitivity to doxorubicin. miR-127 silencing led to significant suppression of P-gp expression levels, as well as modulation of major apoptosis mediators, including Runx2, p53, bcl-2 and survivin. These results showed the efficacy of miR-127 downregulation in triggering apoptosis and overcoming doxorubicin resistance in glioma cells (Feng and Dong, 2015).

Identifying miR-137 and its regulatory role in ABC transporter expression, particularly P-gp, opens the door to developing therapies that can specifically modulate this pathway. Future research may focus on developing small molecules or gene-editing techniques to correct the hypermethylation of miR-137 or to inhibit CAR activity, potentially overcoming drug resistance in neuroblastoma. Future therapies might include miR-127 mimics or inhibitors to modulate drug resistance in glioma cells. In both neuroblastoma and glioma, a deeper comprehensive of the molecular interaction underlying drug resistance will be crucial for developing innovative treatments and improving patient outcomes.

### 5.8 Lung cancer

Lung cancer is primarily categorized into two histological subtypes relying on clinicopathological characteristics: non-small cell lung cancer (NSCLC) and small cell lung cancer (SCLC), with SCLC accounting for roughly 15% of all cases (Kratzer et al., 2024). SCLC is notably responsive to both radiotherapy and chemotherapy; however, the onset of drug resistance results in high relapse rates and poor clinical outcomes (Ying et al., 2024). Doxorubicin is commonly incorporated into chemotherapy protocols for treating SCLC patients. Despite chemotherapy achieving response rates exceeding 50%, the median overall survival (OS) is under 2 years because of the significant potential of disease relapse and progression, with just 15% of patients achieving long-term survival with limited disease (Lally et al., 2007; Das et al., 2021). Although doxorubicin is effective in its anti-tumor activity, the emergence of chemoresistance represents a significant challenge in SCLC treatment and is a critical factor in addressing the poor prognosis associated with this cancer (Hamilton et al., 2024). From a molecular perspective, it has been discovered that the miR-299-3p/ABCE1 axis could operate as a novel therapeutic target for addressing chemoresistance in SCLC. miR-299-3p exhibited reduced expression in both doxorubicin-resistant and--sensitive lung cancer samples. Through luciferase assay, it was observed to directly target the 3′-UTR region of the ABCE1 in lung cancer H69 cells. Notably, similar to the role of ABCC5 and ABCC10 in modulating proliferation and apoptosis in leukaemia, ABCE1 knockdown significantly suppressed H69/ADR cell growth, augmented the cell cycle inhibition rate, and promoted apoptosis. Furthermore, miR-299-3p markedly lowered ABCE1 protein levels, contributing to an antiproliferative environment and enhancing cell death, thereby reversing doxorubicin resistance in cancerous cells and thereupon providing novel approaches into the biological property mediated by miR-299-3p (Cao YX. et al., 2020; Zheng et al., 2015).

NSCLC constitutes over 85% of lung cancer cases and is often recognized at a progressed stage (Raskova Kafkova et al., 2024). Chemotherapy is a key therapeutic approach for NSCLC, but the appearance of resistance to anticancer drugs poses a substantial challenge to effective treatment. The DNA-damaging agent doxorubicin has shown an overall response rate of only 30%–50% in treating NSCLC. Clinical studies suggest that co-administration of doxorubicin with supplementary chemotherapeutic agents can offer benefits to NSCLC patients. Doxorubicin works by inhibiting the relegation of DNA strands during double-strand breaks (DSBs), thereby arresting tumour progression and inducing programmed cell death. However, drug resistance can severely constrain the effectiveness of doxorubicin in treating NSCLC (Kciuk et al., 2023; Maharati and Moghbeli, 2023; Moyal et al., 2018). Studies have established that miR-199a-5p is notably downregulated in cells resistant to doxorubicin, and its overexpression can enhance doxorubicin sensitivity in these resistant cells. Among the diverse targets of miR-199a-5p, increased expression of MRP1 and HIF-1α is associated with chemoresistance. Functional studies, including gain- and loss-of-function experiments, have substantiated that ABCC1 and HIF-1α contribute to the chem-drug resistance observed in NSCLC cells. miR-199a-5p downregulates MRP1 and HIF-1α by directly attaching to their 3′UTRs regions in NSCLC (Jin et al., 2020). Even more, in an investigation by LV et al., it was shown that miR-155 inhibition in doxorubicin-resistant A549 cell line resulted in recovery of cell cycle arrest and doxorubicin-induced apoptosis, hence reversal of doxorubicin resistance. Further investigations have revealed that this effect of miR-155 was mediated via suppression of P-gp, glutathione S-transferase-π, Survivin and Bcl-2, and upregulation of the expression of caspase-3 and caspase-8. Moreover, Akt and NF-kb proteins were also downregulated upon miR-155 downregulation in A549 cells with doxorubicin resistance. Therefore, miR-155 was indicated to participate in the doxorubicin resistance in lung cancer cells through targeting the P-gp/Akt/NF-kb pathway (Lv et al., 2016).

In summary, while doxorubicin remains a cornerstone in the treatment of lung cancer, both SCLC and NSCLC demonstrate significant challenges due to the development of chemoresistance. The discovery of miR-299-3p and miR-199a-5p as crucial regulators in mediating resistance provides valuable insights for future therapeutic strategies. Targeting these microRNAs could lead to innovative approaches to reverse drug resistance and improve clinical outcomes for lung cancer patients. For instance, enhancing the expression of miR-299-3p may restore sensitivity to doxorubicin in resistant SCLC cells by downregulating ABCE1, thus inhibiting cell growth and promoting apoptosis. Similarly, leveraging the pathways regulated by miR-199a-5p could provide a dual approach in NSCLC, addressing the expression of MRP1 and HIF-1α to improve doxorubicin efficacy. Future research should focus on the potential of combination therapies that incorporate miRNA modulation alongside conventional chemotherapeutic agents. This could pave the way for more personalized treatment regimens that enhance sensitivity to existing drugs while mitigating resistance mechanisms, ultimately leading to improved survival rates and quality of life for patients suffering from lung cancer.

### 5.9 Prostate cancer

Prostate cancer is now the most prevalent diagnosed noncutaneous malignancy in developed nations and ranks as the second predominant cause of cancer-related deaths among men (Wang L. et al., 2022). A substantial body of evidence indicates that intrinsic or acquired drug resistance is a principal barrier to accomplished treatment. This resistance is often attributed to MDR, which is commonly linked with the overexpression of ABC transport proteins (Ebrahimnezhad et al., 2024). The increased protein level of these efflux pumps restricts the intracellular concentration of chemotherapeutic agents like doxorubicin, thereby diminishing their therapeutic efficacy (Packeiser et al., 2023). The most extensively explored of these proteins are the P-gp and MRP1 transporters, which are minimally recruited in normal prostate tissue but show elevated expression as the tumour progresses (Sarwar et al., 2024). Zhou et al. disclosed robustly higher transcriptional levels of miR-21 in doxorubicin resistance prostate cancer PC3 cells. miR-21 downregulation attributed to significant palliation of resistant cell viability and induction of apoptosis. In cells transfected with miR-21 inhibitor, a dramatic decrease in the expression levels and activity of P-gp hence accumulation of doxorubicin in resistant cells was observed (Zhao et al., 2021). Strikingly, it gets more interesting to mention that akin to oncogenic miR-132, miR-212 in breast cancer (Xie M. et al., 2018), tumour promotor miR-29a in CRC (Shi et al., 2020), miR-21 mediated upregulation of P-gp in prostate cancer is a PTEN phosphatase-dependent circumstance. miR-21 significantly hindered the PTEN expression levels to exert the triggering condition on P-gp transcription, suggesting the therapeutic value of miR-21 in prostate cancer cells with doxorubicin resistance (Zhao et al., 2021). These findings have given rise to the notion of the influence of oncogenic miRNAs on PTEN and its relevance for the pathophysiology of doxorubicin resistance in chemotherapy, thereby pointing alluring directions for upcoming exploration.

### 5.10 HCC

HCC is the most widespread type of primary liver cancer and is classified as the third leading motive of cancer-related fatality rate on a global scale (Toh et al., 2023). HCC typically arises from chronic liver conditions, such as cirrhosis resulting from hepatitis B and/or hepatitis C infections, non-alcoholic steatohepatitis, iron overload disorders, obesity, autoimmune hepatitis, alcohol consumption, smoking, exposure to aflatoxins, and oral contraceptive utilization. Recent research indicates that Liver cancer can arise from either the dedifferentiation of mature hepatocytes or the impaired maturation of hepatic stem cells (Gabbia and De Martin, 2024; Feng et al., 2024). Regrettably, the prognosis for HCC remains unfavourable, largely due to the repetitive emergence of resistance to a range of chemo-drug agents. This resistance enables cancer cells to continue proliferating unchecked, leading to increased tumour aggressiveness and a higher potential for metastasis to other organs. Similar to other malignancies, elevated expression of ATP transporter proteins, which simplify the ATP-driven outflow of chemotherapeutic agents from cancerous cells, has been entangled in the acquisition of HCC chemoresistance, and more importantly miRNAs have been highlighted in reversing this mechanism through translational inhibition or mRNA degradation and deadenylation (Ladd et al., 2024; Lei et al., 2024).

As seen in Supplementary Table S1, even though miRNAs such as miR-223 (Yang et al., 2013), miR-133a, and miR-326 (Ma et al., 2015) particularly target one type of ABC transporters, some other ones comprising miR-122 (Yahya et al., 2018), miR-98, and miR-214 (Hamed et al., 2018) modulate the expression of multi-transporters simultaneously, making them efficient therapeutic agents reversing doxorubicin associated drug resistance in HCC. Amplified expression of miR-122 in HepG2 cells, both in the absence and presence of doxorubicin treatment, has the potential to influence the cells’ responsiveness to chemotherapy by reducing the transcription of multidrug resistance-associated genes, specifically P-gp and ABCF2 (Yahya et al., 2018). ABCF2, which is deficient in transmembrane domains and therefore cannot function as a classical transporter, is likely to act as a transcription factor involved in signalling pathways that are crucial for preventing drug resistance at metastatic sites and within the endocrine pathways of breast cancer. Although the exact molecular mechanisms are not yet fully understood, the contribution of ABCF2 to drug resistance in tumour cells is still unclear (Vasiliou et al., 2009). In the research conducted by Yahya et al., the levels of ABCF2 gene expression were found to be lower in cells treated with miR-122 mimics in contrast to those treated solely with acute doxorubicin or with a combination of acute doxorubicin and miR-122 mimics; notably, the lowest expression levels were perceived in the latter group. This observation suggests that miR-122 might be instrumental in downregulating ABCF2 gene expression. Noteworthy, HepG2 cells transfection with miR-122 mimics results in the arrest of the cell cycle and thus elevates the cells’ sensitivity to doxorubicin by diminishing the expression of multiple drug resistance protein-coding genes, including ABCB1, ABCC1, ABCG2, and ABCF2 (Yahya et al., 2018). Additionally, miR-214, as an oncogenic regulator, impeded the reversal of drug resistance by upregulating ABCC1 and ABCC5, whereas its downregulation led to a significant mitigation in ABCC10 expression (Hamed et al., 2018). In this respect, beyond the specific function, it seems that recruiting miRNAs with multiple APC transporter downstream targets might efficiently overcome doxorubicin chemoresistance and point integral directions for future research.

Emerging shreds of evidence have established that among the signalling pathways, epidermal growth factor receptor (EGFR)/extracellular signal-regulated kinase 1/2 (ERK 1/2) and phosphatase and tensin homolog (PTEN)/PI3K/Akt might be the pioneer ones implicated in miRNA-mediated regulation ABC transporters during doxorubicin drug resistance in HCC (Zhao et al., 2018; Li et al., 2022). EGFR is a tyrosine kinase HER family member extensively expressed on mammalian cell membranes. This receptor is crucial in the development and advancement of various carcinomas, like HCC (He et al., 2023). miR-338-5p has been observed to modulate the MDR of Hep3B and Huh7 cells in an EGFR/ERK1/2 cascade. miR-338-5p has been shown to mitigate the expression of P-gp, thereby enhancing the HCC cell’s sensitivity to doxorubicin and vinblastine, both P-gp substrates. Additionally, miR-338-5p has been observed to restrain HCC cell proliferation by directly modulating the EGFR. In more detail, these conclusions point to miR-338-5p downregulates P-gp through a dual inhibitory mechanism. This involves direct interaction with the 3′-UTR of ABCB1 and suppression of the EGFR-related phosphorylation of the ERK1/2 pathway, ultimately giving rise to increased sensitivity of hepatoma cells to doxorubicin (Zhao et al., 2018). On the other hand, owing to the phosphatase function of PTEN serves a prominent role in regulating the PI3K/Akt signalling pathway; therefore, reduced transcription of PTEN in tumour cells may contribute to unfavourable tumour outcomes and an increased resistance to therapeutic drugs (Alimohammadi et al., 2024). In agreement with this phenomenon, in a recent investigation, miR-205 has been acknowledged as a significant factor in overcoming drug resistance in liver tumour cells. This miRNA enhances therapeutic efficacy by mitigating drug efflux and promoting apoptosis. It achieves this property by upregulating PTEN, abating PI3K/Akt signalling phosphorylation, and subsequent downregulation of P-gp expression. Mechanistically, the transfection of miR-205 into HepG2/DOX in combination with doxorubicin was shown to inhibit multidrug resistance, reduce cell proliferation, and enhance apoptotic cell death (Li et al., 2022).

In conclusion, recent breakthroughs in understanding miRNAs’ role in regulating ABC transporters have presented promising opportunities to overcome doxorubicin resistance in HCC. Specific miRNAs, such as miR-122, miR-338-5p, and miR-205, have shown significant potential in reversing chemoresistance by targeting critical transporters and modulating signalling pathways like EGFR/ERK and PTEN/PI3K/Akt. These miRNAs not only inhibit drug efflux but also enhance cancer cell sensitivity to chemotherapy through mechanisms involving transcriptional repression and post-translational modifications. As future research advances, the integration of miRNA-based therapies with current conventional treatments could hold transformative potential. This approach may lead to more effective, durable responses in HCC patients, minimizing recurrence and metastasis. Additionally, advancements in delivery systems, such as nanoparticle-based miRNA carriers, could further improve the specificity and efficiency of miRNA therapeutics, reducing off-target effects and enhancing drug delivery directly to cancer cells. Investigating the combination of miRNAs targeting multiple ABC transporters with existing immunotherapies or targeted therapies presents another promising avenue. Such combinations could synergistically increase treatment efficacy while mitigating the adaptive resistance mechanisms often observed in HCC. Incorporating these miRNA-based approaches into clinical practice could significantly improve patient outcomes by mitigating resistance and providing more durable responses in HCC treatment. Hence, miRNAs represent not only a vital component in the current fight against HCC but also a promising frontier for the development of more effective cancer therapies.

### 5.11 Renal cancer

Renal cell carcinoma (RCC) is rated among the most fatal urologic cancers globally and is characterized by high morbidity, mortality, and a generally poor prognosis (Hu et al., 2023). Surgical intervention, whether through radical resection or nephron-sparing surgery, remains the primary treatment approach for RCC. However, for patients with end-stage or recurrent RCC, chemotherapy is typically employed, though its efficacy is often limited (Young et al., 2024). The primary challenge with chemotherapy in RCC lies in the development of MDR by RCC cells against agents like vinblastine and doxorubicin. MDR enhances the cancer cell’s ability to evade the cytotoxic effects of chemotherapy, a process tightly modified by ncRNAs, proteins, and various signalling pathways (Aweys et al., 2023; Liu et al., 2023). The lack of response in some RCC patients to standard chemotherapy is likely due to either intrinsic or acquired MDR. Notably, the upregulation of MRP1, P-gp, and BCRP in RCC patients has been observed, where these proteins function as efflux pumps that, aided by ATPase activity, actively expel chemotherapeutic drugs from cancer cells (Li et al., 2018; Long et al., 2015).

As with other types of cancer, miRNAs in RCC play crucial roles in primary biological functions and cancer-related processes (Yu et al., 2024). They exert their effects by attaching to the 3′UTR of target mRNAs, which leads to translation suppression and mRNA degradation, thereby modulating gene expression at the post-transcriptional stage (Okamura et al., 2008). miR-210-3p is among the miRNAs that have been downregulated in the Caki-2/DOX drug-resistant RCC cell line. It has been reported that miR-210-3p refined the doxorubicin drug sensitivity of RCC cells by directly impacting the 3′UTR of ABCC1 and ultimately barricading MRP1 expression (Li et al., 2018). However, miR-124 has adopted more complex mechanisms to influence the transcriptional activity of ABC transporters (Long et al., 2015). Wnt signalling pathways are typically initiated when secreted Wnt ligands bind to the conserved C-terminal cytoplasmic domain of Frizzled (FZD) receptor proteins, a group of multipass transmembrane receptors that includes nine members (Qin et al., 2024). Persistent stimulation of Wnt signalling is crucial in the development of renal cancer. A significant clinical exploration demonstrated that the expression levels of the Wnt ligand receptors Frizzled 5 (FZD5) and FZD8 were significantly elevated in renal carcinoma tissues (Xu et al., 2016). The increased aggressiveness of RCC has been associated with alterations in Wnt1/β-catenin canonical signalling, with β-catenin being linked to poor clinical outcomes and reduced survival rates (Kruck et al., 2013). In this rationale, a well-known study mentioned that Wnt-5a induced phosphorylation of PKC and CamKII in a dose-dependent manner. However, silencing FZD5 or administering a miR-124 mimic attenuated the impact of Wnt-5a on PKC. These observations suggest that the Wnt-5a-FZD5 signaling pathway may contribute to doxorubicin resistance by promoting P-gp upregulation through PKCα-mediated phosphorylation. Simultaneously it has been revealed that in doxorubicin-resistant renal cancer cells, miR-124 expression was notably reduced, correlating with elevated levels of FZD5 and P-gp. Restoration of miR-124 in Caki-2/DOX cells significantly improved drug sensitivity and induced increased cell apoptosis. Additionally, miR-124 was found to target FZD5, leading to its downregulation, which subsequently resulted in the deactivation of PKC and a reduction in P-gp expression. These shreds of evidence point to the restoration of miR-124, which could serve as an upcoming alternative therapeutic approach for combating doxorubicin resistance in advanced or metastatic renal cell carcinoma (Long et al., 2015).

Current understanding indicates that ncRNAs, particularly miRNAs like miR-124 and miR-210-3p, significantly modulate the expression of efflux transporters such as MRP1 and P-gp, contributing to the chemoresistance observed in RCC. Future research should focus on elucidating the specific regulatory networks involving these miRNAs and their potential as therapeutic agents. For instance, the restoration of miR-124 presents a promising avenue for overcoming doxorubicin resistance, but comprehensive studies are essential to explore the broader implications of miRNA therapy in clinical settings. Additionally, targeting associated signalling pathways, such as the Wnt/β-catenin pathway, may offer synergistic effects when combined with traditional chemotherapeutic approaches.

### 5.12 Neuroblastoma

Neuroblastoma accounts for 15% of all childhood cancer-attributed deaths and initially shows a favourable response to chemotherapy (Valter et al., 2018). However, it often recurs in a form that is resistant to treatment. Research indicates that this chemoresistance may be associated with enhanced drug efflux due to the upregulation of ABC transporters, such as MDR1, coupled with the epigenetic silencing of cancer inhibitor genes via DNA hypermethylation (Veschi et al., 2024). The constitutive androstane receptor (CAR) acts as a critical xenosensor in regulating MDR. It plays a vital role in foreign substance detoxification by controlling the transcription of phase I drug-metabolizing protein regulators and ABC transporters. The overexpression of these transporters in cancer cells and their involvement in drug resistance positions them as promising pharmacological targets for mitigating MDR, specifically in neuroblastoma (Stern et al., 2022; Wang et al., 2010). In this rationale, the research conducted by Takwi et al. established that the miR-137 promoter hypermethylation, along with its negative regulation by CAR, partially accounts for the mitigated transcriptional level of miR-137 and the elevated levels of CAR and P-gp in doxorubicin-resistant neuroblastoma cells. These results highlight the critical role of miR-137 in modifying the cancer cells’ reaction to doxorubicin treatment and underscore miR-137 as an extremely favourable target for reducing CAR-related P-gp protein levels in doxorubicin-resistant neuroblastoma tumour cells (Takwi et al., 2014).

## 6 miRNAs targeting ABC transporters: from clinical trials aspects

To address ABC transporter-mediated MDR, researchers have increasingly focused on ncRNA-based therapeutic approaches. These strategies aim to correct the dysregulation of ncRNAs, which play a crucial role in drug-resistant cancer cells. One promising avenue involves the application of CRISPR/Cas9 technology to modify ncRNAs, potentially inhibiting cancer growth and regulating the expression of MDR-related ncRNAs (Yang et al., 2023). By doing so, this approach could reduce drug efflux, which is often mediated by ABC transporters, thereby overcoming drug resistance mechanisms in cancer cells. The potential of miRNA-based therapeutics has been explored in preclinical and clinical settings. One approach involves the use of miRNA mimics or inhibitors to correct the dysregulation of miRNAs that contribute to MDR. For example, miR-15b has been identified as a key regulator in reversing doxorubicin resistance in osteosarcoma models by targeting the ABC transporters and other associated resistance pathways. In a mouse xenograft model, miR-15b downregulation was observed in resistant osteosarcoma cell lines, and its reconstitution significantly increased sensitivity to doxorubicin, reducing tumour growth. Clinically, low levels of miR-15b were associated with poor patient outcomes, further highlighting its potential as a biomarker for treatment prognosis (Duan et al., 2017).

Despite the potential, a major hurdle in designing ncRNA-based therapies to combat MDR is the associated toxicity. A pertinent example is the development of valspodar, a small molecule inhibitor targeting ABCB1, which was designed to re-sensitize MDR tumours to chemotherapeutic agents. However, this approach faced significant setbacks. In a Phase III clinical trial (CALGB 9720), the combination of valspodar with chemotherapy had to be halted prematurely due to high mortality rates observed in the experimental group (Baer et al., 2002). This failure was linked to the off-target effects of valspodar, notably its inhibition of CYP3A4, a cytochrome P450 enzyme critical for drug metabolism (Sparreboom and Nooter, 2000). The concurrent inhibition of ABCB1 and CYP3A4 led to increased drug toxicity in normal tissues, highlighting the need for specificity in modulating ABC transporters in future therapeutic strategies. Another example of the complexities involved in ncRNA-based therapies is the Phase I clinical trial of MRX34, a liposomal miR-34a mimic, which was being tested for its efficacy in cancer treatment (NCT01829971). Despite its initial promise, the trial was terminated due to severe immune-related toxicities, including patient deaths (Hong et al., 2020). This outcome further underlines the delicate balance between therapeutic efficacy and safety when manipulating ncRNAs for clinical purposes. While miRNAs like miR-34a have shown great potential in regulating ABC transporter expression and reversing chemoresistance, the challenge remains in developing delivery methods and therapeutic combinations that minimize adverse effects. While significant progress has been made in understanding the role of miRNAs in drug resistance, especially through their regulation of ABC transporters, it is important to note that no clinical trials have yet been conducted specifically to investigate miRNAs’ direct impact on ABC transporters in overcoming drug resistance.

Several challenges have hindered the advancement of miRNA-based therapies in clinical settings. Among these are toxicity-related issues, as seen in trials like MRX34, and the off-target effects observed in ABC transporter modulators such as Valspodar (Baer et al., 2002; Hong et al., 2020). Furthermore, the delivery of miRNA therapies remains a major obstacle, as efficient, targeted delivery to cancer cells without affecting normal tissues is essential to avoid adverse effects (Seyhan, 2024). Additionally, the complexity of miRNA networks and their pleiotropic effects pose a significant challenge. miRNAs often regulate multiple genes and pathways simultaneously, making it difficult to achieve selective modulation of ABC transporters without unintended consequences (To et al., 2024). The lack of specificity in current miRNA delivery methods, combined with the potential for immune-related toxicities, further complicates their clinical translation (Chakraborty et al., 2021). For these reasons, while preclinical research continues to demonstrate miRNAs’ potential to modulate ABC transporters and reverse drug resistance, further refinement in the therapeutic design, targeting, and safety profiles is needed before clinical trials in this area can be initiated.

Thus, while preclinical miRNA-based therapeutics and other interventions offer innovative strategies to target and modulate ABC transporter-related drug resistance, the specificity and safety of these approaches must be carefully evaluated. The lessons learned from the valspodar and MRX34 trials emphasize the importance of ensuring that any modulation of ABC transporters in cancer therapy does not inadvertently increase toxicity to normal tissues. Moving forward, a greater focus on optimizing delivery systems and refining the targets of miRNA modulation will be crucial in translating these findings into safe and effective clinical treatments.

## 7 Conclusion

Doxorubicin remains a widely used first-line chemotherapeutic agent in the treatment of various cancers, but the development of resistance significantly limits its long-term efficacy. Understanding the molecular mechanisms responsible for doxorubicin resistance, particularly the role of ABC transporters, is crucial for developing effective strategies to overcome this challenge. miRNAs, as key regulators of gene expression, have emerged as potent modulators of ABC transporters, reversing resistance by targeting specific members like ABCB1, ABCC1, and ABCG2, among others.

The potential of miRNAs to enhance cancer treatment by targeting these transporters presents a promising therapeutic avenue. However, the clinical translation of miRNA-based therapies faces significant obstacles. One major challenge is the lack of efficient delivery systems that can protect miRNAs from degradation in the bloodstream and ensure their specific delivery to target cells without affecting healthy tissues. Innovative approaches, such as nanoparticle-based or viral vector delivery systems, are being explored but require further development to ensure safety, stability, and precision.

**FIGURE 5:**
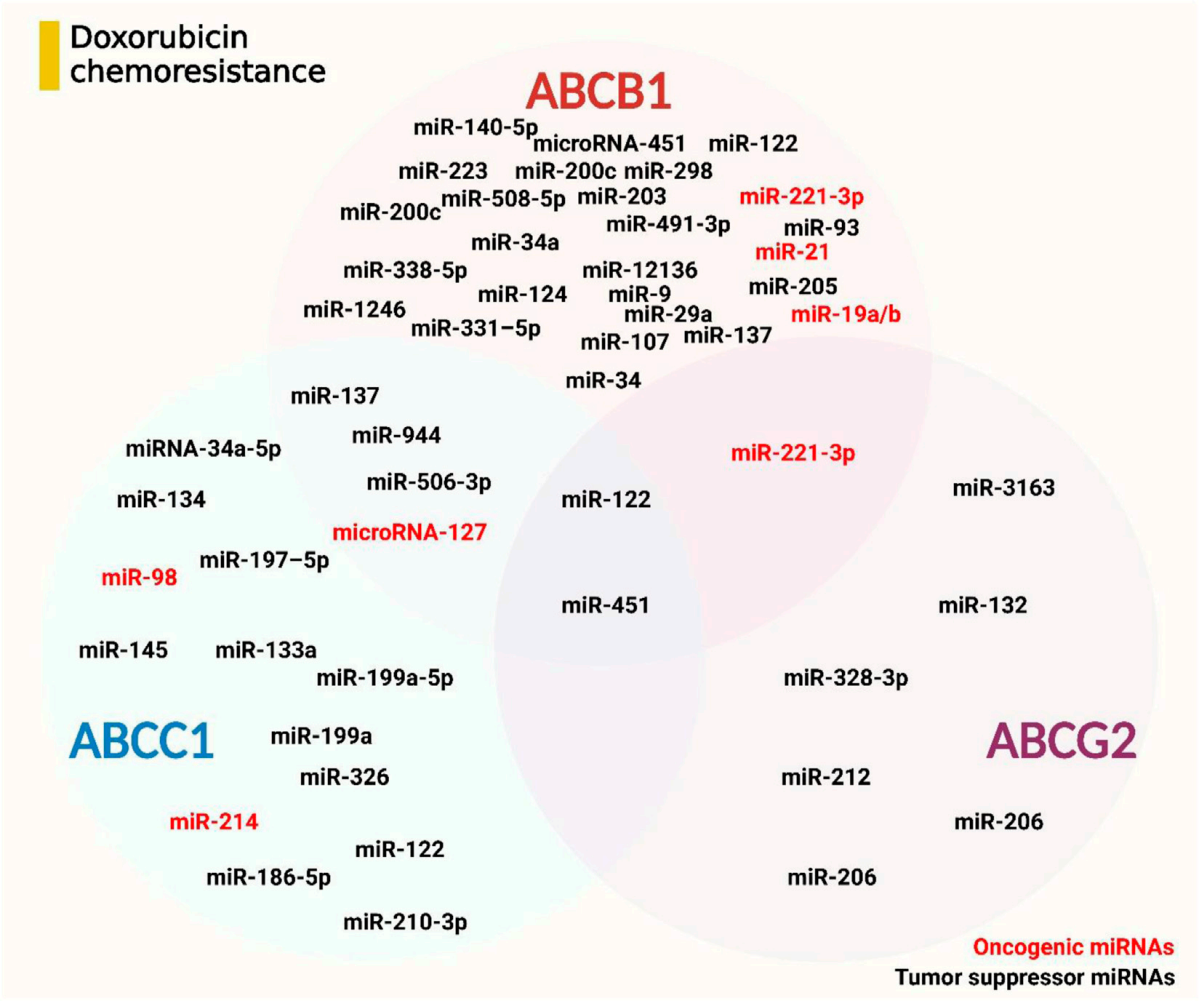
MiRNAs involved in regulating ABC transporters contribute to doxorubicin resistance in cancer cells. The Venn diagram illustrates the roles of specific miRNAs in modulating the expression of three key ATP-binding cassette ABC transporters: ABCB1, ABCC1, and ABCG2, all of which are implicated in mediating resistance to doxorubicin by promoting its efflux from cancer cells. Tumour suppressor miRNAs (black text) and oncogenic miRNAs (red text) are mapped to each transporter based on their roles in either inhibiting or promoting chemoresistance. ABCB1 is regulated by a broad spectrum of miRNAs, including tumour suppressors like miR-34a, miR-124, and miR-338-5p, which can potentially reverse resistance, and oncogenic miRNAs such as miR-221-3p, miR-21, and miR-19a/b, which enhance resistance by upregulating ABCB1 expression. ABCC1 is associated with miRNAs like miR-145 and miR-186-5p, which act as tumour suppressors, while miR-214 promotes chemoresistance by upregulating ABCC1 expression. ABCG2 is modulated by miRNAs such as miR-328-3p and miR-132, which regulate its expression, with miR-221-3p playing a dual role across both ABCB1 and ABCG2 transporters. The overlap of miRNAs between the transporters highlights shared regulatory mechanisms, suggesting that some miRNAs like miR-122 and miR-451 target multiple transporters, thereby playing a broader role in multidrug resistance. Understanding these miRNA-transporter interactions can provide insights into the development of therapeutic strategies aimed at overcoming doxorubicin resistance in cancer cells.

Figure 5 illustrates the complex network of miRNAs that modulate the expression of ABCB1, ABCC1, and ABCG2, highlighting both tumor-suppressing and oncogenic miRNAs. The overlap of miRNA regulation across multiple transporters underscores the redundancy and complexity of the mechanisms driving multidrug resistance. The pleiotropic nature of miRNAs, which can regulate multiple genes, poses a risk of off-target effects. This complexity underscores the importance of designing highly specific miRNA therapeutics that minimize unintended consequences on non-target pathways. Another limitation is the tumour heterogeneity observed among different patients and even within the same tumour, where varying levels of miRNA expression and ABC transporter activity may influence the efficacy of miRNA-based treatments. Personalized approaches that consider the molecular profile of individual tumours will be essential for improving therapeutic outcomes.

Future research should prioritize the refinement of these delivery vehicles, focusing on improving their stability, biocompatibility, and specificity. Nanoparticles, for instance, offer a promising avenue due to their ability to encapsulate miRNAs and deliver them directly to tumour tissues. Research into optimizing these systems to bypass biological barriers, such as the immune system or the tumour microenvironment, is crucial for enhancing the efficacy of miRNA-based treatments. Focus on identifying biomarkers that predict miRNA responsiveness in specific patient populations, enabling the development of personalized treatment strategies. Studies investigating the molecular profiles of tumours and their correlation with miRNA expression and transporter activity could help design tailored therapeutic approaches that maximize effectiveness. Enhancing the specificity of miRNA-based treatments, ensuring they selectively target ABC transporters involved in drug resistance without affecting other critical pathways. Techniques such as CRISPR-based genome editing and improved miRNA mimics may help enhance precision by limiting off-target gene regulation, thus reducing the risk of adverse effects. Exploring the potential of combining miRNA-based therapies with conventional chemotherapy or other targeted treatments. By integrating miRNA therapeutics with established cancer treatments, it may be possible to enhance the cytotoxic effects of drugs like doxorubicin while simultaneously overcoming resistance mechanisms. For instance, combining miRNA-based therapy with inhibitors of other resistance pathways, such as PI3K/Akt or EGFR signalling, could result in synergistic effects that improve treatment outcomes. Preclinical and clinical trials investigating the safety and efficacy of such combination strategies are needed to determine their therapeutic potential. Even more, conducting well-designed clinical trials to assess the safety, efficacy, and long-term effects of miRNA therapies in cancer patients. Understanding the potential for immune responses, toxicity, and the durability of treatment effects will be essential for bringing miRNA-based therapies to clinical practice. Additionally, investigating the pharmacokinetics and pharmacodynamics of miRNA delivery systems will provide crucial insights into their behaviour *in vivo* and inform the development of next-generation therapeutics.

In conclusion, miRNAs offer a promising therapeutic avenue for overcoming doxorubicin resistance by targeting ABC transporters. By continuing to advance research in this field and addressing these challenges, miRNA-based strategies may become a transformative tool in cancer treatment, offering new hope for patients struggling with drug-resistant tumours.

## References

[B1] Aas T. Børresen A.-L. Geisler S. Smith-Sørensen B. Johnsen H. Varhaug J. E. (1996). Specific P53 mutations are associated with *de novo* resistance to doxorubicin in breast cancer patients. Nat. Med. 2 (7), 811–814. 10.1038/nm0796-811 8673929

[B2] Achkar N. P. Cambiagno D. A. Manavella P. A. (2016). miRNA biogenesis: a dynamic pathway. Trends Plant Sci. 21 (12), 1034–1044. 10.1016/j.tplants.2016.09.003 27793495

[B3] Adams C. Cazzanelli G. Rasul S. Hitchinson B. Hu Y. Coombes R. C. (2015). Apoptosis inhibitor TRIAP1 is a novel effector of drug resistance. Oncol. Rep. 34 (1), 415–422. 10.3892/or.2015.3988 25998939

[B4] Alam A. Locher K. P. (2023). Structure and mechanism of human ABC transporters. Annu. Rev. Biophysics 52, 275–300. 10.1146/annurev-biophys-111622-091232 36737602

[B5] Aldossary S. A. (2019). Review on pharmacology of cisplatin: clinical use, toxicity and mechanism of resistance of cisplatin. Biomed. Pharmacol. J. 12 (1), 07–15. 10.13005/bpj/1608

[B6] Alimohammadi M. Rahimzadeh P. Khorrami R. Bonyadi M. Daneshi S. Nabavi N. (2024). A comprehensive review of the PTEN/PI3K/Akt axis in multiple myeloma: from molecular interactions to potential therapeutic targets. Pathology - Res. Pract. 260, 155401. 10.1016/j.prp.2024.155401 38936094

[B7] Almohammad Aljabr B. Zihlif M. Abu-Dahab R. Zalloum H. (2024). Effect of quercetin on doxorubicin cytotoxicity in sensitive and resistant human MCF7 breast cancer cell lines. Biomed. Rep. 20 (4), 58. 10.3892/br.2024.1745 38414625 PMC10895388

[B8] Al-Momany B. Hammad H. Ahram M. (2021). Dihydrotestosterone induces chemo-resistance of triple-negative breast MDA-MB-231 cancer cells towards doxorubicin independent of ABCG2 and miR-328-3p. Curr. Mol. Pharmacol. 14 (5), 860–870. 10.2174/1874467214666210531170355 34061013

[B9] Anastasiadou E. Jacob L. S. Slack F. J. (2018). Non-coding RNA networks in cancer. Nat. Rev. Cancer 18 (1), 5–18. 10.1038/nrc.2017.99 29170536 PMC6337726

[B10] Armada A. Gomes B. C. Viveiros M. Rueff J. Rodrigues A. S. (2019). Regulation of ABCB1 activity by microRNA-200c and microRNA-203a in breast cancer cells: the quest for microRNAs’ involvement in cancer drug resistance. Cancer Drug Resist. 2 (3), 897–911. 10.20517/cdr.2019.24 35582584 PMC8992503

[B11] Armstrong J. Dass C. R. (2018). Doxorubicin action on mitochondria: relevance to osteosarcoma therapy? Curr. drug targets 19 (5), 432–438. 10.2174/1389450116666150416115852 25882220

[B12] Ashique S. Bhowmick M. Pal R. Khatoon H. Kumar P. Sharma H. (2024). Multi drug resistance in Colorectal Cancer-approaches to overcome, advancements and future success. Adv. Cancer Biol. - Metastasis 10, 100114. 10.1016/j.adcanc.2024.100114

[B13] Ashrafizadeh M. Dai J. Torabian P. Nabavi N. Aref A. R. Aljabali A. A. A. (2024). Circular RNAs in EMT-driven metastasis regulation: modulation of cancer cell plasticity, tumorigenesis and therapy resistance. Cell Mol. Life Sci. 81 (1), 214. 10.1007/s00018-024-05236-w 38733529 PMC11088560

[B14] Aweys H. Lewis D. Sheriff M. Rabbani R. D. Lapitan P. Sanchez E. (2023). Renal cell cancer – insights in drug resistance mechanisms. Anticancer Res. 43 (11), 4781–4792. 10.21873/anticanres.16675 37909991

[B15] Baer M. R. George S. L. Dodge R. K. O'Loughlin K. L. Minderman H. Caligiuri M. A. (2002). Phase 3 study of the multidrug resistance modulator PSC-833 in previously untreated patients 60 years of age and older with acute myeloid leukemia: cancer and Leukemia Group B Study 9720. Blood 100 (4), 1224–1232. 10.1182/blood.v100.4.1224.h81602001224_1224_1232 12149202

[B16] Banerjee A. Pata J. Chaptal V. Boumendjel A. Falson P. Prasad R. (2023). Structure, function, and inhibition of catalytically asymmetric ABC transporters: lessons from the PDR subfamily. Drug Resist. Updat. 71, 100992. 10.1016/j.drup.2023.100992 37567064

[B17] Bao L. Hazari S. Mehra S. Kaushal D. Moroz K. Dash S. (2012). Increased expression of P-glycoprotein and doxorubicin chemoresistance of metastatic breast cancer is regulated by miR-298. Am. J. Pathology 180 (6), 2490–2503. 10.1016/j.ajpath.2012.02.024 PMC3378910 22521303

[B18] Blukacz L. Nuciforo S. Fucile G. Trulsson F. Duthaler U. Wieland S. (2024). Inhibition of the transmembrane transporter ABCB1 overcomes resistance to doxorubicin in patient-derived organoid models of HCC. Hepatol. Commun. 8 (5), e0437. 10.1097/HC9.0000000000000437 38696353 PMC11068137

[B19] Bogman K. Peyer A. K. Török M. Küsters E. Drewe J. (2001). HMG-CoA reductase inhibitors and P-glycoprotein modulation. Br. J. Pharmacol. 132 (6), 1183–1192. 10.1038/sj.bjp.0703920 11250868 PMC1572659

[B20] Bourguignon L. Y. Spevak C. C. Wong G. Xia W. Gilad E. (2009). Hyaluronan-CD44 interaction with protein kinase C(epsilon) promotes oncogenic signaling by the stem cell marker Nanog and the Production of microRNA-21, leading to down-regulation of the tumor suppressor protein PDCD4, anti-apoptosis, and chemotherapy resistance in breast tumor cells. J. Biol. Chem. 284 (39), 26533–26546. 10.1074/jbc.M109.027466 19633292 PMC2785342

[B21] Bray F. Laversanne M. Sung H. Ferlay J. Siegel R. L. Soerjomataram I. (2024). Global cancer statistics 2022: GLOBOCAN estimates of incidence and mortality worldwide for 36 cancers in 185 countries. CA A Cancer J. Clin. 74 (3), 229–263. 10.3322/caac.21834 38572751

[B22] Cai Y. Yu X. Hu S. Yu J. (2009). A brief review on the mechanisms of miRNA regulation. Genomics, proteomics and Bioinforma. 7 (4), 147–154. 10.1016/S1672-0229(08)60044-3 PMC5054406 20172487

[B23] Cao X. Hou J. An Q. Assaraf Y. G. Wang X. (2020a). Towards the overcoming of anticancer drug resistance mediated by p53 mutations. Drug Resist. Updat. 49, 100671. 10.1016/j.drup.2019.100671 31841768

[B24] Cao Y. X. Wen F. Luo Z. Y. Long X. X. Luo C. Liao P. (2020b). Downregulation of microRNA let-7f mediated the Adriamycin resistance in leukemia cell line. J. Cell Biochem. 121 (10), 4022–4033. 10.1002/jcb.29541 31793054

[B25] Carabias P. Espelt M. V. Bacigalupo M. L. Rojas P. Sarrias L. Rubin A. (2022). Galectin-1 confers resistance to doxorubicin in hepatocellular carcinoma cells through modulation of P-glycoprotein expression. Cell Death and Dis. 13 (1), 79. 10.1038/s41419-022-04520-6 PMC8786848 35075112

[B26] Chakraborty C. Sharma A. R. Sharma G. Lee S.-S. (2021). Therapeutic advances of miRNAs: a preclinical and clinical update. J. Adv. Res. 28, 127–138. 10.1016/j.jare.2020.08.012 33364050 PMC7753224

[B27] Chan K.-T. Lung M. L. (2004). Mutant p53 expression enhances drug resistance in a hepatocellular carcinoma cell line. Cancer Chemother. Pharmacol. 53, 519–526. 10.1007/s00280-004-0767-4 15004724

[B28] Chang L. Hu Z. Zhou Z. Zhang H. (2018). Linc00518 contributes to multidrug resistance through regulating the MiR-199a/MRP1 axis in breast cancer. Cell. Physiology Biochem. 48 (1), 16–28. 10.1159/000491659 30001527

[B29] Chawra H. S. Agarwal M. Mishra A. Chandel S. S. Singh R. P. Dubey G. (2024). MicroRNA-21's role in PTEN suppression and PI3K/AKT activation: implications for cancer biology. Pathology-Research Pract. 254, 155091. 10.1016/j.prp.2024.155091 38194804

[B30] Chen H.-K. Chen Y.-L. Wang C.-Y. Chung W.-P. Fang J.-H. Lai M.-D. (2023b). ABCB1 regulates immune genes in breast cancer. Breast Cancer Targets Ther. 15 (null), 801–811. 10.2147/BCTT.S421213 PMC10655737 38020048

[B31] Chen J. Tian W. Cai H. He H. Deng Y. (2012). Down-regulation of microRNA-200c is associated with drug resistance in human breast cancer. Med. Oncol. 29, 2527–2534. 10.1007/s12032-011-0117-4 22101791

[B32] Chen L. He Y. Zhu J. Zhao S. Qi S. Chen X. (2023a). The roles and mechanism of m(6)A RNA methylation regulators in cancer immunity. Biomed. Pharmacother. 163, 114839. 10.1016/j.biopha.2023.114839 37156113

[B33] Chen M. Li D. Gong N. Wu H. Su C. Xie C. (2017). miR-133b down-regulates ABCC1 and enhances the sensitivity of CRC to anti-tumor drugs. Oncotarget 8 (32), 52983–52994. 10.18632/oncotarget.17677 28881788 PMC5581087

[B34] Chen Y. Feng X. Yuan Y. Jiang J. Zhang P. Zhang B. (2022a). Identification of a novel mechanism for reversal of doxorubicin-induced chemotherapy resistance by TXNIP in triple-negative breast cancer via promoting reactive oxygen-mediated DNA damage. Cell Death and Dis. 13 (4), 338. 10.1038/s41419-022-04783-z PMC9005717 35414060

[B35] Chen Y. Li X. Shi L. Ma P. Wang W. Wu N. (2022b). Combination of 7-O-geranylquercetin and microRNA-451 enhances antitumor effect of Adriamycin by reserving P-gp-mediated drug resistance in breast cancer. Aging (Albany NY) 14 (17), 7156–7169. 10.18632/aging.204287 36107024 PMC9512499

[B36] Chen Y.-L. Yang T.-Y. Chen K.-C. Wu C.-L. Hsu S.-L. Hsueh C.-M. (2016). Hypoxia can impair doxorubicin resistance of non-small cell lung cancer cells by inhibiting MRP1 and P-gp expression and boosting the chemosensitizing effects of MRP1 and P-gp blockers. Cell. Oncol. 39 (5), 411–433. 10.1007/s13402-016-0285-5 PMC13001888 27306525

[B37] Chen Z. Pan T. Jiang D. Jin L. Geng Y. Feng X. (2020). The lncRNA-GAS5/miR-221-3p/DKK2 Axis modulates ABCB1-mediated adriamycin resistance of breast cancer via the wnt/β-catenin signaling pathway. Mol. Therapy-Nucleic Acids 19, 1434–1448. 10.1016/j.omtn.2020.01.030 PMC7056627 32160712

[B38] Choi B. H. Ryu D. Y. Ryoo I. G. Kwak M. K. (2017). NFE2L2/NRF2 silencing-inducible miR-206 targets c-MET/EGFR and suppresses BCRP/ABCG2 in cancer cells. Oncotarget 8 (63), 107188–107205. 10.18632/oncotarget.22513 29291022 PMC5739807

[B39] Christidi E. Brunham L. R. (2021). Regulated cell death pathways in doxorubicin-induced cardiotoxicity. Cell Death and Dis. 12 (4), 339. 10.1038/s41419-021-03614-x PMC8017015 33795647

[B40] Cox J. Weinman S. (2016). Mechanisms of doxorubicin resistance in hepatocellular carcinoma. Hepatic Oncol. 3 (1), 57–59. 10.2217/hep.15.41 PMC4792121 26998221

[B41] Dai X. Wang L. Deivasigamni A. Looi C. Y. Karthikeyan C. Trivedi P. (2017). A novel benzimidazole derivative, MBIC inhibits tumor growth and promotes apoptosis via activation of ROS-dependent JNK signaling pathway in hepatocellular carcinoma. Oncotarget 8 (8), 12831–12842. 10.18632/oncotarget.14606 28086233 PMC5355059

[B42] Das M. Padda S. K. Weiss J. Owonikoko T. K. (2021). Advances in treatment of recurrent small cell lung cancer (SCLC): insights for optimizing patient outcomes from an expert roundtable discussion. Adv. Ther. 38 (11), 5431–5451. 10.1007/s12325-021-01909-1 34564806 PMC8475485

[B43] Dean M. Hamon Y. Chimini G. (2001). The human ATP-binding cassette (ABC) transporter superfamily. J. lipid Res. 42 (7), 1007–1017. 10.1016/s0022-2275(20)31588-1 11441126

[B44] Du J. Liu S. He J. Liu X. Qu Y. Yan W. (2015). MicroRNA-451 regulates stemness of side population cells via PI3K/Akt/mTOR signaling pathway in multiple myeloma. Oncotarget 6 (17), 14993–15007. 10.18632/oncotarget.3802 25915427 PMC4558131

[B45] Duan C. Yu M. Xu J. Li B.-Y. Zhao Y. Kankala R. K. (2023). Overcoming cancer multi-drug resistance (MDR): reasons, mechanisms, nanotherapeutic solutions, and challenges. Biomed. and Pharmacother. 162, 114643. 10.1016/j.biopha.2023.114643 37031496

[B46] Duan Z. Gao Y. Shen J. Choy E. Cote G. Harmon D. (2017). miR-15b modulates multidrug resistance in human osteosarcoma *in vitro* and *in vivo* . Mol. Oncol. 11 (2), 151–166. 10.1002/1878-0261.12015 28145098 PMC5300234

[B47] Ebrahimnezhad M. Asl S. H. Rezaie M. Molavand M. Yousefi B. Majidinia M. (2024). lncRNAs: new players of cancer drug resistance via targeting ABC transporters. IUBMB Life. 76, 883–921. 10.1002/iub.2888 39091106

[B48] Elfadadny A. Ragab R. F. Hamada R. Al Jaouni S. K. Fu J. Mousa S. A. (2023). Natural bioactive compounds-doxorubicin combinations targeting topoisomerase II-alpha: anticancer efficacy and safety. Toxicol. Appl. Pharmacol. 461, 116405. 10.1016/j.taap.2023.116405 36716865

[B49] Feng D. D. Zhang H. Zhang P. Zheng Y. S. Zhang X. J. Han B. W. (2011). Down-regulated miR-331-5p and miR-27a are associated with chemotherapy resistance and relapse in leukaemia. J. Cell Mol. Med. 15 (10), 2164–2175. 10.1111/j.1582-4934.2010.01213.x 21070600 PMC4394226

[B50] Feng F. Wu J. Chi Q. Wang S. Liu W. Yang L. (2024). Lactylome analysis unveils lactylation-dependent mechanisms of stemness remodeling in the liver cancer stem cells. Adv. Sci. (Weinh) 11, e2405975. 10.1002/advs.202405975 39099416 PMC11481176

[B51] Feng R. Dong L. (2015). Knockdown of microRNA-127 reverses adriamycin resistance via cell cycle arrest and apoptosis sensitization in adriamycin-resistant human glioma cells. Int. J. Clin. Exp. Pathology 8 (6), 6107–6116. PMC4525822 26261488

[B52] Gabbia D. De Martin S. (2024). Insights into hepatocellular carcinoma: from pathophysiology to novel therapies. Int. J. Mol. Sci. 25 (8), 4188. 10.3390/ijms25084188 38673774 PMC11049888

[B53] Gao M. Miao L. Liu M. Li C. Yu C. Yan H. (2016). miR-145 sensitizes breast cancer to doxorubicin by targeting multidrug resistance-associated protein-1. Oncotarget 7 (37), 59714–59726. 10.18632/oncotarget.10845 27487127 PMC5312343

[B54] Garrido-Cano I. Adam-Artigues A. Lameirinhas A. Blandez J. F. Candela-Noguera V. Rojo F. (2022). miR-99a-5p modulates doxorubicin resistance via the COX-2/ABCG2 axis in triple-negative breast cancer: from the discovery to *in vivo* studies. Cancer Commun. 42 (12), 1412–1416. 10.1002/cac2.12352 PMC9759767 35997029

[B55] Go H. Jang J. Y. Kim P. J. Kim Y. G. Nam S. J. Paik J. H. (2015). MicroRNA-21 plays an oncogenic role by targeting FOXO1 and activating the PI3K/AKT pathway in diffuse large B-cell lymphoma. Oncotarget 6 (17), 15035–15049. 10.18632/oncotarget.3729 25909227 PMC4558134

[B56] Gomes B. C. Honrado M. Armada A. Viveiros M. Rueff J. Rodrigues A. S. (2020). ABC efflux transporters and the circuitry of miRNAs: kinetics of expression in cancer drug resistance. Int. J. Mol. Sci. 21 (8), 2985. 10.3390/ijms21082985 32340269 PMC7215654

[B57] Goossens J.-F. Bailly C. (2019). Ursodeoxycholic acid and cancer: from chemoprevention to chemotherapy. Pharmacol. and Ther. 203, 107396. 10.1016/j.pharmthera.2019.107396 31356908

[B58] Guo Q. Grimmig T. Gonzalez G. Giobbie-Hurder A. Berg G. Carr N. (2018). ATP-binding cassette member B5 (ABCB5) promotes tumor cell invasiveness in human colorectal cancer. J. Biol. Chem. 293 (28), 11166–11178. 10.1074/jbc.RA118.003187 29789423 PMC6052213

[B59] Guo Y. Tang Y. Lu G. Gu J. (2023). p53 at the crossroads between doxorubicin-induced cardiotoxicity and resistance: a nutritional balancing act. Nutrients 15 (10), 2259. 10.3390/nu15102259 37242146 PMC10222243

[B60] Ha M. Kim V. N. (2014). Regulation of microRNA biogenesis. Nat. Rev. Mol. Cell Biol. 15 (8), 509–524. 10.1038/nrm3838 25027649

[B61] Hafezi N. Valadan R. Asgarian O. H. Ajami A. (2022). Anti-tumor activity of a recombinant soluble Fzd7 decoy receptor in human gastric and colon cancer cells. Iran. J. Basic Med. Sci. 25 (2), 187–192. 10.22038/IJBMS.2022.61908.13700 35655594 PMC9124529

[B62] Halim V. A. García-Santisteban I. Warmerdam D. O. van den Broek B. Heck A. J. R. Mohammed S. (2018). Doxorubicin-induced DNA damage causes extensive ubiquitination of ribosomal proteins associated with a decrease in protein translation. Mol. and Cell. Proteomics 17 (12), 2297–2308. 10.1074/mcp.RA118.000652 PMC6283304 29438997

[B63] Hamed A. R. Emara M. Soltan M. M. Yahya S. M. M. Nabih H. K. Elsayed G. H. (2018). Investigating the role of miRNA-98 and miRNA-214 in chemoresistance of HepG2/Dox cells: studying their effects on predicted ABC transporters targets. Med. Chem. Res. 27 (2), 531–537. 10.1007/s00044-017-2079-3

[B64] Hamilton G. Hochmair M. J. Stickler S. (2024). Overcoming resistance in small-cell lung cancer. Expert Rev. Respir. Med. 18, 569–580. 10.1080/17476348.2024.2388288 39099310

[B65] Hanssen K. M. Haber M. Fletcher J. I. (2021). Targeting multidrug resistance-associated protein 1 (MRP1)-expressing cancers: beyond pharmacological inhibition. Drug Resist. Updat. 59, 100795. 10.1016/j.drup.2021.100795 34983733

[B66] Hauptman N. Glavač D. (2013). Long non-coding RNA in cancer. Int. J. Mol. Sci. 14 (3), 4655–4669. 10.3390/ijms14034655 23443164 PMC3634483

[B67] Hayes J. Peruzzi P. P. Lawler S. (2014). MicroRNAs in cancer: biomarkers, functions and therapy. Trends Mol. Med. 20 (8), 460–469. 10.1016/j.molmed.2014.06.005 25027972

[B68] Hays E. Bonavida B. (2019). YY1 regulates cancer cell immune resistance by modulating PD-L1 expression. Drug Resist. Updat. 43, 10–28. 10.1016/j.drup.2019.04.001 31005030

[B69] He Q. Qu M. Bao H. Xu Y. Shen T. Tan D. (2023). Multiple post-translational modifications ensure EGFR functionality: potential therapeutic targets to overcome its drug-resistance mutations. Cytokine Growth Factor Rev. 70, 41–53. 10.1016/j.cytogfr.2023.03.003 36934069

[B70] He X. Jiang Z. Akakuru O. U. Li J. Wu A. (2021). Nanoscale covalent organic frameworks: from controlled synthesis to cancer therapy. Chem. Commun. (Camb) 57 (93), 12417–12435. 10.1039/d1cc04846e 34734601

[B71] Hong D. S. Kang Y. K. Borad M. Sachdev J. Ejadi S. Lim H. Y. (2020). Phase 1 study of MRX34, a liposomal miR-34a mimic, in patients with advanced solid tumours. Br. J. Cancer 122 (11), 1630–1637. 10.1038/s41416-020-0802-1 32238921 PMC7251107

[B72] Hong L. Piao Y. Han Y. Wang J. Zhang X. Du Y. (2005). Zinc ribbon domain-containing 1 (ZNRD1) mediates multidrug resistance of leukemia cells through regulation of P-glycoprotein and Bcl-2. Mol. Cancer Ther. 4 (12), 1936–1942. 10.1158/1535-7163.MCT-05-0182 16373708

[B73] Hosseini S. A. Mirzaei S. A. Kermani S. Yaghoobi H. (2024). Valproate modulates the activity of multidrug resistance efflux pumps, as a chemoresistance factor in gastric cancer cells. Mol. Biol. Rep. 51 (1), 427. 10.1007/s11033-024-09284-0 38498238

[B74] Hu D. Li M. Su J. Miao K. Qiu X. (2019). Dual-targeting of miR-124-3p and ABCC4 promotes sensitivity to adriamycin in breast cancer cells. Genet. Test. Mol. biomarkers 23 (3), 156–165. 10.1089/gtmb.2018.0259 30807260

[B75] Hu M. Fan J. Y. Zhou X. Cao G. W. Tan X. (2023). Global incidence and mortality of renal cell carcinoma in 2020. Zhonghua Liu Xing Bing Xue Za Zhi 44 (4), 575–580. 10.3760/cma.j.cn112338-20220624-00558 37147828

[B76] Huang J. Ecker G. F. (2023). A structure-based view on ABC-transporter linked to multidrug resistance. Molecules 28 (2), 495. 10.3390/molecules28020495 36677553 PMC9862083

[B77] Huang W. Zhang J. Dong B. Chen H. Shao L. Li X. (2021). A novel miR-98 negatively regulates the resistance of endometrial cancer cells to paclitaxel by suppressing ABCC10/MRP-7. Front. Oncol. 11, 809410. 10.3389/fonc.2021.809410 34950596 PMC8688247

[B78] Iwu C. D. Iwu-Jaja C. J. (2023). Gastric cancer epidemiology: current trend and future direction. Hygiene 3 (3), 256–268. 10.3390/hygiene3030019

[B79] Jain N. Das B. Mallick B. (2022). miR-197-5p increases Doxorubicin-mediated anticancer cytotoxicity of HT1080 fibrosarcoma cells by decreasing drug efflux. DNA Repair 109, 103259. 10.1016/j.dnarep.2021.103259 34871862

[B80] Jairajpuri D. S. Mohammad T. Hussain A. Alajmi M. F. Yadav D. K. Hassan M. I. (2024). Identification of high-affinity inhibitors of TANK-binding kinase 1 (TBK1): a promising frontier for controlling inflammatory signaling in cancer. Discov. Med. 36 (180), 129–139. 10.24976/Discov.Med.202436180.12 38273753

[B81] Jia M. Wei Z. Liu P. Zhao X. (2016). Silencing of ABCG2 by microRNA-3163 inhibits multidrug resistance in retinoblastoma cancer stem cells. J. Korean Med. Sci. 31 (6), 836–842. 10.3346/jkms.2016.31.6.836 27247490 PMC4853660

[B82] Jin Y. Wang H. Zhu Y. Feng H. Wang G. Wang S. (2020). miR-199a-5p is involved in doxorubicin resistance of non-small cell lung cancer (NSCLC) cells. Eur. J. Pharmacol. 878, 173105. 10.1016/j.ejphar.2020.173105 32278855

[B83] Karabicici M. Alptekin S. Fırtına Karagonlar Z. Erdal E. (2021). Doxorubicin-induced senescence promotes stemness and tumorigenicity in EpCAM−/CD133− nonstem cell population in hepatocellular carcinoma cell line, HuH-7. Mol. Oncol. 15 (8), 2185–2202. 10.1002/1878-0261.12916 33524223 PMC8334288

[B84] Kciuk M. Gielecińska A. Mujwar S. Kołat D. Kałuzińska-Kołat Ż. Celik I. (2023). Doxorubicin—an agent with multiple mechanisms of anticancer activity. Cells 12 (4), 659. 10.3390/cells12040659 36831326 PMC9954613

[B85] Kim J.-Y. Jung E. J. Kim J.-M. Son Y. Lee H. S. Kwag S.-J. (2023). MiR-221 and miR-222 regulate cell cycle progression and affect chemosensitivity in breast cancer by targeting ANXA3. Exp. Ther. Med. 25 (3), 127. 10.3892/etm.2023.11826 36845963 PMC9947582

[B86] Kirtonia A. Sethi G. Garg M. (2020). The multifaceted role of reactive oxygen species in tumorigenesis. Cell. Mol. Life Sci. 77, 4459–4483. 10.1007/s00018-020-03536-5 32358622 PMC11105050

[B87] Kong F. B. Deng Q. M. Deng H. Q. Dong C. C. Li L. He C. G. (2020). Siva-1 regulates multidrug resistance of gastric cancer by targeting MDR1 and MRP1 via the NF-κB pathway. Mol. Med. Rep. 22 (2), 1558–1566. 10.3892/mmr.2020.11211 32626967 PMC7339453

[B88] Kovalchuk O. Filkowski J. Meservy J. Ilnytskyy Y. Tryndyak V. P. Chekhun V. F. (2008). Involvement of microRNA-451 in resistance of the MCF-7 breast cancer cells to chemotherapeutic drug doxorubicin. Mol. cancer Ther. 7 (7), 2152–2159. 10.1158/1535-7163.MCT-08-0021 18645025

[B89] Kratzer T. B. Bandi P. Freedman N. D. Smith R. A. Travis W. D. Jemal A. (2024). Lung cancer statistics, 2023. Cancer 130 (8), 1330–1348. 10.1002/cncr.35128 38279776

[B90] Kroemer G. McQuade J. L. Merad M. André F. Zitvogel L. (2023). Bodywide ecological interventions on cancer. Nat. Med. 29 (1), 59–74. 10.1038/s41591-022-02193-4 36658422

[B91] Kruck S. Eyrich C. Scharpf M. Sievert K. D. Fend F. Stenzl A. (2013). Impact of an altered Wnt1/β-catenin expression on clinicopathology and prognosis in clear cell renal cell carcinoma. Int. J. Mol. Sci. 14 (6), 10944–10957. 10.3390/ijms140610944 23708097 PMC3709711

[B92] Kruh G. D. Belinsky M. G. (2003). The MRP family of drug efflux pumps. Oncogene 22 (47), 7537–7552. 10.1038/sj.onc.1206953 14576857

[B93] Kuipers E. J. Grady W. M. Lieberman D. Seufferlein T. Sung J. J. Boelens P. G. (2015). Colorectal cancer. Colorectal cancer. Nat. Rev. Dis. prim. 1, 15065. 10.1038/nrdp.2015.65 27189416 PMC4874655

[B94] Ladd A. D. Duarte S. Sahin I. Zarrinpar A. (2024). Mechanisms of drug resistance in HCC. Hepatology 79 (4), 926–940. 10.1097/HEP.0000000000000237 36680397

[B95] Lally B. E. Urbanic J. J. Blackstock A. W. Miller A. A. Perry M. C. (2007). Small cell lung cancer: have we made any progress over the last 25 years? Oncologist 12 (9), 1096–1104. 10.1634/theoncologist.12-9-1096 17914079

[B96] Lao J. Madani J. Puértolas T. Álvarez M. Hernández A. Pazo-Cid R. (2013). Liposomal doxorubicin in the treatment of breast cancer patients: a review. J. drug Deliv. 2013, 456409. 10.1155/2013/456409 23634302 PMC3619536

[B97] Larasati Y. Boudou C. Koval A. Katanaev V. L. (2022). Unlocking the Wnt pathway: therapeutic potential of selective targeting FZD7 in cancer. Drug Discov. Today 27 (3), 777–792. 10.1016/j.drudis.2021.12.008 34915171

[B98] Lei Y. He X. Li J. Mo C. (2024). Drug resistance in hepatocellular carcinoma: theoretical basis and therapeutic aspects. FBL 29 (2), 52–null. 10.31083/j.fbl2902052 38420802

[B99] Leonard G. D. Fojo T. Bates S. E. (2003). The role of ABC transporters in clinical practice. Oncol. 8 (5), 411–424. 10.1634/theoncologist.8-5-411 14530494

[B100] Li D. Hong X. Zhao F. Ci X. Zhang S. (2021b). Targeting Nrf2 may reverse the drug resistance in ovarian cancer. Cancer Cell Int. 21 (1), 116. 10.1186/s12935-021-01822-1 33596893 PMC7890806

[B101] Li D. Huang Y. Wang G. (2021a). Circular RNA circPVT1 contributes to doxorubicin (DXR) resistance of osteosarcoma cells by regulating TRIAP1 via miR-137. Biomed. Res. Int. 2021, 7463867. 10.1155/2021/7463867 33981772 PMC8088374

[B102] Li M. Li Z. H. Song J. Li X. Zhai P. Mu X. (2022). miR-205 reverses MDR-1 mediated doxorubicin resistance via PTEN in human liver cancer HepG2 cells. Cell J. 24 (3), 112–119. 10.22074/cellj.2022.7231 35451580 PMC9035231

[B103] Li S. Yang J. Wang J. Gao W. Ding Y. Ding Y. (2018). Down-regulation of miR-210-3p encourages chemotherapy resistance of renal cell carcinoma via modulating ABCC1. Cell and Biosci. 8 (1), 9. 10.1186/s13578-018-0209-3 PMC5803904 29445446

[B104] Li Y. Zhao L. Li N. Miao Y. Zhou H. Jia L. (2017). miR-9 regulates the multidrug resistance of chronic myelogenous leukemia by targeting ABCB1. Oncol. Rep. 37 (4), 2193–2200. 10.3892/or.2017.5464 28260112

[B105] Liao H. Bao X. Zhu J. Qu J. Sun Y. Ma X. (2015). O-Alkylated derivatives of quercetin induce apoptosis of MCF-7 cells via a caspase-independent mitochondrial pathway. Chem. Biol. Interact. 242, 91–98. 10.1016/j.cbi.2015.09.022 26415619

[B106] Lin S. Gregory R. I. (2015). MicroRNA biogenesis pathways in cancer. Nat. Rev. cancer 15 (6), 321–333. 10.1038/nrc3932 25998712 PMC4859809

[B107] Liu Y. Ao X. Ji G. Zhang Y. Yu W. Wang J. (2021a). Mechanisms of action and clinical implications of MicroRNAs in the drug resistance of gastric cancer. Front. Oncol. 11, 768918. 10.3389/fonc.2021.768918 34912714 PMC8667691

[B108] Liu Y. Liu X. Yang S. (2021b). MicroRNA-221 upregulates the expression of P-gp and Bcl-2 by activating the Stat3 pathway to promote doxorubicin resistance in osteosarcoma cells. Biol. Pharm. Bull. 44 (6), 861–868. 10.1248/bpb.b21-00163 33828027

[B109] Liu Y. Zhang H. Fang Y. Tang D. Luo Z. (2023). Non-coding RNAs in renal cell carcinoma: implications for drug resistance. Biomed. and Pharmacother. 164, 115001. 10.1016/j.biopha.2023.115001 37315433

[B110] Long Q.-Z. Du Y.-F. Liu X.-G. Li X. He D.-L. (2015). miR-124 represses FZD5 to attenuate P-glycoprotein-mediated chemo-resistance in renal cell carcinoma. Tumor Biol. 36 (9), 7017–7026. 10.1007/s13277-015-3369-3 25861751

[B111] Lotfi N. Yousefi Z. Golabi M. Khalilian P. Ghezelbash B. Montazeri M. (2023). The potential anti-cancer effects of quercetin on blood, prostate and lung cancers: an update. Front. Immunol. 14, 1077531. 10.3389/fimmu.2023.1077531 36926328 PMC10011078

[B112] Louisa M. Soediro T. M. Suyatna F. D. (2014). *In vitro* modulation of P-glycoprotein, MRP-1 and BCRP expression by mangiferin in doxorubicin-treated MCF-7 cells. Asian Pac. J. Cancer Prev. 15 (4), 1639–1642. 10.7314/apjcp.2014.15.4.1639 24641381

[B113] Lovitt C. J. Shelper T. B. Avery V. M. (2018). Doxorubicin resistance in breast cancer cells is mediated by extracellular matrix proteins. BMC Cancer 18 (1), 41. 10.1186/s12885-017-3953-6 29304770 PMC5756400

[B114] Lu L. Ju F. Zhao H. Ma X. (2015). MicroRNA-134 modulates resistance to doxorubicin in human breast cancer cells by downregulating ABCC1. Biotechnol. Lett. 37, 2387–2394. 10.1007/s10529-015-1941-y 26318721

[B115] Lu Q. Chen W. Ji Y. Liu Y. Xue X. (2022). Ursolic acid enhances cytotoxicity of doxorubicin-resistant triple-negative breast cancer cells via ZEB1-AS1/miR-186-5p/ABCC1 Axis. Cancer Biotherapy Radiopharm. 37 (8), 673–683. 10.1089/cbr.2020.4147 33493421

[B116] Lu X. Liu R. Wang M. Kumar A. K. Pan F. He L. (2020). MicroRNA-140 impedes DNA repair by targeting FEN1 and enhances chemotherapeutic response in breast cancer. Oncogene 39 (1), 234–247. 10.1038/s41388-019-0986-0 31471584

[B117] Lv J. Fu Z. Shi M. Xia K. Ji C. Xu P. (2015). Systematic analysis of gene expression pattern in has-miR-760 overexpressed resistance of the MCF-7 human breast cancer cell to doxorubicin. Biomed. and Pharmacother. 69, 162–169. 10.1016/j.biopha.2014.11.028 25661353

[B118] Lv L. An X. Li H. Ma L. (2016). Effect of miR-155 knockdown on the reversal of doxorubicin resistance in human lung cancer A549/dox cells. Oncol. Lett. 11 (2), 1161–1166. 10.3892/ol.2015.3995 26893712 PMC4734087

[B119] Ma J. Wang T. Guo R. Yang X. Yin J. Yu J. (2015). Involvement of miR-133a and miR-326 in ADM resistance of HepG2 through modulating expression of ABCC1. J. Drug Target. 23 (6), 519–524. 10.3109/1061186X.2015.1015536 25714665

[B120] Maharati A. Moghbeli M. (2023). Role of microRNAs in regulation of doxorubicin and paclitaxel responses in lung tumor cells. Cell Div. 18 (1), 11. 10.1186/s13008-023-00093-8 37480054 PMC10362644

[B121] Michlewski G. Cáceres J. F. (2019). Post-transcriptional control of miRNA biogenesis. Rna 25 (1), 1–16. 10.1261/rna.068692.118 30333195 PMC6298569

[B122] Moore J. M. Bell E. L. Hughes R. O. Garfield A. S. (2023). ABC transporters: human disease and pharmacotherapeutic potential. Trends Mol. Med. 29 (2), 152–172. 10.1016/j.molmed.2022.11.001 36503994

[B123] Moyal L. Goldfeiz N. Gorovitz B. Rephaeli A. Tal E. Tarasenko N. (2018). AN-7, a butyric acid prodrug, sensitizes cutaneous T-cell lymphoma cell lines to doxorubicin via inhibition of DNA double strand breaks repair. Invest. New Drugs 36 (1), 1–9. 10.1007/s10637-017-0500-x 28884410

[B124] Naso F. D. Bruqi K. Manzini V. Chiurchiù V. D’Onofrio M. Arisi I. (2024). miR-218-5p and doxorubicin combination enhances anticancer activity in breast cancer cells through Parkin-dependent mitophagy inhibition. Cell Death Discov. 10 (1), 149. 10.1038/s41420-024-01914-7 38514650 PMC10957887

[B125] Nazari Soltan Ahmad S. Sanajou D. Kalantary‐Charvadeh A. Hosseini V. Roshangar L. Khojastehfard M. (2020). β-LAPachone ameliorates doxorubicin-induced cardiotoxicity via regulating autophagy and Nrf2 signalling pathways in mice. Basic and Clin. Pharmacol. and Toxicol. 126 (4), 364–373. 10.1111/bcpt.13340 31630478

[B126] Okamura K. Phillips M. D. Tyler D. M. Duan H. Chou Y. Lai E. C. (2008). The regulatory activity of microRNA* species has substantial influence on microRNA and 3′ UTR evolution. Nat. Struct. and Mol. Biol. 15 (4), 354–363. 10.1038/nsmb.1409 18376413 PMC2698667

[B127] Ou Y. Zhang K. Shuai Q. Wang C. Hu H. Cao L. (2024). Abstract 4556: USP51-GRP78-ABCB1 axis promotes chemoresistance of triple negative breast cancer by decreasing the accumulation of doxorubicin in cells. Cancer Res. 84 (6_Suppl. ment), 4556. 10.1158/1538-7445.am2024-4556

[B128] Packeiser E.-M. Engels L. Nolte I. Goericke-Pesch S. Murua Escobar H. (2023). MDR1 inhibition reverses doxorubicin-resistance in six doxorubicin-resistant canine prostate and bladder cancer cell lines. Int. J. Mol. Sci. 24 (9), 8136. 10.3390/ijms24098136 37175843 PMC10179448

[B129] Pan X. Hong X. Li S. Meng P. Xiao F. (2021). METTL3 promotes adriamycin resistance in MCF-7 breast cancer cells by accelerating pri-microRNA-221-3p maturation in a m6A-dependent manner. Exp. and Mol. Med. 53 (1), 91–102. 10.1038/s12276-020-00510-w 33420414 PMC8080609

[B130] Park S.-J. Wu C.-H. Safa A. R. (2004). A P-glycoprotein- and MRP1-independent doxorubicin-resistant variant of the MCF-7 breast cancer cell line with defects in caspase-6, -7, -8, -9 and -10 activation pathways. Anticancer Res. 24 (1), 123–131. 15015586

[B131] Ponnusamy L. Mahalingaiah P. K. S. Chang Y.-W. Singh K. P. (2018). Reversal of epigenetic aberrations associated with the acquisition of doxorubicin resistance restores drug sensitivity in breast cancer cells. Eur. J. Pharm. Sci. 123, 56–69. 10.1016/j.ejps.2018.07.028 30016648

[B132] Qin K. Yu M. Fan J. Wang H. Zhao P. Zhao G. (2024). Canonical and noncanonical Wnt signaling: multilayered mediators, signaling mechanisms and major signaling crosstalk. Genes and Dis. 11 (1), 103–134. 10.1016/j.gendis.2023.01.030 PMC10425814 37588235

[B133] Raskova Kafkova L. Mierzwicka J. M. Chakraborty P. Jakubec P. Fischer O. Skarda J. (2024). NSCLC: from tumorigenesis, immune checkpoint misuse to current and future targeted therapy. Front. Immunol. 15, 1342086. 10.3389/fimmu.2024.1342086 38384472 PMC10879685

[B134] Reddy K. B. (2015). MicroRNA (miRNA) in cancer. Cancer Cell Int. 15 (1), 38–46. 10.1186/s12935-015-0185-1 25960691 PMC4424445

[B135] Romero-Cordoba S. L. Salido-Guadarrama I. Rodriguez-Dorantes M. Hidalgo-Miranda A. (2014). miRNA biogenesis: biological impact in the development of cancer. Cancer Biol. and Ther. 15 (11), 1444–1455. 10.4161/15384047.2014.955442 25482951 PMC4622859

[B136] Sadighi S. Sharifian R. Kazemimanesh M. Muhammadnejad A. Shahosseini Z. Amanpour S. (2021). Down-regulation of immune checkpoints by doxorubicin and carboplatin-containing neoadjuvant regimens in a murine breast cancer model. Iran. J. Basic Med. Sci. 24 (4), 537–544. 10.22038/ijbms.2021.54383.12221 34094037 PMC8143709

[B137] Safaei S. Amini M. Najjary S. Mokhtarzadeh A. Bolandi N. Saeedi H. (2022). miR-200c increases the sensitivity of breast cancer cells to Doxorubicin through downregulating MDR1 gene. Exp. Mol. Pathology 125, 104753. 10.1016/j.yexmp.2022.104753 35235816

[B138] Sajid A. Rahman H. Ambudkar S. V. (2023). Advances in the structure, mechanism and targeting of chemoresistance-linked ABC transporters. Nat. Rev. Cancer 23 (11), 762–779. 10.1038/s41568-023-00612-3 37714963

[B139] Salvatore L. Calegari M. A. Loupakis F. Fassan M. Di Stefano B. Bensi M. (2019). PTEN in colorectal cancer: shedding light on its role as predictor and target. Cancers 11 (11), 1765. 10.3390/cancers11111765 31717544 PMC6896095

[B140] Sansregret L. Vanhaesebroeck B. Swanton C. (2018). Determinants and clinical implications of chromosomal instability in cancer. Nat. Rev. Clin. Oncol. 15 (3), 139–150. 10.1038/nrclinonc.2017.198 29297505

[B141] Sarwar S. Morozov V. M. Newcomb M. A. Yan B. Brant J. O. Opavsky R. (2024). Overcoming ABCB1 mediated multidrug resistance in castration resistant prostate cancer. Cell Death and Dis. 15 (8), 558. 10.1038/s41419-024-06949-3 PMC11294535 39090086

[B142] Schulz J. A. Hartz A. M. S. Bauer B. (2023). ABCB1 and ABCG2 regulation at the blood-brain barrier: potential new targets to improve brain drug delivery. Pharmacol. Rev. 75 (5), 815–853. 10.1124/pharmrev.120.000025 36973040 PMC10441638

[B143] Selvarajan S. Vijayaraghavan J. Bobby Z. Ramalingam J. Porur C. (2019). Micro RNAs—a review. J. Evol. Med. Dent. Sci. 8, 2918–2923. 10.14260/jemds/2019/634

[B144] Seyhan A. A. (2024). Trials and tribulations of MicroRNA therapeutics. Int. J. Mol. Sci. 25 (3), 1469. 10.3390/ijms25031469 38338746 PMC10855871

[B145] Shaikh S. Shaikh J. Naba Y. S. Doke K. Ahmed K. Yusufi M. (2021). Curcumin: reclaiming the lost ground against cancer resistance. Cancer Drug Resist 4 (2), 298–320. 10.20517/cdr.2020.92 35582033 PMC9019276

[B146] Shang Y. Zhang Z. Liu Z. Feng B. Ren G. Li K. (2014). miR-508-5p regulates multidrug resistance of gastric cancer by targeting ABCB1 and ZNRD1. Oncogene 33 (25), 3267–3276. 10.1038/onc.2013.297 23893241

[B147] Shi X. Valizadeh A. Mir S. M. Asemi Z. Karimian A. Majidina M. (2020). miRNA-29a reverses P-glycoprotein-mediated drug resistance and inhibits proliferation via up-regulation of PTEN in colon cancer cells. Eur. J. Pharmacol. 880, 173138. 10.1016/j.ejphar.2020.173138 32416187

[B148] Shi Y. Zhang Y. Zhao Y. Hong L. Liu N. Jin X. (2004). Overexpression of ZNRD1 promotes multidrug-resistant phenotype of gastric cancer cells through upregulation of P-glycoprotein. Cancer Biol. Ther. 3 (4), 377–381. 10.4161/cbt.3.4.724 14726695

[B149] Siegel R. Ma J. Zou Z. Jemal A. (2014). Cancer statistics, 2014. CA a cancer J. Clin. 64 (1), 9–29. 10.3322/caac.21208 24399786

[B150] Siegel R. L. Miller K. D. Wagle N. S. Jemal A. (2023). Cancer statistics, 2023. Ca Cancer J. Clin. 73 (1), 17–48. 10.3322/caac.21763 36633525

[B151] Sohail M. Sun Z. Li Y. Gu X. Xu H. (2021). Research progress in strategies to improve the efficacy and safety of doxorubicin for cancer chemotherapy. Expert Rev. Anticancer Ther. 21 (12), 1385–1398. 10.1080/14737140.2021.1991316 34636282

[B152] Sparreboom A. Nooter K. (2000). Does P-glycoprotein play a role in anticancer drug pharmacokinetics? Drug Resist. Updat. 3 (6), 357–363. 10.1054/drup.2000.0164 11498403

[B153] Sritharan S. Sivalingam N. (2021). A comprehensive review on time-tested anticancer drug doxorubicin. Life Sci. 278, 119527. 10.1016/j.lfs.2021.119527 33887349

[B154] Stern S. Kurian R. Wang H. (2022). Clinical relevance of the constitutive androstane receptor. Drug Metab. Dispos. 50 (7), 1010–1018. 10.1124/dmd.121.000483 35236665 PMC11022901

[B155] Sun H. Sun Y. Chen Q. Xu Z. (2020). LncRNA KCNQ1OT1 contributes to the progression and chemoresistance in acute myeloid leukemia by modulating Tspan3 through suppressing miR-193a-3p. Life Sci. 241, 117161. 10.1016/j.lfs.2019.117161 31837329

[B156] Szakács G. Paterson J. K. Ludwig J. A. Booth-Genthe C. Gottesman M. M. (2006). Targeting multidrug resistance in cancer. Nat. Rev. Drug Discov. 5 (3), 219–234. 10.1038/nrd1984 16518375

[B157] Takwi A. A. Wang Y. M. Wu J. Michaelis M. Cinatl J. Chen T. (2014). miR-137 regulates the constitutive androstane receptor and modulates doxorubicin sensitivity in parental and doxorubicin-resistant neuroblastoma cells. Oncogene 33 (28), 3717–3729. 10.1038/onc.2013.330 23934188 PMC3922897

[B158] Tao L. Shu-Ling W. Jing-Bo H. Ying Z. Rong H. Xiang-Qun L. (2020). MiR-451a attenuates doxorubicin resistance in lung cancer via suppressing epithelialmesenchymal transition (EMT) through targeting c-Myc. Biomed. and Pharmacother. 125, 109962. 10.1016/j.biopha.2020.109962 32106373

[B159] Tawfik S. A. Awad E. T. Bakr H. O. A. Ahmed I. M. Ashour E. Gamal-Eldeen A. M. (2023). Chia seeds oil suppresses the resistance of hepatocellular carcinoma cells to liposomal-doxorubicin and upregulates the tumor suppressor miRNAs. Curr. Pharm. Biotechnol. 24 (4), 570–578. 10.2174/1389201023666220921125258 36154592

[B160] Taymaz-Nikerel H. Karabekmez M. E. Eraslan S. Kırdar B. (2018). Doxorubicin induces an extensive transcriptional and metabolic rewiring in yeast cells. Sci. Rep. 8 (1), 13672. 10.1038/s41598-018-31939-9 30209405 PMC6135803

[B161] Teng R. Hu Y. Zhou J. Seifer B. Chen Y. Shen J. (2015). Overexpression of Lin28 decreases the chemosensitivity of gastric cancer cells to oxaliplatin, paclitaxel, doxorubicin, and fluorouracil in part via microRNA-107. PLOS ONE 10 (12), e0143716. 10.1371/journal.pone.0143716 26636340 PMC4670127

[B162] To K. K. W. Huang Z. Zhang H. Ashby C. R. Fu L. (2024). Utilizing non-coding RNA-mediated regulation of ATP binding cassette (ABC) transporters to overcome multidrug resistance to cancer chemotherapy. Drug Resist. Updat. 73, 101058. 10.1016/j.drup.2024.101058 38277757

[B163] Toh M. R. Wong E. Y. T. Wong S. H. Ng A. W. T. Loo L.-H. Chow P. K.-H. (2023). Global epidemiology and genetics of hepatocellular carcinoma. Gastroenterology 164 (5), 766–782. 10.1053/j.gastro.2023.01.033 36738977

[B164] Valter K. Zhivotovsky B. Gogvadze V. (2018). Cell death-based treatment of neuroblastoma. Cell Death and Dis. 9 (2), 113. 10.1038/s41419-017-0060-1 PMC5833874 29371588

[B165] Vasiliou V. Vasiliou K. Nebert D. W. (2009). Human ATP-binding cassette (ABC) transporter family. Hum. Genomics 3 (3), 281–290. 10.1186/1479-7364-3-3-281 19403462 PMC2752038

[B166] Veschi V. Durinck K. Thiele C. J. Speleman F. (2024). “Neuroblastoma epigenetic landscape: drugging opportunities,” in Neuroblastoma: discoveries and challenges. Editors Asgharzadeh S. Westermann F. (Cham: Springer International Publishing), 71–95.

[B167] Vincan E. Flanagan D. J. Pouliot N. Brabletz T. Spaderna S. (2010). Variable FZD7 expression in colorectal cancers indicates regulation by the tumour microenvironment. Dev. Dyn. 239 (1), 311–317. 10.1002/dvdy.22045 19655379

[B168] Vishnoi A. Rani S. (2017). MiRNA biogenesis and regulation of diseases: an overview. MicroRNA Profiling Methods Protoc. 1509, 1–10. 10.1007/978-1-4939-6524-3_1 27826912

[B169] Wang B. Yan L. Shi W. Xie H. Chen R. Shao Y. (2022a). CircRNA PVT1 promotes proliferation and chemoresistance of osteosarcoma cells via the miR-24-3p/KLF8 axis. Int. J. Clin. Oncol. 27 (4), 811–822. 10.1007/s10147-022-02122-y 35171359

[B170] Wang F. Li T. Zhang B. Li H. Wu Q. Yang L. (2013). MicroRNA-19a/b regulates multidrug resistance in human gastric cancer cells by targeting PTEN. Biochem. Biophysical Res. Commun. 434 (3), 688–694. 10.1016/j.bbrc.2013.04.010 23603256

[B171] Wang L. Lu B. He M. Wang Y. Wang Z. Du L. (2022b). Prostate cancer incidence and mortality: global status and temporal trends in 89 countries from 2000 to 2019. Front. Public Health 10, 811044. 10.3389/fpubh.2022.811044 35252092 PMC8888523

[B172] Wang Q. Su C. Li J. Wei C. (2018). Mechanism of the enhancing effects of miR-93 on resistance of breast cancer MCF-7 cells to adriamycin. Oncol. Lett. 16 (3), 3779–3783. 10.3892/ol.2018.9054 30127988 PMC6096162

[B173] Wang X. Ding R. Fu Z. Yang M. Li D. Zhou Y. (2024). Overexpression of miR-506-3p reversed doxorubicin resistance in drug-resistant osteosarcoma cells. Front. Pharmacol. 15, 1303732. 10.3389/fphar.2024.1303732 38420199 PMC10899521

[B174] Wang X. Sykes D. B. Miller D. S. (2010). Constitutive androstane receptor-mediated up-regulation of ATP-driven xenobiotic efflux transporters at the blood-brain barrier. Mol. Pharmacol. 78 (3), 376–383. 10.1124/mol.110.063685 20547735 PMC2939489

[B175] Wang Y. Wang Y. Qin Z. Cai S. Yu L. Hu H. (2021). The role of non-coding RNAs in ABC transporters regulation and their clinical implications of multidrug resistance in cancer. Expert Opin. Drug Metabolism and Toxicol. 17 (3), 291–306. 10.1080/17425255.2021.1887139 33544643

[B176] Wilson B. J. Schatton T. Zhan Q. Gasser M. Ma J. Saab K. R. (2011). ABCB5 identifies a therapy-refractory tumor cell population in colorectal cancer patients. Cancer Res. 71 (15), 5307–5316. 10.1158/0008-5472.CAN-11-0221 21652540 PMC3395026

[B177] Wu D. Zhang J. Lu Y. Bo S. Li L. Wang L. (2019). miR-140-5p inhibits the proliferation and enhances the efficacy of doxorubicin to breast cancer stem cells by targeting Wnt1. Cancer Gene Ther. 26 (3), 74–82. 10.1038/s41417-018-0035-0 30032164

[B178] Wu H.-T. Chen W.-T. Li G.-W. Shen J.-X. Ye Q.-Q. Zhang M.-L. (2020). Analysis of the differentially expressed genes induced by cisplatin resistance in oral squamous cell carcinomas and their interaction. Front. Genet. 10, 1328. 10.3389/fgene.2019.01328 32038705 PMC6989555

[B179] Wuxiao Z. Wang H. Su Q. Zhou H. Hu M. Tao S. (2020). MicroRNA-145 promotes the apoptosis of leukemic stem cells and enhances drug-resistant K562/ADM cell sensitivity to adriamycin via the regulation of ABCE1. Int. J. Mol. Med. 46 (4), 1289–1300. 10.3892/ijmm.2020.4675 32945355 PMC7447303

[B180] Xi L. Liu Q. Zhang W. Luo L. Song J. Liu R. (2021). Circular RNA circCSPP1 knockdown attenuates doxorubicin resistance and suppresses tumor progression of colorectal cancer via miR-944/FZD7 axis. Cancer Cell Int. 21 (1), 153. 10.1186/s12935-021-01855-6 33663510 PMC7934234

[B181] Xia X. Wang Q. Ye T. Liu Y. Liu D. Song S. (2020). NRF 2/ABCB 1-mediated efflux and PARP 1-mediated dampening of DNA damage contribute to doxorubicin resistance in chronic hypoxic HepG2 cells. Fundam. and Clin. Pharmacol. 34 (1), 41–50. 10.1111/fcp.12505 31420991

[B182] Xiao H. Zheng Y. Ma L. Tian L. Sun Q. (2021). Clinically-relevant ABC transporter for anti-cancer drug resistance. Front. Pharmacol. 12, 648407. 10.3389/fphar.2021.648407 33953682 PMC8089384

[B183] Xie B. Li L. Zhang Z. Zhao L. Cheng J. Zhou C. (2021). MicroRNA-1246 by targeting AXIN2 and GSK-3β overcomes drug resistance and induces apoptosis in chemo-resistant leukemia cells. J. Cancer 12 (14), 4196–4208. 10.7150/jca.58522 34093820 PMC8176421

[B184] Xie M. Fu Z. Cao J. Liu Y. Wu J. Li Q. (2018a). MicroRNA-132 and microRNA-212 mediate doxorubicin resistance by down-regulating the PTEN-AKT/NF-κB signaling pathway in breast cancer. Biomed. and Pharmacother. 102, 286–294. 10.1016/j.biopha.2018.03.088 29567542

[B185] Xie Q. Wang S. Zhao Y. Zhang Z. Qin C. Yang X. (2017). MiR-519d impedes cisplatin-resistance in breast cancer stem cells by down-regulating the expression of MCL-1. Oncotarget 8 (13), 22003–22013. 10.18632/oncotarget.15781 28423543 PMC5400641

[B186] Xie Z.-Y. Liu M.-S. Zhang C. Cai P.-C. Xiao Z.-H. Wang F.-F. (2018b). Aspirin enhances the sensitivity of hepatocellular carcinoma side population cells to doxorubicin via miR-491/ABCG2. Biosci. Rep. 38 (6), BSR20180854. 10.1042/BSR20180854 30120100 PMC6239265

[B187] Xiong S. Xiao G. W. (2018). Reverting doxorubicin resistance in colon cancer by targeting a key signaling protein, steroid receptor coactivator. Exp. Ther. Med. 15 (4), 3751–3758. 10.3892/etm.2018.5912 29581735 PMC5863609

[B188] Xu C. Shen J. Xie S. Jiang Z. Huang L. Wang L. (2013). Positive expression of Lin28 is correlated with poor survival in gastric carcinoma. Med. Oncol. 30, 382–386. 10.1007/s12032-012-0382-x 23292830

[B189] Xu P. Li C. Yuan J. Bao Z. Liu W. (2024). Predict lncRNA-drug associations based on graph neural network. Front. Genet. 15, 1388015. 10.3389/fgene.2024.1388015 38737125 PMC11082279

[B190] Xu Q. Krause M. Samoylenko A. Vainio S. (2016). Wnt signaling in renal cell carcinoma. Cancers 8 (6), 57. 10.3390/cancers8060057 27322325 PMC4931622

[B191] Xu Y. Xia F. Ma L. Shan J. Shen J. Yang Z. (2011). MicroRNA-122 sensitizes HCC cancer cells to adriamycin and vincristine through modulating expression of MDR and inducing cell cycle arrest. Cancer Lett. 310 (2), 160–169. 10.1016/j.canlet.2011.06.027 21802841

[B192] Yahya S. M. M. Fathy S. A. El-Khayat Z. A. El-Toukhy S. E. Hamed A. R. Hegazy M. G. A. (2018). Possible role of microRNA-122 in modulating multidrug resistance of hepatocellular carcinoma. Indian J. Clin. Biochem. 33 (1), 21–30. 10.1007/s12291-017-0651-8 29371766 PMC5766467

[B193] Yahya S. M. M. Nabih H. K. Elsayed G. H. Mohamed S. I. A. Elfiky A. M. Salem S. M. (2024). Restoring microRNA-34a overcomes acquired drug resistance and disease progression in human breast cancer cell lines via suppressing the ABCC1 gene. Breast Cancer Res. Treat. 204 (1), 133–149. 10.1007/s10549-023-07170-0 38057687 PMC10806220

[B194] Yan H. Bu P. (2021). Non-coding RNA in cancer. Essays Biochem. 65 (4), 625–639. 10.1042/EBC20200032 33860799 PMC8564738

[B195] Yang G. Jiang O. Ling D. Jiang X. Yuan P. Zeng G. (2015). MicroRNA-522 reverses drug resistance of doxorubicin-induced HT29 colon cancer cell by targeting ABCB5. Mol. Med. Rep. 12 (3), 3930–3936. 10.3892/mmr.2015.3890 26043974

[B196] Yang S. Shim M. K. Kim W. J. Choi J. Nam G.-H. Kim J. (2021). Cancer-activated doxorubicin prodrug nanoparticles induce preferential immune response with minimal doxorubicin-related toxicity. Biomaterials 272, 120791. 10.1016/j.biomaterials.2021.120791 33831739

[B197] Yang S. Wang X. Zhou X. Hou L. Wu J. Zhang W. (2023). ncRNA-mediated ceRNA regulatory network: transcriptomic insights into breast cancer progression and treatment strategies. Biomed. and Pharmacother. 162, 114698. 10.1016/j.biopha.2023.114698 37060661

[B198] Yang T. Zheng Z. Li X. Li Z. Wang Y. Geng Y. (2013). MiR-223 modulates multidrug resistance via downregulation of ABCB1 in hepatocellular carcinoma cells. Exp. Biol. Med. 238 (9), 1024–1032. 10.1177/1535370213497321 23925649

[B199] Ye J. Xu M. Tian X. Cai S. Zeng S. (2019). Research advances in the detection of miRNA. J. Pharm. analysis 9 (4), 217–226. 10.1016/j.jpha.2019.05.004 PMC6702429 31452959

[B200] Yi H. Liu L. Sheng N. Li P. Pan H. Cai L. (2016). Synergistic therapy of doxorubicin and miR-129-5p with self-cross-linked bioreducible polypeptide nanoparticles reverses multidrug resistance in cancer cells. Biomacromolecules 17 (5), 1737–1747. 10.1021/acs.biomac.6b00141 27029378

[B201] Yi J. Wang L. Hu G. Zhang Y. Du J. Ding J. (2023). CircPVT1 promotes ER‐positive breast tumorigenesis and drug resistance by targeting ESR1 and MAVS. EMBO J. 42 (10), e112408. 10.15252/embj.2022112408 37009655 PMC10183818

[B202] Ying Q. Fan R. Shen Y. Chen B. Zhang J. Li Q. (2024). Small cell lung cancer—an update on chemotherapy resistance. Curr. Treat. Options Oncol. 25, 1112–1123. 10.1007/s11864-024-01245-w 39066852

[B203] Young M. Jackson-Spence F. Beltran L. Day E. Suarez C. Bex A. (2024). Renal cell carcinoma. Lancet 404 (10451), 476–491. 10.1016/S0140-6736(24)00917-6 39033764

[B204] Yu J. Chen W.-X. Xie W.-J. Chen R.-W. Lin D.-Q. You W.-W. (2021). Silencing of the CrkL gene reverses the doxorubicin resistance of K562/ADR cells through regulating PI3K/Akt/MRP1 signaling. J. Clin. Laboratory Analysis 35 (8), e23817. 10.1002/jcla.23817 PMC8373353 34114685

[B205] Yu X. Du Z. Zhu P. Liao B. (2024). Diagnostic, prognostic, and therapeutic potential of exosomal microRNAs in renal cancer. Pharmacol. Rep. 76 (2), 273–286. 10.1007/s43440-024-00568-7 38388810

[B206] Yuan J. Xiao C. Lu H. Yu H. Hong H. Guo C. (2020). Effect of miR-12136 on drug sensitivity of drug-resistant cell line Michigan cancer foundation-7/doxorubicin by regulating ATP binding cassette subfamily B member 1. J. Biomaterials Tissue Eng. 10 (10), 1431–1435.

[B207] Zangouei A. S. Alimardani M. Moghbeli M. (2021). MicroRNAs as the critical regulators of Doxorubicin resistance in breast tumor cells. Cancer Cell Int. 21 (1), 213. 10.1186/s12935-021-01873-4 33858435 PMC8170947

[B208] Zeng C. Fan D. Xu Y. Li X. Yuan J. Yang Q. (2020). Curcumol enhances the sensitivity of doxorubicin in triple-negative breast cancer via regulating the miR-181b-2-3p-ABCC3 axis. Biochem. Pharmacol. 174, 113795. 10.1016/j.bcp.2020.113795 31926937

[B209] Zhang F. Lei X. Yang X. (2024). Emerging roles of ncRNAs regulating ABCC1 on chemotherapy resistance of cancer – a review. J. Chemother. 36 (1), 1–10. 10.1080/1120009X.2023.2247202 38263773

[B210] Zhang L. Shi H. Tan X. Jiang Z. Wang P. Qin J. (2022). Ten-gram-scale mechanochemical synthesis of ternary lanthanum coordination polymers for antibacterial and antitumor activities. Front. Chem. 10, 898324. 10.3389/fchem.2022.898324 35774860 PMC9237552

[B211] Zhang Z. Ha S. H. Moon Y. J. Hussein U. K. Song Y. Kim K. M. (2020). Inhibition of SIRT6 potentiates the anti-tumor effect of doxorubicin through suppression of the DNA damage repair pathway in osteosarcoma. J. Exp. and Clin. Cancer Res. 39 (1), 247. 10.1186/s13046-020-01759-9 33198792 PMC7670730

[B212] Zhao C. Tang X. Chen X. Jiang Z. (2024). Multifaceted carbonized metal-organic frameworks synergize with immune checkpoint inhibitors for precision and augmented cuproptosis cancer therapy. ACS Nano 18 (27), 17852–17868. 10.1021/acsnano.4c04022 38939981

[B213] Zhao J. Jiang G. (2020). Effect of dual-targeting MiR-4282 and ATP-binding cassette sub-family C member 4 on drug resistance of breast cancer cells and its molecular mechanism. J. Biomaterials Tissue Eng. 10, 507–511. 10.1166/jbt.2020.2289

[B214] Zhao L. Wang Y. Jiang L. He M. Bai X. Yu L. (2016). MiR-302a/b/c/d cooperatively sensitizes breast cancer cells to adriamycin via suppressing P-glycoprotein (P-gp) by targeting MAP/ERK kinase kinase 1 (MEKK1). J. Exp. and Clin. Cancer Res. 35 (1), 25–14. 10.1186/s13046-016-0300-8 26842910 PMC4738800

[B215] Zhao S. Pan T. Deng J. Cao L. Vicencio J. M. Liu J. (2023). Exosomal transfer of miR-181b-5p confers senescence-mediated doxorubicin resistance via modulating BCLAF1 in breast cancer. Br. J. Cancer 128 (4), 665–677. 10.1038/s41416-022-02077-x 36522479 PMC9938221

[B216] Zhao W. Ning L. Wang L. Ouyang T. Qi L. Yang R. (2021). miR-21 inhibition reverses doxorubicin-resistance and inhibits PC3 human prostate cancer cells proliferation. Andrologia 53 (5), e14016. 10.1111/and.14016 33598946

[B217] Zhao Y. Chen J. Wei W. Qi X. Li C. Ren J. (2018). The dual-inhibitory effect of miR-338-5p on the multidrug resistance and cell growth of hepatocellular carcinoma. Signal Transduct. Target. Ther. 3 (1), 3. 10.1038/s41392-017-0003-4 29527329 PMC5837112

[B218] Zhao Y. Qi X. Chen J. Wei W. Yu C. Yan H. (2017). The miR-491-3p/Sp3/ABCB1 axis attenuates multidrug resistance of hepatocellular carcinoma. Cancer Lett. 408, 102–111. 10.1016/j.canlet.2017.08.027 28844709

[B219] Zheng D. Dai Y. Wang S. Xing X. (2015). MicroRNA-299-3p promotes the sensibility of lung cancer to doxorubicin through directly targeting ABCE1. Int. J. Clin. Exp. Pathol. 8 (9), 10072–10081. 26617714 PMC4637529

[B220] Zheng S. Z. Sun P. Wang J. P. Liu Y. Gong W. Liu J. (2019). MiR-34a overexpression enhances the inhibitory effect of doxorubicin on HepG2 cells. World J. Gastroenterol. 25 (22), 2752–2762. 10.3748/wjg.v25.i22.2752 31235998 PMC6580351

[B221] Zhou Y. Li Q. Pan R. Wang Q. Zhu X. Yuan C. (2022). Regulatory roles of three miRNAs on allergen mRNA expression in Tyrophagus putrescentiae. Allergy 77 (2), 469–482. 10.1111/all.15111 34570913

[B222] Zhou Y. Zhao R. Tseng K.-F. Li K. Lu Z. Liu Y. (2016). Sirolimus induces apoptosis and reverses multidrug resistance in human osteosarcoma cells *in vitro* via increasing microRNA-34b expression. Acta Pharmacol. Sin. 37 (4), 519–529. 10.1038/aps.2015.153 26924291 PMC4820798

[B223] Zhu Y. Jiang Y. Shi L. Du L. Xu X. Wang E. (2017). 7-O-Geranylquercetin induces apoptosis in gastric cancer cells via ROS-MAPK mediated mitochondrial signaling pathway activation. Biomed. Pharmacother. 87, 527–538. 10.1016/j.biopha.2016.12.095 28076833

[B224] Zhu Y. Yu F. Jiao Y. Feng J. Tang W. Yao H. (2011). Reduced miR-128 in breast tumor–initiating cells induces chemotherapeutic resistance via bmi-1 and ABCC5. Clin. Cancer Res. 17 (22), 7105–7115. 10.1158/1078-0432.CCR-11-0071 21953503

[B225] Zou Z. Zou R. Zong D. Shi Y. Chen J. Huang J. (2017). miR‐495 sensitizes MDR cancer cells to the combination of doxorubicin and taxol by inhibiting MDR1 expression. J. Cell. Mol. Med. 21 (9), 1929–1943. 10.1111/jcmm.13114 28411377 PMC5571520

