# Supplementary material for: MiRNAs function in the development of resistance against doxorubicin in cancer cells: targeting ABC transporters
Source: Front Pharmacol. 2024 Nov 29;15:1486783. doi: 10.3389/fphar.2024.1486783 (PMC11638538; doi:10.3389/fphar.2024.1486783)
Supplement: Supplementary file 1 [file Table1.docx]

**Table 1 Interactions between miRNAs and ABC transporters in mediating doxorubicin resistance**

| **MiRNA** | **ABC transporters** | **Cell lines** | **Sample and cancer cell lines** | **Targeted** | **Highlights** | **Ref.** |
| --- | --- | --- | --- | --- | --- | --- |
| **Liver cancers** | | | | | | |
| miR-223 (Tumor suppressor) | ↓ABCB1(P-gp) | HCC  Hep3B, HCC3, LM-6, SMMC7721, Huh-7, SK-Hep-1, HepG2 and BEL-7402 | In vitro and in vivo | ↓ABCB1 | “miR-223 targeted ABCB1 3’UTR directly, and miR-223 downregulated ABCB1 at both mRNA and protein levels.” | (1) |
| miR-491-3p  (Tumor suppressor) | ↓ABCB1(P-gp) | HCC  Hep3B, BEL-7402 and SMMC-7721 | In vitro | microRNA-491/ ↓Sp3/↓ABCB1 | “miR-491-3p regulated P-gp expression via Sp3 pathway besides directly binding to the 3′-UTR of ABCB1. In combination, these data propose an important regulatory role for miR-491-3p in HCC cells, probably through regulating Sp3-dependent and –independent P-gp expression.” | (2) |
| miR-133a and miR-326  (Tumor suppressor) | ↓ABCC1 | HCC  HepG2 | In vitro | miR-133a and miR-326/↓ABCC1 | “miR-133a or miR-326 mimics transfection sensitized cancer cells to Adriamycin through targeting 3′untranslated region (3′UTR) of ABCC1 and suppressing its expression.” | (3) |
| miR-122  (Tumor suppressor) | ↓ABCB1, ↓ABCC1, ↓ABCG2 and ↓ABCF2 | HCC  HepG2 | In vitro | miR-122/↓ABCB1, ↓ABCC1, ↓ABCG2 and ↓ABCF2 | “ Overexpression of miR-122 inhibited HCC cell growth by inducing cell cycle arrest and this arrest is associated with down-regulation of MDR-related genes like as ABCB1, ABCC1, ABCG2 and ABCF2.” | (4) |
| miR-34a  (Tumor suppressor) | ↓ABCB1(P-gp) | HCC  HepG2 | In vitro | miR-34a /↑p53 /↓ABCB1, ↓cyclin D1, ↓CDK4 and ↓CDK6, ↓Bcl-2 | “MiR-34a may enhance the inhibitory effect of doxorubicin by downregulating MDR1/P-gp and AXL, which may be related to p53 expression.” | (5) |
| miR-491  (Tumor suppressor) | ↓ABCG2 | HCC  MHCC-97L | In vitro | Aspirin/↑miR-491/↓ABCG2 | “Aspirin enhanced the sensitivity of HCC SP cells to Doxo via up-regulating miR-491 and down-regulating target gene ABCG2.” | (6) |
| miR-98 and miR-214  (Oncogenic) | ↑ABCC1, ↑ABCC5  ↑ABCC10 | HCC  HepG2 | In vitro | miR-98 and miR-214/↑ABCC1, ↑ABCC5  ↑ABCC10 | “miR-214 has a negative effect on chemosensitisation by inducing the expression of both ABCC1 and ABCC5. ABCC5 and ABCC10 were downregulated by miR-98 and miR-214 inhibitors.” | (7) |
| miR-205  (Tumor suppressor) | ↓ABCB1(P-gp) | HCC  HepG2 | In vitro | miR-205  /↑PTEN/↓PI3K/ ↓Akt/↓MDR1 | “miR-205  upregulate PTEN to induce cell death and MDR reversal effect by suppression of PI3K/Akt pathway and consequent ABCB1 inhibition.” | (8) |
| miR-338-5p  (Tumor suppressor) | ↓ABCB1(P-gp) | HCC  Hep3B and Huh7 | In vitro | miR-338-5p/↓Epidermal growth factor receptor (EGFR)/↓ Extracellular signal-regulated kinase 1/2 (ERK1/2)/↓ABCB1 | “miR-338-5p  could downregulate ABCB1 expression and sensitize HCC cells to doxorubicin by directly targeting the 3′-untranslated region (3′-UTR) of ABCB1 and even more inhibited ABCB1 expression by targeting the EGFR/ERK1/2 signaling pathway.” | (9) |
| miR-122 (Tumor suppressor) | ↓ABCC1 (MRP1) | HCC  HepG2 | In vitro and in vivo | Chia seeds oil/↑miR-122/↓ABCB1 | “CSO/DOX upregulated miR-122 which inhibits MRP1 and increased apoptosis.” | (10) |
| miR-122  (Tumor suppressor) | ↓ABCB1(P-gp) | HCC  Hep3B, HepG2, Huh7, and PLC/PRF/5 | In vitro | miR-122/ABCB1, GST-π, MRP, Bcl-w, and cyclin B1 | “miR-122 could modulate the sensitivity of the HCC cells to chemotherapeutic drugs through downregulating MDR-related genes MDR-1, GST-π, and MRP, antiapoptotic gene Bcl-w and cell cycle-related gene cyclin B1.” | (11) |
| **Breast cancer** | | | | | | |
| miR-181b-2-3p  (Tumor suppressor) | ↓ABCC3 | Triple-negative breast cancer (TNBC)  MDA-MB-231, and 293 T | In vitro and in vivo | Curcumol/↑NFAT1/↑miR-181b-2-3p/↓ABCC3 | “NFAT1 could be activated by curcumol which directly binds to the promoter region of miR-181b-2-3p, leading to suppression of ABCC3 expression caused by targeting its 3′UTR.” | (12) |
| miR-145  (Tumor suppressor) | ↓ABCC1 (MRP1) | MCF-7, MDA-MB-231, MDA-MB-453, MDA-MB-468, MCF-10A, MDA-kb2 | In vitro and in vivo | miR-145/ ↓ABCC1 | “MRP1 was negatively regulated at the posttranscriptional level by miR-145 through a specific target motif at nt 1728-1734 of the MRP1 3′UTR.” | (13) |
| miR-12136  (Tumor suppressor) | ↓ABCB1(P-gp) | MCF-7 | In vitro | miR-12136/↓ABCB1 | “miR-12136 can directly bind to the 3′-UTR region of ABCB1 mRNA, reduce ABCB1 level and increase Dox drug sensitivity of breast cancer cells.” | (14) |
| microRNA-451  (Tumor suppressor) | ↓ABCB1(P-gp) | Breast adenocarcinoma  MCF-7 | In vitro | microRNA-451/↓ABCB1 | “Enforced increase of miR-451 levels in the MCF-7/DOX cells down-regulates expression of P-gp and increases the sensitivity of the MCF-7-resistant cancer cells to doxorubicin.” | (15) |
| miR-140-5p  (Tumor suppressor) | ↓ABCB1(P-gp) | MCF-7 and MDA-MB-231 | In vitro and in vivo | miR-140-5p/ ↓Wnt1/↓ABCB1 | “miR-140-5p increases the breast cancer sensitivity to Dox is achieved by reducing the expression of Wnt1/ABCB1 signaling.” | (16) |
| miR-186-5p  (Tumor suppressor) | ↓ABCC1 | TNBC  MDA-MB-468 and MDA-MB-436 | In vitro | ↓Zinc finger E-box binding homeobox 1 antisense RNA 1 (ZEB1-AS1)/ ↑miR-186-5p/↓ABCC1 | “ZEB1-AS1 served as an endogenous sponge of miR-186-5p to activate ABCC1 through the ceRNA pattern and ursolic acid relied on ZEB1-AS1-mediated miR-186-5p/ABCC1 signaling to hamper the growth and DOX resistance of TNBC cells.” | (17) |
| miR-221-3p  (Oncogenic) | ↑ABCB1(P-gp) | MCF-7 | In vitro and in vivo | lncRNA GAS5/↓miR-221-3p/↑dickkopf 2 (DKK2)/ ↑c-Myc, ↑cyclin D1 and ↓ABCB1 | “GAS5 inhibits ABCB1 expression by activating the Wnt/β-catenin signaling pathway through the GAS5/miR-221-3p/DKK2 axis.” | (18) |
| miR-200c  (Tumor suppressor) | ↓ABCB1(P-gp) | MDA-MB-231 | In vitro | miR-200c /↓ABCB1 | “miR-200c enhances the breast cancer cells' sensitivity to doxorubicin through downregulating the ABCB1 expression.” | (19) |
| miR-132 and miR-212  (Oncogenic) | ↑ABCG2(BCRP) | MCF-7 | In vitro | miR-132/-212/↓PTEN/↑AKT/↑NF-κB/↑BCRP | “up-regulation of miR-132/-212 in MCF-7/ADR cells suppressed the expression of PTEN, a target gene of miR-132/-212, which activated AKT phosphorylation and the NF-κB pathway and led to increased breast cancer resistance protein expression.” | (20) |
| miR-760  (Tumor suppressor) | ↓ABCA1 | MCF-7 | In vitro | miR-760  /↓ABCA1 | “The miR-760 negatively regulated the expression of ABCA1 in Adriamycin resistant MCF-7 cells.” | (21) |
| miR-298  (Tumor suppressor) | ↓ABCB1(P-gp) | MDA-MB-231 and MCF-7 | In vitro | miR-298  /↓ABCB1 | “miR-298 directly bound to the MDR1 3′ untranslated region and downregulated the expression of the firefly luciferase reporter in a dose-dependent manner.” | (22) |
| miR-134  (Tumor suppressor) | ↓ABCC1(MRP1) | MCF-7 | In vitro | miR-134/ ↓ABCC1 | “microRNA-134 modulates resistance to doxorubicin in breast cancer cells by downregulating the expression of ABCC1 which encodes the MRP1.” | (23) |
| miRNA-34a-5p  (Tumor suppressor) | ↓ABCC1(MRP1) | MCF-7 and MDA-MB-231 | In vitro | miRNA-34a-5p/ ↓ABCC1 | “miRNA-34a-5p restoration as an anti-drug-resistant molecule that highly significantly attenuates the expression of ABCC1 via the direct targeting of its 3′- untranslated regions.” | (24) |
| miR-203 and miR-200c  (Tumor suppressor) | ↓ABCB1 | MCF-7 | In vitro | miR-203 and miR-200c/↓ABCB1 | “miR-200c and miR-203 exert a negative modulating effect on the activity of ABCB1 associated with promoted cytotoxic effects of doxorubicin.” | (25) |
| miR-128 (Tumor suppressor) | ↓ABCC5 | breast tumor-initiating cells (BT-IC)  SKBR3 | In vitro | miR-128/↓ABCC5 | “Reduction in miR-128 leading to Bmi-1 and ABCC5 overexpression is a stem cell–like feature of BT-ICs, which contributes to chemotherapeutic resistance in breast cancers.” | (26) |
| miR-451  (Tumor suppressor) | ↓ABCB1(P-gp), ↓ABCG2(BCRP), and  ↓ABCC1(MRP1) | MCF-7 | In vitro and in vivo | 7-O-geranylquercetin (GQ), miR-451/↓ABCB1, ↓ABCG2, and  ↓ABCC1 | “miR-451 attenuated the expression of transporters MRP1, BCRP and P-gp by suppressing the expression of MRP1, BCRP and MDR1 genes in ADR-resistant cells.” | (27) |
| miR-302  (Tumor suppressor) | ↓ABCB1(P-gp) | MCF-7 | In vitro | miR-302  /↓MEKK1/↓ERK/↓ABCB1 | “miR-302 cooperatively sensitizes breast cancer cells to adriamycin via combinatorial effects on MKEE1 repression and MEKK1-mediated ERK pathway which consequently suppressed P-glycoprotein expression.” | (28) |
| miR-4282  (Tumor suppressor) | ↓ABCC4  ↓ABCB1(P-gp) | MCF-7 | In vitro | miR-4284/↓ABCC4, ↓ABCB1/↓p53 | “MiR-4282 overexpression could prominently inhibit ABCC4 activity and suppress proliferation, invasion and migration of MCF-7-ADR.” | (29) |
| miR‑93  (Tumor suppressor) | ↓ABCB1(P-gp) | MCF-7 | In vitro | miR-4284/ ↓ABCB1, ↓Bcl‑2 | “miR‑93 can increase the apoptosis of MCF‑7/ADM cells and their resistance to adriamycin by inhibiting the expression of Bcl‑2 and P‑gp proteins.” | (30) |
| miR-328-3p  (Tumor suppressor) | ↓ABCG2(BCRP) | TNBC  MDA-MB-231 | In vitro | Dihydrotestosterone/ | “Dihydrotestosterone induced cell sensitivity towards doxorubicin via increasing levels of miR-328-3p and, consequently, reducing ABCG2 levels.” | (31) |
| miR-221-3p  (Oncogenic) | ↑ABCB1(P-gp/MDR1) ↑ABCG2(BCRP) | MCF-7 | In vitro and in vivo | METTL3/↑miR-221-3p/↓HIPK2/↑Che-1 | “METTL3 was shown to stimulate the expression of miR-221-3p by increasing pri-miR-221-3p m6A mRNA methylation, thereby promoting the IC50 value of ADR-resistant MCF-7 cells, facilitating the expression of MDR1 and BCRP, and apoptosis.” | (32) |
| miR-199a  (Tumor suppressor) | ↑ABCC1(MRP1) | MCF-7 | In vitro and in vivo | linc00518/ ↓miR-199a/ ↑ABCC1 | “linc00518 could act as a molecular sponge of miR-199a to repress MRP1 expression.” | (33) |
| **Gastrointestinal cancers** | | | | | | |
| miR‑522  (Tumor suppressor) | ↓ABCB5 | Colon cancer  HT29 | In vitro | miR‑522  /↓ABCB5 | “miR-522 bound to the specific sites of the ABCB5 mRNA 3′UTR and negatively regulated the gene expression of ABCB5.” | (34) |
| miR-495  (Tumor suppressor) | ↓ABCB1(P-gp) | Gastric  SGC7901R | In vitro and in vivo | miR-495/↓ABCB1 | “The reduced expression of MDR1 via the complementary binding of miR-495 to the mRNA of MDR1 could decrease drug efflux from the cell, improve the chemotherapeutic effect, and reverse MDR in cancer.” | (35) |
| miR-508-5p  (Tumor suppressor) | ↓ABCB1(P-gp) | Gastric  SGC7901 | In vitro and in vivo | miR-508-5p/Zinc ribbon domain-containing 1 (ZNRD1)/ABCB1 | “t miR-508-5p could directly target the 3-untranslated regions of ABCB1 and Zinc ribbon domain-containing 1 (ZNRD1), and suppress their expression at the mRNA and protein levels.” | (36) |
| miR-29a  (Tumor suppressor) | ↓ABCB1(P-gp) | Colon cancer  HT29 | In vitro | miR-29a/↑PTEN/↓PI3K/Akt/↓ABCB1 | “miR-29a promoted the expression of PTEN in HT29/DOX cells which results in suppression of the PI3K/Akt signaling pathway; and finally, downregulate P-gp to enhance drug accumulation in doxorubicin-resistant cells.” | (37) |
| miR-107  (Tumor suppressor) | ↓ABCB1(P-gp) | Gastric  MKN-28 and MKN-45 | In vitro and in vivo | Lin28/↓miR-107/↑ABCB1, ↑C-myc, ↓Cylin D1 | “Lin28 significantly downregulated the expression miR-107 to alleviate protein expression of C-myc and P-gp while increasing the Cyclin D1 expression.” | (38) |
| miR-19a/b  (Oncogenic) | ↑ABCB1(P-gp) | Gastric adenocarcinoma  SGC7901 | In vitro | miR-19a/b/↓PTEN/↑Akt/↑ABCB1, ↑Bcl-2 and ↓Bax | “miR-19a/b accelerated the doxorubicin efflux of gastric cancer cells by increasing the levels of mdr1 and P-gp and inhibited drug-induced apoptosis by regulating Bcl-2 and Bax through the suppression of PTEN, as an inhibitor of AKT phosphorylation.” | (39) |
| miR-944  (Tumor suppressor) | ↓ABCC1 (MRP1), ↓ABCB1 (MDR1) | CRC  LoVo and HCT116 | In vitro and in vivo | circCSPP1/↓miR-944/↑FZD7/ ↑ABCC1, ↑ABCB1, ↑Lung resistance protein (LRP) | “circCSPP1 directly downregulated miR-944 expression and miR-944 decreased FZD7 level through targeting to 3′ UTR of FZD7 leading to the increment of protein level of MRP1, P-gp and LRP.” | (40) |
| **Leukemia** | | | | | | |
| miR-9  (Tumor suppressor) | ↓ABCB1(P-gp) | chronic myelogenous leukemia (CML)  K562 | In vitro and in vivo | miR-9/↓ABCB1 | “miR-9 negatively regulates ABCB1 expression to enhance the cytotoxic effects of doxorubicin.” | (41) |
| miR‑145  (Tumor suppressor) | ↓ABCE1(MRP1) | K562 | In vitro | miR‑145/↓ABCE1 | “miR-145 suppressed MRP1 expression by targeting MRP1 3′-UTR, and miR-145 overexpression sensitized K562 cancer cells to ADM by inducing intracellular ADM accumulation via MRP1 inhibition.” | (42) |
| microRNA let-7f  (Tumor suppressor) | ↓ABCC5, ↓ABCC10 | K562 | In vitro and in vivo | microRNA let-7f/↓ABCC5, ↓ABCC10/  ↑p27, ↓cyclin D1, and ↓p‐pRb,  ↓caspase3, PARP, ↑cleaved caspase3, and ↑cleaved PARP | “Upregulation of let‐7f reduced the resistance of leukemia cells to doxorubicin by downregulating MRP (ABCC5 and ABCC10), promoting cell apoptosis, and hindering cell cycle progression. moreover, overexpression of ABCC5 and ABCC10 inhibited cell apoptosis and promoted cell proliferation” | (43) |
| miR-1246  (Tumor suppressor) | ↓ABCB1(P-gp) | K562, and HL-60 | In vitro and in vivo | miR-1246/ ↓AXIN2, ↓GSK-3β/↓β-catenin, ↓Wnt2, ↓c-Myc, ↑APC/↓ABCB1, | "miR-1246 could inhibit the expression of AXIN2 and GSK-3β by directly binding to the 3'UTR seed-matching sites and ultimately suppress the activity of P-gp.” | (44) |
| miR-331–5p and miR-27a  (Tumor suppressor) | ↓ABCB1(P-gp) | K562, and HL60 | In vitro | miR-331–5p and miR-27a | “transfection of K562-resistant cells with miR-27a or miR-331–5p, or a combination of miR-27a and miR-331–5p, resulted in a decrease in P-gp levels.” | (45) |
| **Multiple myeloma** | | | | | | |
| miR-451  (Oncogenic) | ↑ABCB1(MDR1) | NCI-H929, RPMI 8226, KMS-11, LP-1, U266, and SKO | In vitro | miR-451/ ↓tuberous sclerosis 1 (TSC1)/ ↑PIK3/AKT/mTOR/↑ABCB1 | “miR-451 targeted 3′UTR of TSC1, led to a decrease in transcription TSC1, and activation PIK3/AKT/mTOR signaling pathway wich in turne abrogated cells apoptosis, enhanced clonogenicity, and promoted MDR1 mRNA expression.” | (46) |
| **Lymphoma** | | | | | | |
| miR-21  (Oncogenic) | ↑ABCB1(MDR1) | Diffuse large B-cell lymphoma (DLBCL) | In vitro | miR-21/↓PTEN/↑PIK3/AKT/mTOR/↑ABCB1 | “miR-21 downregulated PTEN expression and activated the PI3K/AKT/mTOR pathway, to augment expression and activity of MDR1, thereby leading to resistance of DLBCL cells to doxorubicin.” | (47) |
| **Prostate** | | | | | | |
| miR-21  (Oncogenic) | ↑ABCB1(P-gp) | PC3 | In vitro | miR-21/↓PTEN/↑ABCB1 | “PTEN is a key modulator of the PI3K/Akt/P-gp cascade, which miR-21 suppression led to the upregulation of PTEN and sequentially lower-expression of P-gp that reversed MDR.” | (48) |
| **Lung cancer** | | | | | | |
| miR-199a-5p  (Tumor suppressor) | ↓ABCC1 | Non-small cell lung cancer (NSCLC)  A549 and H460 | In vitro | miR-199a-5p  /↓ABCC1, HIF-1α | “miR-199a-5p downregulated the expression of ABCC1 and HIF-1α were involved in Doxorubicin resistance of NSCLC.” | (49) |
| miR-299-3p (Tumor suppressor) | ↓ABCE1 | Small cell lung cancer (SCLC)  H69 | In vitro | miR-299-3p/↓ABCE1 | “miR-299-3p promotes the sensibility of lung cancer to doxorubicin through suppression of ABCE1.” | (50) |
| **Ovarian cancer** | | | | | | |
| miR-206 (Tumor suppressor) | ↓ABCG2(BCRP) | Ovarian carcinoma cell line SKOV3 | In vitro and in vivo | ↓Nuclear factor erythroid 2-related factor 2 (NRF2)/miR-206/↓Hepatocyte growth factor receptor (HGFR/c-MET), ↓EGFR/↓ABCG2 | “Interfering RNA-induced stable inhibition of NRF2 induced miR-206 to decreased c-MET and EGFR levels through a direct binding to the 3′-untranslated region of the c-MET and EGFR genes, leading to suppression of cancer cell proliferation and BCRP protein levels.” | (51) |
| **Bone cancer** | | | | | | |
| miR-506-3p  (Tumor suppressor) | ↓ABCC1 (MRP1), ↓ABCB1 (MDR1) | Osteosarcoma  U-2OS | In vitro | miR-506-3p /↓JAK2/↓STAT3/ ↓MRP1, ↓MDR1, ↓survivin and ↓Bcl-2 | “miR-506-3p could directly bound to the 3′-UTR of STAT3 mRNA, decrease the protein expression levels of p-JAK2 and finally inhibit the JAK2/STAT3 pathway to hinder MDR1 and MRP1 expression.” | (52) |
| miR-34  (Tumor suppressor) | ↓ABCB1(P-gp) | Osteosarcoma  MG63 | In vitro | Sirolimus/↑miR-34/ ↓PAK1 and ↓ABCB1 | “Two key regulators of the cell cycle, apoptosis and multiple drug resistance, PAK1, and ABCB1, were demonstrated to be the direct targets of miR-34b and sirolimus up-regulated miR-34b to increase the sensitivity of doxorbicin resistance cancer cells.” | (53) |
| miR-137  (Tumor suppressor) | ↓ABCC1 (MRP1), ↓ABCB1(P-gp) | KHOS and U2OS | In vitro and in vivo | circPVT1/↓miR-137/↑TRIAP1/ ↑Bcl-2, ↑ABCB1, ↑ABCC1, and ↓caspase-3 | “circPVT1 acted as a sponge of miR-137 to upregulate TRIAP1 expression and resulting in increment of Bcl-2, ABCB1, and MRP-1 and decrement in c-caspase-3 transcription.” | (54) |
| **Fibrosarcoma** | | | | | | |
| miR-197–5p  (Tumor suppressor) | ↓ABCC1 | HT1080 | In vitro | miR-197–5p/ ↓KIAA0101/ ↑p53/↓ABCC1 | “miR-197–5p negatively regulated KIAA0101 expression to upregulate p53 and sensitize HT1080 cells to Doxorubicin by increasing drug influx, possibly due to suppression of ABCC1.” | (55) |
| **Brain cancer** | | | | | | |
| microRNA-127  (Oncogenic) | ↑ABCC1 (MRP1), ↑ABCB1(P-gp) | Glioma cell lines  U251 and U87-MG | In vitro | microRNA-127/↑Akt/ ↑ABCC1, ↑ABCB, ↑Runx2, ↓p53, ↑bcl-2, ↑ErbB4, and ↑survivin | “Down-regulating microRNA-127 decreased the level of phosphorylated-Akt triggered apoptosis and overcome drug resistance of gliomas cells via suppressing the protein expression levels of MDR1 and MRP1.” | (56) |
| **Renal cancer** | | | | | | |
| miR-210-3p  (Tumor suppressor) | ↓ABCC1 (MRP1) | Caki-2 | In vitro and in vivo | miR-210-3p/↓ABCC1 | “miR-210-3p has a binding site on the 3′UTR of ABCC1 and negatively regulated its expression.” | (57) |
| miR-124  (Tumor suppressor) | ↓ABCB1(P-gp) | Caki-2 | In vitro | miR-124/ ↓frizzled class receptor 5(FZD5)/ ↓protein kinase C (PKC)/ ↓ABCB1 | “miR-124 targeted FZD5 and abolished its presentation, which in turn led to inactivation of PKC and eventually repressed P-gp expression.” | (58) |
| miR-206 (Tumor suppressor) | ↓ABCG2(BCRP) | Renal carcinoma  A498 | In vitro and in vivo | ↓Nuclear factor erythroid 2-related factor 2 (NRF2)/miR-206/↓Hepatocyte growth factor receptor (HGFR/c-MET), ↓EGFR/↓ABCG2 | “Interfering RNA-induced stable inhibition of NRF2 induced miR-206 to decreased c-MET and EGFR levels through a direct binding to the 3′-untranslated region of the c-MET and EGFR genes, leading to suppression of cancer cell proliferation and BCRP protein levels.” | (51) |
| **Retinoblastoma** | | | | | | |
| miR-3163  (Tumor suppressor) | ↓ABCG2(BCRP) | Retinoblastoma cancer stem cells  WERI-Rb1 | In vitro | miR-3163/↓ABCG2 | “ABCG2 expression decreased significantly upon overexpression of miR-3163. miR-3163 gain-of-function led to anti-proliferation and promotion of apoptosis in RCSCs.” | (59) |
| **Neuroblastoma** | | | | | | |
| miR-137  (Tumor suppressor) | ↓ABCB1 (MDR1) | Neuroblastoma  UKF-NB3 | In vitro and in vivo | Constitutive androstane receptor (CAR)/ ↓miR-137/↑MDR1 | “Hypermethylation of the miR-137 promoter and negative regulation of miR-137 by CAR contribute in part to reduced miR-137 expression and increased CAR and MDR1 expression in doxorubicin-resistant neuroblastoma cells.” | (60) |

1. Yang T, Zheng Z-m, Li X-n, Li Z-f, Wang Y, Geng Y-f, et al. MiR-223 modulates multidrug resistance via downregulation of ABCB1 in hepatocellular carcinoma cells. Experimental Biology and Medicine. 2013;238(9):1024-32.

2. Zhao Y, Qi X, Chen J, Wei W, Yu C, Yan H, et al. The miR-491-3p/Sp3/ABCB1 axis attenuates multidrug resistance of hepatocellular carcinoma. Cancer Letters. 2017;408:102-11.

3. Ma J, Wang T, Guo R, Yang X, Yin J, Yu J, et al. Involvement of miR-133a and miR-326 in ADM resistance of HepG2 through modulating expression of ABCC1. Journal of Drug Targeting. 2015;23(6):519-24.

4. Yahya SMM, Fathy SA, El-Khayat ZA, El-Toukhy SE, Hamed AR, Hegazy MGA, et al. Possible Role of microRNA-122 in Modulating Multidrug Resistance of Hepatocellular Carcinoma. Indian Journal of Clinical Biochemistry. 2018;33(1):21-30.

5. Zheng SZ, Sun P, Wang JP, Liu Y, Gong W, Liu J. MiR-34a overexpression enhances the inhibitory effect of doxorubicin on HepG2 cells. World J Gastroenterol. 2019;25(22):2752-62.

6. Xie Z-Y, Liu M-S, Zhang C, Cai P-C, Xiao Z-H, Wang F-F. Aspirin enhances the sensitivity of hepatocellular carcinoma side population cells to doxorubicin via miR-491/ABCG2. Bioscience Reports. 2018;38(6):BSR20180854.

7. Hamed AR, Emara M, Soltan MM, Yahya SMM, Nabih HK, Elsayed GH. Investigating the role of miRNA-98 and miRNA-214 in chemoresistance of HepG2/Dox cells: studying their effects on predicted ABC transporters targets. Medicinal Chemistry Research. 2018;27(2):531-7.

8. Li M, Li ZH, Song J, Li X, Zhai P, Mu X, et al. miR-205 Reverses MDR-1 Mediated Doxorubicin Resistance via PTEN in Human Liver Cancer HepG2 Cells. Cell J. 2022;24(3):112-9.

9. Zhao Y, Chen J, Wei W, Qi X, Li C, Ren J. The dual-inhibitory effect of miR-338-5p on the multidrug resistance and cell growth of hepatocellular carcinoma. Signal Transduction and Targeted Therapy. 2018;3(1):3.

10. Tawfik SA, Awad ET, Abu Bakr HO, Ahmed IM, Ashour E, Gamal-Eldeen AM. Chia Seeds Oil Suppresses the Resistance of Hepatocellular Carcinoma Cells to Liposomal-doxorubicin and Upregulates the Tumor Suppressor miRNAs. Curr Pharm Biotechnol. 2023;24(4):570-8.

11. Xu Y, Xia F, Ma L, Shan J, Shen J, Yang Z, et al. MicroRNA-122 sensitizes HCC cancer cells to adriamycin and vincristine through modulating expression of MDR and inducing cell cycle arrest. Cancer Letters. 2011;310(2):160-9.

12. Zeng C, Fan D, Xu Y, Li X, Yuan J, Yang Q, et al. Curcumol enhances the sensitivity of doxorubicin in triple-negative breast cancer via regulating the miR-181b-2-3p-ABCC3 axis. Biochemical Pharmacology. 2020;174:113795.

13. Gao M, Miao L, Liu M, Li C, Yu C, Yan H, et al. miR-145 sensitizes breast cancer to doxorubicin by targeting multidrug resistance-associated protein-1. Oncotarget. 2016;7(37):59714-26.

14. Yuan J, Xiao C, Lu H, Yu H, Hong H, Guo C, et al. Effect of miR-12136 on Drug Sensitivity of Drug-Resistant Cell Line Michigan Cancer Foundation-7/Doxorubicin by Regulating ATP Binding Cassette Subfamily B Member 1. Journal of Biomaterials and Tissue Engineering. 2020;10(10):1431-5.

15. Kovalchuk O, Filkowski J, Meservy J, Ilnytskyy Y, Tryndyak VP, Chekhun VF, et al. Involvement of microRNA-451 in resistance of the MCF-7 breast cancer cells to chemotherapeutic drug doxorubicin. Molecular Cancer Therapeutics. 2008;7(7):2152-9.

16. Wu D, Zhang J, Lu Y, Bo S, Li L, Wang L, et al. miR-140-5p inhibits the proliferation and enhances the efficacy of doxorubicin to breast cancer stem cells by targeting Wnt1. Cancer Gene Therapy. 2019;26(3):74-82.

17. Lu Q, Chen W, Ji Y, Liu Y, Xue X. Ursolic Acid Enhances Cytotoxicity of Doxorubicin-Resistant Triple-Negative Breast Cancer Cells via ZEB1-AS1/miR-186-5p/ABCC1 Axis. Cancer Biotherapy and Radiopharmaceuticals. 2021;37(8):673-83.

18. Chen Z, Pan T, Jiang D, Jin L, Geng Y, Feng X, et al. The lncRNA-GAS5/miR-221-3p/DKK2 Axis Modulates ABCB1-Mediated Adriamycin Resistance of Breast Cancer via the Wnt/β-Catenin Signaling Pathway. Mol Ther Nucleic Acids. 2020;19:1434-48.

19. Safaei S, Amini M, Najjary S, Mokhtarzadeh A, Bolandi N, Saeedi H, et al. miR-200c increases the sensitivity of breast cancer cells to Doxorubicin through downregulating MDR1 gene. Experimental and Molecular Pathology. 2022;125:104753.

20. Xie M, Fu Z, Cao J, Liu Y, Wu J, Li Q, et al. MicroRNA-132 and microRNA-212 mediate doxorubicin resistance by down-regulating the PTEN-AKT/NF-κB signaling pathway in breast cancer. Biomedicine & Pharmacotherapy. 2018;102:286-94.

21. Lv J, Fu Z, Shi M, Xia K, Ji C, Xu P, et al. Systematic analysis of gene expression pattern in has-miR-760 overexpressed resistance of the MCF-7 human breast cancer cell to doxorubicin. Biomedicine & Pharmacotherapy. 2015;69:162-9.

22. Bao L, Hazari S, Mehra S, Kaushal D, Moroz K, Dash S. Increased Expression of P-Glycoprotein and Doxorubicin Chemoresistance of Metastatic Breast Cancer Is Regulated by miR-298. The American Journal of Pathology. 2012;180(6):2490-503.

23. Lu L, Ju F, Zhao H, Ma X. MicroRNA-134 modulates resistance to doxorubicin in human breast cancer cells by downregulating ABCC1. Biotechnology Letters. 2015;37(12):2387-94.

24. Yahya SMM, Nabih HK, Elsayed GH, Mohamed SIA, Elfiky AM, Salem SM. Restoring microRNA-34a overcomes acquired drug resistance and disease progression in human breast cancer cell lines via suppressing the ABCC1 gene. Breast Cancer Research and Treatment. 2024;204(1):133-49.

25. Armada A, Gomes BC, Viveiros M, Rueff J, Rodrigues AS. Regulation of ABCB1 activity by microRNA-200c and microRNA-203a in breast cancer cells: the quest for microRNAs' involvement in cancer drug resistance. Cancer Drug Resist. 2019;2(3):897-911.

26. Zhu Y, Yu F, Jiao Y, Feng J, Tang W, Yao H, et al. Reduced miR-128 in Breast Tumor–Initiating Cells Induces Chemotherapeutic Resistance via Bmi-1 and ABCC5. Clinical Cancer Research. 2011;17(22):7105-15.

27. Chen Y, Li X, Shi L, Ma P, Wang W, Wu N, et al. Combination of 7-O-geranylquercetin and microRNA-451 enhances antitumor effect of Adriamycin by reserving P-gp-mediated drug resistance in breast cancer. Aging (Albany NY). 2022;14(17):7156-69.

28. Zhao L, Wang Y, Jiang L, He M, Bai X, Yu L, et al. MiR-302a/b/c/d cooperatively sensitizes breast cancer cells to adriamycin via suppressing P-glycoprotein(P-gp) by targeting MAP/ERK kinase kinase 1 (MEKK1). Journal of Experimental & Clinical Cancer Research. 2016;35(1):25.

29. Zhao J, Jiang G. Effect of Dual-Targeting MiR-4282 and ATP-Binding Cassette Sub-Family C Member 4 on Drug Resistance of Breast Cancer Cells and Its Molecular Mechanism. Journal of Biomaterials and Tissue Engineering. 2020;10:507-11.

30. Wang Q, Su C, Li J, Wei C. Mechanism of the enhancing effects of miR‑93 on resistance of breast cancer MCF‑7 cells to adriamycin. Oncol Lett. 2018;16(3):3779-83.

31. Al-Momany B, Hammad H, Ahram M. Dihydrotestosterone Induces Chemo-Resistance of Triple-Negative Breast MDA-MB-231 Cancer Cells Towards Doxorubicin Independent of ABCG2 and miR-328-3p. Curr Mol Pharmacol. 2021;14(5):860-70.

32. Pan X, Hong X, Li S, Meng P, Xiao F. METTL3 promotes adriamycin resistance in MCF-7 breast cancer cells by accelerating pri-microRNA-221-3p maturation in a m6A-dependent manner. Experimental & Molecular Medicine. 2021;53(1):91-102.

33. Chang L, Hu Z, Zhou Z, Zhang H. Linc00518 Contributes to Multidrug Resistance Through Regulating the MiR-199a/MRP1 Axis in Breast Cancer. Cellular Physiology and Biochemistry. 2018;48(1):16-28.

34. Yang G, Jiang O, Ling D, Jiang X, Yuan P, Zeng G, et al. MicroRNA-522 reverses drug resistance of doxorubicin-induced HT29 colon cancer cell by targeting ABCB5. Mol Med Rep. 2015;12(3):3930-6.

35. Zou Z, Zou R, Zong D, Shi Y, Chen J, Huang J, et al. miR-495 sensitizes MDR cancer cells to the combination of doxorubicin and taxol by inhibiting MDR1 expression. Journal of Cellular and Molecular Medicine. 2017;21(9):1929-43.

36. Shang Y, Zhang Z, Liu Z, Feng B, Ren G, Li K, et al. miR-508-5p regulates multidrug resistance of gastric cancer by targeting ABCB1 and ZNRD1. Oncogene. 2014;33(25):3267-76.

37. Shi X, Valizadeh A, Mir SM, Asemi Z, Karimian A, Majidina M, et al. miRNA-29a reverses P-glycoprotein-mediated drug resistance and inhibits proliferation via up-regulation of PTEN in colon cancer cells. European Journal of Pharmacology. 2020;880:173138.

38. Teng R, Hu Y, Zhou J, Seifer B, Chen Y, Shen J, et al. Overexpression of Lin28 Decreases the Chemosensitivity of Gastric Cancer Cells to Oxaliplatin, Paclitaxel, Doxorubicin, and Fluorouracil in Part via microRNA-107. PLOS ONE. 2015;10(12):e0143716.

39. Wang F, Li T, Zhang B, Li H, Wu Q, Yang L, et al. MicroRNA-19a/b regulates multidrug resistance in human gastric cancer cells by targeting PTEN. Biochemical and Biophysical Research Communications. 2013;434(3):688-94.

40. Xi L, Liu Q, Zhang W, Luo L, Song J, Liu R, et al. Circular RNA circCSPP1 knockdown attenuates doxorubicin resistance and suppresses tumor progression of colorectal cancer via miR-944/FZD7 axis. Cancer Cell International. 2021;21(1):153.

41. Li Y, Zhao L, Li N, Miao Y, Zhou H, Jia L. miR-9 regulates the multidrug resistance of chronic myelogenous leukemia by targeting ABCB1. Oncol Rep. 2017;37(4):2193-200.

42. Wuxiao Z, Wang H, Su Q, Zhou H, Hu M, Tao S, et al. MicroRNA‑145 promotes the apoptosis of leukemic stem cells and enhances drug‑resistant K562/ADM cell sensitivity to adriamycin via the regulation of ABCE1. Int J Mol Med. 2020;46(4):1289-300.

43. Cao YX, Wen F, Luo ZY, Long XX, Luo C, Liao P, et al. Downregulation of microRNA let-7f mediated the Adriamycin resistance in leukemia cell line. J Cell Biochem. 2020;121(10):4022-33.

44. Xie B, Li L, Zhang Z, Zhao L, Cheng J, Zhou C, et al. MicroRNA-1246 by Targeting AXIN2 and GSK-3β Overcomes Drug Resistance and Induces Apoptosis in Chemo-resistant Leukemia Cells. J Cancer. 2021;12(14):4196-208.

45. Feng D-D, Zhang H, Zhang P, Zheng Y-S, Zhang X-J, Han B-W, et al. Down-regulated miR-331–5p and miR-27a are associated with chemotherapy resistance and relapse in leukaemia. Journal of Cellular and Molecular Medicine. 2011;15(10):2164-75.

46. Du J, Liu S, He J, Liu X, Qu Y, Yan W, et al. MicroRNA-451 regulates stemness of side population cells via PI3K/Akt/mTOR signaling pathway in multiple myeloma. Oncotarget. 2015;6(17):14993-5007.

47. Go H, Jang JY, Kim PJ, Kim YG, Nam SJ, Paik JH, et al. MicroRNA-21 plays an oncogenic role by targeting FOXO1 and activating the PI3K/AKT pathway in diffuse large B-cell lymphoma. Oncotarget. 2015;6(17):15035-49.

48. Zhao W, Ning L, Wang L, Ouyang T, Qi L, Yang R, et al. miR-21 inhibition reverses doxorubicin-resistance and inhibits PC3 human prostate cancer cells proliferation. Andrologia. 2021;53(5):e14016.

49. Jin Y, Wang H, Zhu Y, Feng H, Wang G, Wang S. miR-199a-5p is involved in doxorubicin resistance of non-small cell lung cancer (NSCLC) cells. European Journal of Pharmacology. 2020;878:173105.

50. Zheng D, Dai Y, Wang S, Xing X. MicroRNA-299-3p promotes the sensibility of lung cancer to doxorubicin through directly targeting ABCE1. Int J Clin Exp Pathol. 2015;8(9):10072-81.

51. Choi BH, Ryu DY, Ryoo IG, Kwak MK. NFE2L2/NRF2 silencing-inducible miR-206 targets c-MET/EGFR and suppresses BCRP/ABCG2 in cancer cells. Oncotarget. 2017;8(63):107188-205.

52. Wang X, Ding R, Fu Z, Yang M, Li D, Zhou Y, et al. Overexpression of miR-506-3p reversed doxorubicin resistance in drug-resistant osteosarcoma cells. Frontiers in Pharmacology. 2024;15.

53. Zhou Y, Zhao R-h, Tseng K-F, Li K-p, Lu Z-g, Liu Y, et al. Sirolimus induces apoptosis and reverses multidrug resistance in human osteosarcoma cells in vitro via increasing microRNA-34b expression. Acta Pharmacologica Sinica. 2016;37(4):519-29.

54. Li D, Huang Y, Wang G. Circular RNA circPVT1 Contributes to Doxorubicin (DXR) Resistance of Osteosarcoma Cells by Regulating TRIAP1 via miR-137. BioMed Research International. 2021;2021(1):7463867.

55. Jain N, Das B, Mallick B. miR-197-5p increases Doxorubicin-mediated anticancer cytotoxicity of HT1080 fibrosarcoma cells by decreasing drug efflux. DNA Repair. 2022;109:103259.

56. Feng R, Dong L. Knockdown of microRNA-127 reverses adriamycin resistance via cell cycle arrest and apoptosis sensitization in adriamycin-resistant human glioma cells. Int J Clin Exp Pathol. 2015;8(6):6107-16.

57. Li S, Yang J, Wang J, Gao W, Ding Y, Ding Y, et al. Down-regulation of miR-210-3p encourages chemotherapy resistance of renal cell carcinoma via modulating ABCC1. Cell & Bioscience. 2018;8(1):9.

58. Long Q-Z, Du Y-F, Liu X-G, Li X, He D-L. miR-124 represses FZD5 to attenuate P-glycoprotein-mediated chemo-resistance in renal cell carcinoma. Tumor Biology. 2015;36(9):7017-26.

59. Jia M, Wei Z, Liu P, Zhao X. Silencing of ABCG2 by microRNA-3163 inhibits multidrug resistance in retinoblastoma cancer stem cells. Journal of Korean medical science. 2016;31(6):836-42.

60. Takwi AA, Wang YM, Wu J, Michaelis M, Cinatl J, Chen T. miR-137 regulates the constitutive androstane receptor and modulates doxorubicin sensitivity in parental and doxorubicin-resistant neuroblastoma cells. Oncogene. 2014;33(28):3717-29.
